# Measurement of double-differential cross sections for top quark pair production in pp collisions at $$\sqrt{s} = 8$$$$\,\text {TeV}$$ and impact on parton distribution functions

**DOI:** 10.1140/epjc/s10052-017-4984-5

**Published:** 2017-07-11

**Authors:** A. M. Sirunyan, A. Tumasyan, W. Adam, E. Asilar, T. Bergauer, J. Brandstetter, E. Brondolin, M. Dragicevic, J. Erö, M. Flechl, M. Friedl, R. Frühwirth, V. M. Ghete, C. Hartl, N. Hörmann, J. Hrubec, M. Jeitler, A. König, I. Krätschmer, D. Liko, T. Matsushita, I. Mikulec, D. Rabady, N. Rad, B. Rahbaran, H. Rohringer, J. Schieck, J. Strauss, W. Waltenberger, C.-E. Wulz, O. Dvornikov, V. Makarenko, V. Mossolov, J. Suarez Gonzalez, V. Zykunov, N. Shumeiko, S. Alderweireldt, E. A. De Wolf, X. Janssen, J. Lauwers, M. Van De Klundert, H. Van Haevermaet, P. Van Mechelen, N. Van Remortel, A. Van Spilbeeck, S. Abu Zeid, F. Blekman, J. D’Hondt, N. Daci, I. De Bruyn, K. Deroover, S. Lowette, S. Moortgat, L. Moreels, A. Olbrechts, Q. Python, K. Skovpen, S. Tavernier, W. Van Doninck, P. Van Mulders, I. Van Parijs, H. Brun, B. Clerbaux, G. De Lentdecker, H. Delannoy, G. Fasanella, L. Favart, R. Goldouzian, A. Grebenyuk, G. Karapostoli, T. Lenzi, A. Léonard, J. Luetic, T. Maerschalk, A. Marinov, A. Randle-conde, T. Seva, C. Vander Velde, P. Vanlaer, D. Vannerom, R. Yonamine, F. Zenoni, F. Zhang, T. Cornelis, D. Dobur, A. Fagot, M. Gul, I. Khvastunov, D. Poyraz, S. Salva, R. Schöfbeck, M. Tytgat, W. Van Driessche, E. Yazgan, N. Zaganidis, H. Bakhshiansohi, O. Bondu, S. Brochet, G. Bruno, A. Caudron, S. De Visscher, C. Delaere, M. Delcourt, B. Francois, A. Giammanco, A. Jafari, M. Komm, G. Krintiras, V. Lemaitre, A. Magitteri, A. Mertens, M. Musich, K. Piotrzkowski, L. Quertenmont, M. Selvaggi, M. Vidal Marono, S. Wertz, N. Beliy, W. L. Aldá Júnior, F. L. Alves, G. A. Alves, L. Brito, C. Hensel, A. Moraes, M. E. Pol, P. Rebello Teles, E. Belchior Batista Das Chagas, W. Carvalho, J. Chinellato, A. Custódio, E. M. Da Costa, G. G. Da Silveira, D. De Jesus Damiao, C. De Oliveira Martins, S. Fonseca De Souza, L. M. Huertas Guativa, H. Malbouisson, D. Matos Figueiredo, C. Mora Herrera, L. Mundim, H. Nogima, W. L. Prado Da Silva, A. Santoro, A. Sznajder, E. J. Tonelli Manganote, F. Torres Da Silva De Araujo, A. Vilela Pereira, S. Ahuja, C. A. Bernardes, S. Dogra, T. R. Fernandez Perez Tomei, E. M. Gregores, P. G. Mercadante, C. S. Moon, S. F. Novaes, Sandra S. Padula, D. Romero Abad, J. C. Ruiz Vargas, A. Aleksandrov, R. Hadjiiska, P. Iaydjiev, M. Rodozov, S. Stoykova, G. Sultanov, M. Vutova, A. Dimitrov, I. Glushkov, L. Litov, B. Pavlov, P. Petkov, W. Fang, M. Ahmad, J. G. Bian, G. M. Chen, H. S. Chen, M. Chen, Y. Chen, T. Cheng, C. H. Jiang, D. Leggat, Z. Liu, F. Romeo, M. Ruan, S. M. Shaheen, A. Spiezia, J. Tao, C. Wang, Z. Wang, H. Zhang, J. Zhao, Y. Ban, G. Chen, Q. Li, S. Liu, Y. Mao, S. J. Qian, D. Wang, Z. Xu, C. Avila, A. Cabrera, L. F. Chaparro Sierra, C. Florez, J. P. Gomez, C. F. González Hernández, J. D. Ruiz Alvarez, J. C. Sanabria, N. Godinovic, D. Lelas, I. Puljak, P. M. Ribeiro Cipriano, T. Sculac, Z. Antunovic, M. Kovac, V. Brigljevic, D. Ferencek, K. Kadija, B. Mesic, T. Susa, M. W. Ather, A. Attikis, G. Mavromanolakis, J. Mousa, C. Nicolaou, F. Ptochos, P. A. Razis, H. Rykaczewski, M. Finger, M. Finger, E. Carrera Jarrin, A. Ellithi Kamel, M. A. Mahmoud, A. Radi, M. Kadastik, L. Perrini, M. Raidal, A. Tiko, C. Veelken, P. Eerola, J. Pekkanen, M. Voutilainen, J. Härkönen, T. Järvinen, V. Karimäki, R. Kinnunen, T. Lampén, K. Lassila-Perini, S. Lehti, T. Lindén, P. Luukka, J. Tuominiemi, E. Tuovinen, L. Wendland, J. Talvitie, T. Tuuva, M. Besancon, F. Couderc, M. Dejardin, D. Denegri, B. Fabbro, J. L. Faure, C. Favaro, F. Ferri, S. Ganjour, S. Ghosh, A. Givernaud, P. Gras, G. Hamel de Monchenault, P. Jarry, I. Kucher, E. Locci, M. Machet, J. Malcles, J. Rander, A. Rosowsky, M. Titov, A. Abdulsalam, I. Antropov, S. Baffioni, F. Beaudette, P. Busson, L. Cadamuro, E. Chapon, C. Charlot, O. Davignon, R. Granier de Cassagnac, M. Jo, S. Lisniak, P. Miné, M. Nguyen, C. Ochando, G. Ortona, P. Paganini, P. Pigard, S. Regnard, R. Salerno, Y. Sirois, A. G. Stahl Leiton, T. Strebler, Y. Yilmaz, A. Zabi, A. Zghiche, J.-L. Agram, J. Andrea, D. Bloch, J.-M. Brom, M. Buttignol, E. C. Chabert, N. Chanon, C. Collard, E. Conte, X. Coubez, J.-C. Fontaine, D. Gelé, U. Goerlach, A.-C. Le Bihan, P. Van Hove, S. Gadrat, S. Beauceron, C. Bernet, G. Boudoul, C. A. Carrillo Montoya, R. Chierici, D. Contardo, B. Courbon, P. Depasse, H. El Mamouni, J. Fay, L. Finco, S. Gascon, M. Gouzevitch, G. Grenier, B. Ille, F. Lagarde, I. B. Laktineh, M. Lethuillier, L. Mirabito, A. L. Pequegnot, S. Perries, A. Popov, V. Sordini, M. Vander Donckt, P. Verdier, S. Viret, A. Khvedelidze, D. Lomidze, C. Autermann, S. Beranek, L. Feld, M. K. Kiesel, K. Klein, M. Lipinski, M. Preuten, C. Schomakers, J. Schulz, T. Verlage, A. Albert, M. Brodski, E. Dietz-Laursonn, D. Duchardt, M. Endres, M. Erdmann, S. Erdweg, T. Esch, R. Fischer, A. Güth, M. Hamer, T. Hebbeker, C. Heidemann, K. Hoepfner, S. Knutzen, M. Merschmeyer, A. Meyer, P. Millet, S. Mukherjee, M. Olschewski, K. Padeken, T. Pook, M. Radziej, H. Reithler, M. Rieger, F. Scheuch, L. Sonnenschein, D. Teyssier, S. Thüer, V. Cherepanov, G. Flügge, B. Kargoll, T. Kress, A. Künsken, J. Lingemann, T. Müller, A. Nehrkorn, A. Nowack, C. Pistone, O. Pooth, A. Stahl, M. Aldaya Martin, T. Arndt, C. Asawatangtrakuldee, K. Beernaert, O. Behnke, U. Behrens, A. A. Bin Anuar, K. Borras, A. Campbell, P. Connor, C. Contreras-Campana, F. Costanza, C. Diez Pardos, G. Dolinska, G. Eckerlin, D. Eckstein, T. Eichhorn, E. Eren, E. Gallo, J. Garay Garcia, A. Geiser, A. Gizhko, J. M. Grados Luyando, A. Grohsjean, P. Gunnellini, A. Harb, J. Hauk, M. Hempel, H. Jung, A. Kalogeropoulos, O. Karacheban, M. Kasemann, J. Keaveney, C. Kleinwort, I. Korol, D. Krücker, W. Lange, A. Lelek, T. Lenz, J. Leonard, K. Lipka, A. Lobanov, W. Lohmann, R. Mankel, I.-A. Melzer-Pellmann, A. B. Meyer, G. Mittag, J. Mnich, A. Mussgiller, D. Pitzl, R. Placakyte, A. Raspereza, B. Roland, M. Ö. Sahin, P. Saxena, T. Schoerner-Sadenius, S. Spannagel, N. Stefaniuk, G. P. Van Onsem, R. Walsh, C. Wissing, O. Zenaiev, V. Blobel, M. Centis Vignali, A. R. Draeger, T. Dreyer, E. Garutti, D. Gonzalez, J. Haller, M. Hoffmann, A. Junkes, R. Klanner, R. Kogler, N. Kovalchuk, S. Kurz, T. Lapsien, I. Marchesini, D. Marconi, M. Meyer, M. Niedziela, D. Nowatschin, F. Pantaleo, T. Peiffer, A. Perieanu, C. Scharf, P. Schleper, A. Schmidt, S. Schumann, J. Schwandt, J. Sonneveld, H. Stadie, G. Steinbrück, F. M. Stober, M. Stöver, H. Tholen, D. Troendle, E. Usai, L. Vanelderen, A. Vanhoefer, B. Vormwald, M. Akbiyik, C. Barth, S. Baur, C. Baus, J. Berger, E. Butz, R. Caspart, T. Chwalek, F. Colombo, W. De Boer, A. Dierlamm, S. Fink, B. Freund, R. Friese, M. Giffels, A. Gilbert, P. Goldenzweig, D. Haitz, F. Hartmann, S. M. Heindl, U. Husemann, F. Kassel, I. Katkov, S. Kudella, H. Mildner, M. U. Mozer, Th. Müller, M. Plagge, G. Quast, K. Rabbertz, S. Röcker, F. Roscher, M. Schröder, I. Shvetsov, G. Sieber, H. J. Simonis, R. Ulrich, S. Wayand, M. Weber, T. Weiler, S. Williamson, C. Wöhrmann, R. Wolf, G. Anagnostou, G. Daskalakis, T. Geralis, V. A. Giakoumopoulou, A. Kyriakis, D. Loukas, I. Topsis-Giotis, S. Kesisoglou, A. Panagiotou, N. Saoulidou, E. Tziaferi, K. Kousouris, I. Evangelou, G. Flouris, C. Foudas, P. Kokkas, N. Loukas, N. Manthos, I. Papadopoulos, E. Paradas, N. Filipovic, G. Pasztor, G. Bencze, C. Hajdu, D. Horvath, F. Sikler, V. Veszpremi, G. Vesztergombi, A. J. Zsigmond, N. Beni, S. Czellar, J. Karancsi, A. Makovec, J. Molnar, Z. Szillasi, M. Bartók, P. Raics, Z. L. Trocsanyi, B. Ujvari, J. R. Komaragiri, S. Bahinipati, S. Bhowmik, S. Choudhury, P. Mal, K. Mandal, A. Nayak, D. K. Sahoo, N. Sahoo, S. K. Swain, S. Bansal, S. B. Beri, V. Bhatnagar, R. Chawla, U. Bhawandeep, A. K. Kalsi, A. Kaur, M. Kaur, R. Kumar, P. Kumari, A. Mehta, M. Mittal, J. B. Singh, G. Walia, Ashok Kumar, A. Bhardwaj, B. C. Choudhary, R. B. Garg, S. Keshri, A. Kumar, S. Malhotra, M. Naimuddin, K. Ranjan, R. Sharma, V. Sharma, R. Bhattacharya, S. Bhattacharya, K. Chatterjee, S. Dey, S. Dutt, S. Dutta, S. Ghosh, N. Majumdar, A. Modak, K. Mondal, S. Mukhopadhyay, S. Nandan, A. Purohit, A. Roy, D. Roy, S. Roy Chowdhury, S. Sarkar, M. Sharan, S. Thakur, P. K. Behera, R. Chudasama, D. Dutta, V. Jha, V. Kumar, A. K. Mohanty, P. K. Netrakanti, L. M. Pant, P. Shukla, A. Topkar, T. Aziz, S. Dugad, G. Kole, B. Mahakud, S. Mitra, G. B. Mohanty, B. Parida, N. Sur, B. Sutar, S. Banerjee, R. K. Dewanjee, S. Ganguly, M. Guchait, Sa. Jain, S. Kumar, M. Maity, G. Majumder, K. Mazumdar, T. Sarkar, N. Wickramage, S. Chauhan, S. Dube, V. Hegde, A. Kapoor, K. Kothekar, S. Pandey, A. Rane, S. Sharma, S. Chenarani, E. Eskandari Tadavani, S. M. Etesami, M. Khakzad, M. Mohammadi Najafabadi, M. Naseri, S. Paktinat Mehdiabadi, F. Rezaei Hosseinabadi, B. Safarzadeh, M. Zeinali, M. Felcini, M. Grunewald, M. Abbrescia, C. Calabria, C. Caputo, A. Colaleo, D. Creanza, L. Cristella, N. De Filippis, M. De Palma, L. Fiore, G. Iaselli, G. Maggi, M. Maggi, G. Miniello, S. My, S. Nuzzo, A. Pompili, G. Pugliese, R. Radogna, A. Ranieri, G. Selvaggi, A. Sharma, L. Silvestris, R. Venditti, P. Verwilligen, G. Abbiendi, C. Battilana, D. Bonacorsi, S. Braibant-Giacomelli, L. Brigliadori, R. Campanini, P. Capiluppi, A. Castro, F. R. Cavallo, S. S. Chhibra, G. Codispoti, M. Cuffiani, G. M. Dallavalle, F. Fabbri, A. Fanfani, D. Fasanella, P. Giacomelli, C. Grandi, L. Guiducci, S. Marcellini, G. Masetti, A. Montanari, F. L. Navarria, A. Perrotta, A. M. Rossi, T. Rovelli, G. P. Siroli, N. Tosi, S. Albergo, S. Costa, A. Di Mattia, F. Giordano, R. Potenza, A. Tricomi, C. Tuve, G. Barbagli, V. Ciulli, C. Civinini, R. D’Alessandro, E. Focardi, P. Lenzi, M. Meschini, S. Paoletti, L. Russo, G. Sguazzoni, D. Strom, L. Viliani, L. Benussi, S. Bianco, F. Fabbri, D. Piccolo, F. Primavera, V. Calvelli, F. Ferro, M. R. Monge, E. Robutti, S. Tosi, L. Brianza, F. Brivio, V. Ciriolo, M. E. Dinardo, S. Fiorendi, S. Gennai, A. Ghezzi, P. Govoni, M. Malberti, S. Malvezzi, R. A. Manzoni, D. Menasce, L. Moroni, M. Paganoni, D. Pedrini, S. Pigazzini, S. Ragazzi, T. Tabarelli de Fatis, S. Buontempo, N. Cavallo, G. De Nardo, S. Di Guida, M. Esposito, F. Fabozzi, F. Fienga, A. O. M. Iorio, G. Lanza, L. Lista, S. Meola, P. Paolucci, C. Sciacca, F. Thyssen, P. Azzi, N. Bacchetta, L. Benato, D. Bisello, A. Boletti, R. Carlin, A. Carvalho Antunes De Oliveira, P. Checchia, M. Dall’Osso, P. De Castro Manzano, T. Dorigo, U. Dosselli, U. Gasparini, F. Gonella, S. Lacaprara, M. Margoni, A. T. Meneguzzo, J. Pazzini, N. Pozzobon, P. Ronchese, R. Rossin, F. Simonetto, E. Torassa, S. Ventura, M. Zanetti, P. Zotto, A. Braghieri, F. Fallavollita, A. Magnani, P. Montagna, S. P. Ratti, V. Re, M. Ressegotti, C. Riccardi, P. Salvini, I. Vai, P. Vitulo, L. Alunni Solestizi, G. M. Bilei, D. Ciangottini, L. Fanò, P. Lariccia, R. Leonardi, G. Mantovani, V. Mariani, M. Menichelli, A. Saha, A. Santocchia, K. Androsov, P. Azzurri, G. Bagliesi, J. Bernardini, T. Boccali, R. Castaldi, M. A. Ciocci, R. Dell’Orso, G. Fedi, A. Giassi, M. T. Grippo, F. Ligabue, T. Lomtadze, L. Martini, A. Messineo, F. Palla, A. Rizzi, A. Savoy-Navarro, P. Spagnolo, R. Tenchini, G. Tonelli, A. Venturi, P. G. Verdini, L. Barone, F. Cavallari, M. Cipriani, D. Del Re, M. Diemoz, S. Gelli, E. Longo, F. Margaroli, B. Marzocchi, P. Meridiani, G. Organtini, R. Paramatti, F. Preiato, S. Rahatlou, C. Rovelli, F. Santanastasio, N. Amapane, R. Arcidiacono, S. Argiro, M. Arneodo, N. Bartosik, R. Bellan, C. Biino, N. Cartiglia, F. Cenna, M. Costa, R. Covarelli, A. Degano, N. Demaria, B. Kiani, C. Mariotti, S. Maselli, E. Migliore, V. Monaco, E. Monteil, M. Monteno, M. M. Obertino, L. Pacher, N. Pastrone, M. Pelliccioni, G. L. Pinna Angioni, F. Ravera, A. Romero, M. Ruspa, R. Sacchi, K. Shchelina, V. Sola, A. Solano, A. Staiano, P. Traczyk, S. Belforte, M. Casarsa, F. Cossutti, G. Della Ricca, A. Zanetti, D. H. Kim, G. N. Kim, M. S. Kim, J. Lee, S. Lee, S. W. Lee, Y. D. Oh, S. Sekmen, D. C. Son, Y. C. Yang, A. Lee, H. Kim, J. A. Brochero Cifuentes, T. J. Kim, S. Cho, S. Choi, Y. Go, D. Gyun, S. Ha, B. Hong, Y. Jo, Y. Kim, K. Lee, K. S. Lee, S. Lee, J. Lim, S. K. Park, Y. Roh, J. Almond, J. Kim, H. Lee, S. B. Oh, B. C. Radburn-Smith, S. H. Seo, U. K. Yang, H. D. Yoo, G. B. Yu, M. Choi, H. Kim, J. H. Kim, J. S. H. Lee, I. C. Park, G. Ryu, M. S. Ryu, Y. Choi, J. Goh, C. Hwang, J. Lee, I. Yu, V. Dudenas, A. Juodagalvis, J. Vaitkus, I. Ahmed, Z. A. Ibrahim, M. A. B. Md Ali, F. Mohamad Idris, W. A. T. Wan Abdullah, M. N. Yusli, Z. Zolkapli, H. Castilla-Valdez, E. De La Cruz-Burelo, I. Heredia-De La Cruz, R. Lopez-Fernandez, R. Magaña Villalba, J. Mejia Guisao, A. Sanchez-Hernandez, S. Carrillo Moreno, C. Oropeza Barrera, F. Vazquez Valencia, S. Carpinteyro, I. Pedraza, H. A. Salazar Ibarguen, C. Uribe Estrada, A. Morelos Pineda, D. Krofcheck, P. H. Butler, A. Ahmad, M. Ahmad, Q. Hassan, H. R. Hoorani, W. A. Khan, A. Saddique, M. A. Shah, M. Shoaib, M. Waqas, H. Bialkowska, M. Bluj, B. Boimska, T. Frueboes, M. Górski, M. Kazana, K. Nawrocki, K. Romanowska-Rybinska, M. Szleper, P. Zalewski, K. Bunkowski, A. Byszuk, K. Doroba, A. Kalinowski, M. Konecki, J. Krolikowski, M. Misiura, M. Olszewski, A. Pyskir, M. Walczak, P. Bargassa, C. Beirão Da Cruz E Silva, B. Calpas, A. Di Francesco, P. Faccioli, M. Gallinaro, J. Hollar, N. Leonardo, L. Lloret Iglesias, M. V. Nemallapudi, J. Seixas, O. Toldaiev, D. Vadruccio, J. Varela, S. Afanasiev, P. Bunin, M. Gavrilenko, I. Golutvin, I. Gorbunov, A. Kamenev, V. Karjavin, A. Lanev, A. Malakhov, V. Matveev, V. Palichik, V. Perelygin, S. Shmatov, S. Shulha, N. Skatchkov, V. Smirnov, N. Voytishin, A. Zarubin, L. Chtchipounov, V. Golovtsov, Y. Ivanov, V. Kim, E. Kuznetsova, V. Murzin, V. Oreshkin, V. Sulimov, A. Vorobyev, Yu. Andreev, A. Dermenev, S. Gninenko, N. Golubev, A. Karneyeu, M. Kirsanov, N. Krasnikov, A. Pashenkov, D. Tlisov, A. Toropin, V. Epshteyn, V. Gavrilov, N. Lychkovskaya, V. Popov, I. Pozdnyakov, G. Safronov, A. Spiridonov, M. Toms, E. Vlasov, A. Zhokin, T. Aushev, A. Bylinkin, M. Danilov, E. Popova, V. Rusinov, V. Andreev, M. Azarkin, I. Dremin, M. Kirakosyan, A. Leonidov, A. Terkulov, A. Baskakov, A. Belyaev, E. Boos, V. Bunichev, M. Dubinin, L. Dudko, A. Ershov, V. Klyukhin, N. Korneeva, I. Lokhtin, I. Miagkov, S. Obraztsov, M. Perfilov, V. Savrin, P. Volkov, V. Blinov, Y. Skovpen, D. Shtol, I. Azhgirey, I. Bayshev, S. Bitioukov, D. Elumakhov, V. Kachanov, A. Kalinin, D. Konstantinov, V. Krychkine, V. Petrov, R. Ryutin, A. Sobol, S. Troshin, N. Tyurin, A. Uzunian, A. Volkov, P. Adzic, P. Cirkovic, D. Devetak, M. Dordevic, J. Milosevic, V. Rekovic, J. Alcaraz Maestre, M. Barrio Luna, E. Calvo, M. Cerrada, M. Chamizo Llatas, N. Colino, B. De La Cruz, A. Delgado Peris, A. Escalante Del Valle, C. Fernandez Bedoya, J. P. Fernández Ramos, J. Flix, M. C. Fouz, P. Garcia-Abia, O. Gonzalez Lopez, S. Goy Lopez, J. M. Hernandez, M. I. Josa, E. Navarro De Martino, A. Pérez-Calero Yzquierdo, J. Puerta Pelayo, A. Quintario Olmeda, I. Redondo, L. Romero, M. S. Soares, J. F. de Trocóniz, M. Missiroli, D. Moran, J. Cuevas, C. Erice, J. Fernandez Menendez, I. Gonzalez Caballero, J. R. González Fernández, E. Palencia Cortezon, S. Sanchez Cruz, I. Suárez Andrés, P. Vischia, J. M. Vizan Garcia, I. J. Cabrillo, A. Calderon, E. Curras, M. Fernandez, J. Garcia-Ferrero, G. Gomez, A. Lopez Virto, J. Marco, C. Martinez Rivero, F. Matorras, J. Piedra Gomez, T. Rodrigo, A. Ruiz-Jimeno, L. Scodellaro, N. Trevisani, I. Vila, R. Vilar Cortabitarte, D. Abbaneo, E. Auffray, G. Auzinger, P. Baillon, A. H. Ball, D. Barney, P. Bloch, A. Bocci, C. Botta, T. Camporesi, R. Castello, M. Cepeda, G. Cerminara, Y. Chen, A. Cimmino, D. d’Enterria, A. Dabrowski, V. Daponte, A. David, M. De Gruttola, A. De Roeck, E. Di Marco, M. Dobson, B. Dorney, T. du Pree, D. Duggan, M. Dünser, N. Dupont, A. Elliott-Peisert, P. Everaerts, S. Fartoukh, G. Franzoni, J. Fulcher, W. Funk, D. Gigi, K. Gill, M. Girone, F. Glege, D. Gulhan, S. Gundacker, M. Guthoff, P. Harris, J. Hegeman, V. Innocente, P. Janot, J. Kieseler, H. Kirschenmann, V. Knünz, A. Kornmayer, M. J. Kortelainen, M. Krammer, C. Lange, P. Lecoq, C. Lourenço, M. T. Lucchini, L. Malgeri, M. Mannelli, A. Martelli, F. Meijers, J. A. Merlin, S. Mersi, E. Meschi, P. Milenovic, F. Moortgat, S. Morovic, M. Mulders, H. Neugebauer, S. Orfanelli, L. Orsini, L. Pape, E. Perez, M. Peruzzi, A. Petrilli, G. Petrucciani, A. Pfeiffer, M. Pierini, A. Racz, T. Reis, G. Rolandi, M. Rovere, H. Sakulin, J. B. Sauvan, C. Schäfer, C. Schwick, M. Seidel, A. Sharma, P. Silva, P. Sphicas, J. Steggemann, M. Stoye, Y. Takahashi, M. Tosi, D. Treille, A. Triossi, A. Tsirou, V. Veckalns, G. I. Veres, M. Verweij, N. Wardle, H. K. Wöhri, A. Zagozdzinska, W. D. Zeuner, W. Bertl, K. Deiters, W. Erdmann, R. Horisberger, Q. Ingram, H. C. Kaestli, D. Kotlinski, U. Langenegger, T. Rohe, S. A. Wiederkehr, F. Bachmair, L. Bäni, L. Bianchini, B. Casal, G. Dissertori, M. Dittmar, M. Donegà, C. Grab, C. Heidegger, D. Hits, J. Hoss, G. Kasieczka, W. Lustermann, B. Mangano, M. Marionneau, P. Martinez Ruiz del Arbol, M. Masciovecchio, M. T. Meinhard, D. Meister, F. Micheli, P. Musella, F. Nessi-Tedaldi, F. Pandolfi, J. Pata, F. Pauss, G. Perrin, L. Perrozzi, M. Quittnat, M. Rossini, M. Schönenberger, A. Starodumov, V. R. Tavolaro, K. Theofilatos, R. Wallny, T. K. Aarrestad, C. Amsler, L. Caminada, M. F. Canelli, A. De Cosa, S. Donato, C. Galloni, A. Hinzmann, T. Hreus, B. Kilminster, J. Ngadiuba, D. Pinna, G. Rauco, P. Robmann, D. Salerno, C. Seitz, Y. Yang, A. Zucchetta, V. Candelise, T. H. Doan, Sh. Jain, R. Khurana, M. Konyushikhin, C. M. Kuo, W. Lin, A. Pozdnyakov, S. S. Yu, Arun Kumar, P. Chang, Y. H. Chang, Y. Chao, K. F. Chen, P. H. Chen, F. Fiori, W.-S. Hou, Y. Hsiung, Y. F. Liu, R.-S. Lu, M. Miñano Moya, E. Paganis, A. Psallidas, J. F. Tsai, B. Asavapibhop, G. Singh, N. Srimanobhas, N. Suwonjandee, A. Adiguzel, F. Boran, S. Cerci, S. Damarseckin, Z. S. Demiroglu, C. Dozen, I. Dumanoglu, S. Girgis, G. Gokbulut, Y. Guler, I. Hos, E. E. Kangal, O. Kara, U. Kiminsu, M. Oglakci, G. Onengut, K. Ozdemir, D. Sunar Cerci, B. Tali, H. Topakli, S. Turkcapar, I. S. Zorbakir, C. Zorbilmez, B. Bilin, S. Bilmis, B. Isildak, G. Karapinar, M. Yalvac, M. Zeyrek, E. Gülmez, M. Kaya, O. Kaya, E. A. Yetkin, T. Yetkin, A. Cakir, K. Cankocak, S. Sen, B. Grynyov, L. Levchuk, P. Sorokin, R. Aggleton, F. Ball, L. Beck, J. J. Brooke, D. Burns, E. Clement, D. Cussans, H. Flacher, J. Goldstein, M. Grimes, G. P. Heath, H. F. Heath, J. Jacob, L. Kreczko, C. Lucas, D. M. Newbold, S. Paramesvaran, A. Poll, T. Sakuma, S. Seif El Nasr-storey, D. Smith, V. J. Smith, K. W. Bell, A. Belyaev, C. Brew, R. M. Brown, L. Calligaris, D. Cieri, D. J. A. Cockerill, J. A. Coughlan, K. Harder, S. Harper, E. Olaiya, D. Petyt, C. H. Shepherd-Themistocleous, A. Thea, I. R. Tomalin, T. Williams, M. Baber, R. Bainbridge, O. Buchmuller, A. Bundock, S. Casasso, M. Citron, D. Colling, L. Corpe, P. Dauncey, G. Davies, A. De Wit, M. Della Negra, R. Di Maria, P. Dunne, A. Elwood, D. Futyan, Y. Haddad, G. Hall, G. Iles, T. James, R. Lane, C. Laner, L. Lyons, A.-M. Magnan, S. Malik, L. Mastrolorenzo, J. Nash, A. Nikitenko, J. Pela, B. Penning, M. Pesaresi, D. M. Raymond, A. Richards, A. Rose, E. Scott, C. Seez, S. Summers, A. Tapper, K. Uchida, M. Vazquez Acosta, T. Virdee, J. Wright, S. C. Zenz, J. E. Cole, P. R. Hobson, A. Khan, P. Kyberd, I. D. Reid, P. Symonds, L. Teodorescu, M. Turner, A. Borzou, K. Call, J. Dittmann, K. Hatakeyama, H. Liu, N. Pastika, R. Bartek, A. Dominguez, A. Buccilli, S. I. Cooper, C. Henderson, P. Rumerio, C. West, D. Arcaro, A. Avetisyan, T. Bose, D. Gastler, D. Rankin, C. Richardson, J. Rohlf, L. Sulak, D. Zou, G. Benelli, D. Cutts, A. Garabedian, J. Hakala, U. Heintz, J. M. Hogan, O. Jesus, K. H. M. Kwok, E. Laird, G. Landsberg, Z. Mao, M. Narain, S. Piperov, S. Sagir, E. Spencer, R. Syarif, R. Breedon, D. Burns, M. Calderon De La Barca Sanchez, S. Chauhan, M. Chertok, J. Conway, R. Conway, P. T. Cox, R. Erbacher, C. Flores, G. Funk, M. Gardner, W. Ko, R. Lander, C. Mclean, M. Mulhearn, D. Pellett, J. Pilot, S. Shalhout, M. Shi, J. Smith, M. Squires, D. Stolp, K. Tos, M. Tripathi, M. Bachtis, C. Bravo, R. Cousins, A. Dasgupta, A. Florent, J. Hauser, M. Ignatenko, N. Mccoll, D. Saltzberg, C. Schnaible, V. Valuev, M. Weber, E. Bouvier, K. Burt, R. Clare, J. Ellison, J. W. Gary, S. M. A. Ghiasi Shirazi, G. Hanson, J. Heilman, P. Jandir, E. Kennedy, F. Lacroix, O. R. Long, M. Olmedo Negrete, M. I. Paneva, A. Shrinivas, W. Si, H. Wei, S. Wimpenny, B. R. Yates, J. G. Branson, G. B. Cerati, S. Cittolin, M. Derdzinski, R. Gerosa, A. Holzner, D. Klein, V. Krutelyov, J. Letts, I. Macneill, D. Olivito, S. Padhi, M. Pieri, M. Sani, V. Sharma, S. Simon, M. Tadel, A. Vartak, S. Wasserbaech, C. Welke, J. Wood, F. Würthwein, A. Yagil, G. Zevi Della Porta, N. Amin, R. Bhandari, J. Bradmiller-Feld, C. Campagnari, A. Dishaw, V. Dutta, M. Franco Sevilla, C. George, F. Golf, L. Gouskos, J. Gran, R. Heller, J. Incandela, S. D. Mullin, A. Ovcharova, H. Qu, J. Richman, D. Stuart, I. Suarez, J. Yoo, D. Anderson, J. Bendavid, A. Bornheim, J. Bunn, J. Duarte, J. M. Lawhorn, A. Mott, H. B. Newman, C. Pena, M. Spiropulu, J. R. Vlimant, S. Xie, R. Y. Zhu, M. B. Andrews, T. Ferguson, M. Paulini, J. Russ, M. Sun, H. Vogel, I. Vorobiev, M. Weinberg, J. P. Cumalat, W. T. Ford, F. Jensen, A. Johnson, M. Krohn, S. Leontsinis, T. Mulholland, K. Stenson, S. R. Wagner, J. Alexander, J. Chaves, J. Chu, S. Dittmer, K. Mcdermott, N. Mirman, J. R. Patterson, A. Rinkevicius, A. Ryd, L. Skinnari, L. Soffi, S. M. Tan, Z. Tao, J. Thom, J. Tucker, P. Wittich, M. Zientek, D. Winn, S. Abdullin, M. Albrow, G. Apollinari, A. Apresyan, S. Banerjee, L. A. T. Bauerdick, A. Beretvas, J. Berryhill, P. C. Bhat, G. Bolla, K. Burkett, J. N. Butler, H. W. K. Cheung, F. Chlebana, S. Cihangir, M. Cremonesi, V. D. Elvira, I. Fisk, J. Freeman, E. Gottschalk, L. Gray, D. Green, S. Grünendahl, O. Gutsche, D. Hare, R. M. Harris, S. Hasegawa, J. Hirschauer, Z. Hu, B. Jayatilaka, S. Jindariani, M. Johnson, U. Joshi, B. Klima, B. Kreis, S. Lammel, J. Linacre, D. Lincoln, R. Lipton, M. Liu, T. Liu, R. Lopes De Sá, J. Lykken, K. Maeshima, N. Magini, J. M. Marraffino, S. Maruyama, D. Mason, P. McBride, P. Merkel, S. Mrenna, S. Nahn, V. O’Dell, K. Pedro, O. Prokofyev, G. Rakness, L. Ristori, E. Sexton-Kennedy, A. Soha, W. J. Spalding, L. Spiegel, S. Stoynev, J. Strait, N. Strobbe, L. Taylor, S. Tkaczyk, N. V. Tran, L. Uplegger, E. W. Vaandering, C. Vernieri, M. Verzocchi, R. Vidal, M. Wang, H. A. Weber, A. Whitbeck, Y. Wu, D. Acosta, P. Avery, P. Bortignon, D. Bourilkov, A. Brinkerhoff, A. Carnes, M. Carver, D. Curry, S. Das, R. D. Field, I. K. Furic, J. Konigsberg, A. Korytov, J. F. Low, P. Ma, K. Matchev, H. Mei, G. Mitselmakher, D. Rank, L. Shchutska, D. Sperka, L. Thomas, J. Wang, S. Wang, J. Yelton, S. Linn, P. Markowitz, G. Martinez, J. L. Rodriguez, A. Ackert, T. Adams, A. Askew, S. Bein, S. Hagopian, V. Hagopian, K. F. Johnson, T. Kolberg, T. Perry, H. Prosper, A. Santra, R. Yohay, M. M. Baarmand, V. Bhopatkar, S. Colafranceschi, M. Hohlmann, D. Noonan, T. Roy, F. Yumiceva, M. R. Adams, L. Apanasevich, D. Berry, R. R. Betts, R. Cavanaugh, X. Chen, O. Evdokimov, C. E. Gerber, D. A. Hangal, D. J. Hofman, K. Jung, J. Kamin, I. D. Sandoval Gonzalez, H. Trauger, N. Varelas, H. Wang, Z. Wu, J. Zhang, B. Bilki, W. Clarida, K. Dilsiz, S. Durgut, R. P. Gandrajula, M. Haytmyradov, V. Khristenko, J.-P. Merlo, H. Mermerkaya, A. Mestvirishvili, A. Moeller, J. Nachtman, H. Ogul, Y. Onel, F. Ozok, A. Penzo, C. Snyder, E. Tiras, J. Wetzel, K. Yi, B. Blumenfeld, A. Cocoros, N. Eminizer, D. Fehling, L. Feng, A. V. Gritsan, P. Maksimovic, J. Roskes, U. Sarica, M. Swartz, M. Xiao, C. You, A. Al-bataineh, P. Baringer, A. Bean, S. Boren, J. Bowen, J. Castle, L. Forthomme, S. Khalil, A. Kropivnitskaya, D. Majumder, W. Mcbrayer, M. Murray, S. Sanders, R. Stringer, J. D. Tapia Takaki, Q. Wang, A. Ivanov, K. Kaadze, Y. Maravin, A. Mohammadi, L. K. Saini, N. Skhirtladze, S. Toda, F. Rebassoo, D. Wright, C. Anelli, A. Baden, O. Baron, A. Belloni, B. Calvert, S. C. Eno, C. Ferraioli, J. A. Gomez, N. J. Hadley, S. Jabeen, G. Y. Jeng, R. G. Kellogg, J. Kunkle, A. C. Mignerey, F. Ricci-Tam, Y. H. Shin, A. Skuja, M. B. Tonjes, S. C. Tonwar, D. Abercrombie, B. Allen, A. Apyan, V. Azzolini, R. Barbieri, A. Baty, R. Bi, K. Bierwagen, S. Brandt, W. Busza, I. A. Cali, M. D’Alfonso, Z. Demiragli, G. Gomez Ceballos, M. Goncharov, D. Hsu, Y. Iiyama, G. M. Innocenti, M. Klute, D. Kovalskyi, K. Krajczar, Y. S. Lai, Y.-J. Lee, A. Levin, P. D. Luckey, B. Maier, A. C. Marini, C. Mcginn, C. Mironov, S. Narayanan, X. Niu, C. Paus, C. Roland, G. Roland, J. Salfeld-Nebgen, G. S. F. Stephans, K. Tatar, D. Velicanu, J. Wang, T. W. Wang, B. Wyslouch, A. C. Benvenuti, R. M. Chatterjee, A. Evans, P. Hansen, S. Kalafut, S. C. Kao, Y. Kubota, Z. Lesko, J. Mans, S. Nourbakhsh, N. Ruckstuhl, R. Rusack, N. Tambe, J. Turkewitz, J. G. Acosta, S. Oliveros, E. Avdeeva, K. Bloom, D. R. Claes, C. Fangmeier, R. Gonzalez Suarez, R. Kamalieddin, I. Kravchenko, A. Malta Rodrigues, J. Monroy, J. E. Siado, G. R. Snow, B. Stieger, M. Alyari, J. Dolen, A. Godshalk, C. Harrington, I. Iashvili, J. Kaisen, D. Nguyen, A. Parker, S. Rappoccio, B. Roozbahani, G. Alverson, E. Barberis, A. Hortiangtham, A. Massironi, D. M. Morse, D. Nash, T. Orimoto, R. Teixeira De Lima, D. Trocino, R.-J. Wang, D. Wood, S. Bhattacharya, O. Charaf, K. A. Hahn, N. Mucia, N. Odell, B. Pollack, M. H. Schmitt, K. Sung, M. Trovato, M. Velasco, N. Dev, M. Hildreth, K. Hurtado Anampa, C. Jessop, D. J. Karmgard, N. Kellams, K. Lannon, N. Marinelli, F. Meng, C. Mueller, Y. Musienko, M. Planer, A. Reinsvold, R. Ruchti, N. Rupprecht, G. Smith, S. Taroni, M. Wayne, M. Wolf, A. Woodard, J. Alimena, L. Antonelli, B. Bylsma, L. S. Durkin, S. Flowers, B. Francis, A. Hart, C. Hill, W. Ji, B. Liu, W. Luo, D. Puigh, B. L. Winer, H. W. Wulsin, S. Cooperstein, O. Driga, P. Elmer, J. Hardenbrook, P. Hebda, D. Lange, J. Luo, D. Marlow, T. Medvedeva, K. Mei, I. Ojalvo, J. Olsen, C. Palmer, P. Piroué, D. Stickland, A. Svyatkovskiy, C. Tully, S. Malik, A. Barker, V. E. Barnes, S. Folgueras, L. Gutay, M. K. Jha, M. Jones, A. W. Jung, A. Khatiwada, D. H. Miller, N. Neumeister, J. F. Schulte, X. Shi, J. Sun, F. Wang, W. Xie, N. Parashar, J. Stupak, A. Adair, B. Akgun, Z. Chen, K. M. Ecklund, F. J. M. Geurts, M. Guilbaud, W. Li, B. Michlin, M. Northup, B. P. Padley, J. Roberts, J. Rorie, Z. Tu, J. Zabel, B. Betchart, A. Bodek, P. de Barbaro, R. Demina, Y. T. Duh, T. Ferbel, M. Galanti, A. Garcia-Bellido, J. Han, O. Hindrichs, A. Khukhunaishvili, K. H. Lo, P. Tan, M. Verzetti, A. Agapitos, J. P. Chou, Y. Gershtein, T. A. Gómez Espinosa, E. Halkiadakis, M. Heindl, E. Hughes, S. Kaplan, R. Kunnawalkam Elayavalli, S. Kyriacou, A. Lath, R. Montalvo, K. Nash, M. Osherson, H. Saka, S. Salur, S. Schnetzer, D. Sheffield, S. Somalwar, R. Stone, S. Thomas, P. Thomassen, M. Walker, A. G. Delannoy, M. Foerster, J. Heideman, G. Riley, K. Rose, S. Spanier, K. Thapa, O. Bouhali, A. Celik, M. Dalchenko, M. De Mattia, A. Delgado, S. Dildick, R. Eusebi, J. Gilmore, T. Huang, E. Juska, T. Kamon, R. Mueller, Y. Pakhotin, R. Patel, A. Perloff, L. Perniè, D. Rathjens, A. Safonov, A. Tatarinov, K. A. Ulmer, N. Akchurin, J. Damgov, F. De Guio, C. Dragoiu, P. R. Dudero, J. Faulkner, E. Gurpinar, S. Kunori, K. Lamichhane, S. W. Lee, T. Libeiro, T. Peltola, S. Undleeb, I. Volobouev, Z. Wang, S. Greene, A. Gurrola, R. Janjam, W. Johns, C. Maguire, A. Melo, H. Ni, P. Sheldon, S. Tuo, J. Velkovska, Q. Xu, M. W. Arenton, P. Barria, B. Cox, R. Hirosky, A. Ledovskoy, H. Li, C. Neu, T. Sinthuprasith, X. Sun, Y. Wang, E. Wolfe, F. Xia, C. Clarke, R. Harr, P. E. Karchin, J. Sturdy, S. Zaleski, D. A. Belknap, J. Buchanan, C. Caillol, S. Dasu, L. Dodd, S. Duric, B. Gomber, M. Grothe, M. Herndon, A. Hervé, U. Hussain, P. Klabbers, A. Lanaro, A. Levine, K. Long, R. Loveless, G. A. Pierro, G. Polese, T. Ruggles, A. Savin, N. Smith, W. H. Smith, D. Taylor, N. Woods, CMS Collaboration

**Affiliations:** 1 Yerevan Physics Institute, Yerevan, Armenia; 2 Institut für Hochenergiephysik, Vienna, Austria; 3 Institute for Nuclear Problems, Minsk, Belarus; 4 National Centre for Particle and High Energy Physics, Minsk, Belarus; 5 Universiteit Antwerpen, Antwerpen, Belgium; 6 Vrije Universiteit Brussel, Brussel, Belgium; 7 Université Libre de Bruxelles, Bruxelles, Belgium; 8 Ghent University, Ghent, Belgium; 9 Université Catholique de Louvain, Louvain-la-Neuve, Belgium; 10 Université de Mons, Mons, Belgium; 11 Centro Brasileiro de Pesquisas Fisicas, Rio de Janeiro, Brazil; 12 Universidade do Estado do Rio de Janeiro, Rio de Janeiro, Brazil; 13 Universidade Estadual Paulista, Universidade Federal do ABC, São Paulo, Brazil; 14 Institute for Nuclear Research and Nuclear Energy, Sofia, Bulgaria; 15 University of Sofia, Sofia, Bulgaria; 16 Beihang University, Beijing, China; 17 Institute of High Energy Physics, Beijing, China; 18 State Key Laboratory of Nuclear Physics and Technology, Peking University, Beijing, China; 19 Universidad de Los Andes, Bogotá, Colombia; 20 Faculty of Electrical Engineering Mechanical Engineering and Naval Architecture, University of Split, Split, Croatia; 21 Faculty of Science, University of Split, Split, Croatia; 22 Institute Rudjer Boskovic, Zagreb, Croatia; 23 University of Cyprus, Nicosia, Cyprus; 24 Charles University, Prague, Czech Republic; 25 Universidad San Francisco de Quito, Quito, Ecuador; 26 Academy of Scientific Research and Technology of the Arab Republic of Egypt, Egyptian Network of High Energy Physics, Cairo, Egypt; 27 National Institute of Chemical Physics and Biophysics, Tallinn, Estonia; 28 Department of Physics, University of Helsinki, Helsinki, Finland; 29 Helsinki Institute of Physics, Helsinki, Finland; 30 Lappeenranta University of Technology, Lappeenranta, Finland; 31 IRFU, CEA, Université Paris-Saclay, Gif-sur-Yvette, France; 32 Laboratoire Leprince-Ringuet, Ecole Polytechnique, IN2P3-CNRS, Palaiseau, France; 33 Institut Pluridisciplinaire Hubert Curien (IPHC), Université de Strasbourg, CNRS-IN2P3, Strasbourg, France; 34 Centre de Calcul de l’Institut National de Physique Nucleaire et de Physique des Particules, CNRS/IN2P3, Villeurbanne, France; 35 Université de Lyon, Université Claude Bernard Lyon 1, CNRS-IN2P3, Institut de Physique Nucléaire de Lyon, Villeurbanne, France; 36 Georgian Technical University, Tbilisi, Georgia; 37 Tbilisi State University, Tbilisi, Georgia; 38 RWTH Aachen University, I. Physikalisches Institut, Aachen, Germany; 39 RWTH Aachen University, III. Physikalisches Institut A, Aachen, Germany; 40 RWTH Aachen University,III. Physikalisches Institut B, Aachen, Germany; 41 Deutsches Elektronen-Synchrotron, Hamburg, Germany; 42 University of Hamburg, Hamburg, Germany; 43 Institut für Experimentelle Kernphysik, Karlsruhe, Germany; 44 Institute of Nuclear and Particle Physics (INPP), NCSR Demokritos, Aghia Paraskevi, Greece; 45 National and Kapodistrian University of Athens, Athens, Greece; 46 National Technical University of Athens, Athens, Greece; 47 University of Ioánnina, Ioannina, Greece; 48 MTA-ELTE Lendület CMS Particle and Nuclear Physics Group, Eötvös Loránd University, Budapest, Hungary; 49 Wigner Research Centre for Physics, Budapest, Hungary; 50 Institute of Nuclear Research ATOMKI, Debrecen, Hungary; 51 Institute of Physics, University of Debrecen, Debrecen, Hungary; 52 Indian Institute of Science (IISc), Bengaluru, India; 53 National Institute of Science Education and Research, Bhubaneswar, India; 54 Panjab University, Chandigarh, India; 55 University of Delhi, Delhi, India; 56 Saha Institute of Nuclear Physics, Kolkata, India; 57 Indian Institute of Technology Madras, Madras, India; 58 Bhabha Atomic Research Centre, Mumbai, India; 59 Tata Institute of Fundamental Research-A, Mumbai, India; 60 Tata Institute of Fundamental Research-B, Mumbai, India; 61 Indian Institute of Science Education and Research (IISER), Pune, India; 62 Institute for Research in Fundamental Sciences (IPM), Tehran, Iran; 63 University College Dublin, Dublin, Ireland; 64 INFN Sezione di Bari, Università di Bari, Politecnico di Bari, Bari, Italy; 65 INFN Sezione di Bologna, Università di Bologna, Bologna, Italy; 66 INFN Sezione di Catania, Università di Catania, Catania, Italy; 67 INFN Sezione di Firenze, Università di Firenze, Florence, Italy; 68 INFN Laboratori Nazionali di Frascati, Frascati, Italy; 69 INFN Sezione di Genova, Università di Genova, Genova, Italy; 70 INFN Sezione di Milano-Bicocca, Università di Milano-Bicocca, Milan, Italy; 71 INFN Sezione di Napoli, Università di Napoli ’Federico II’, Napoli, Italy, Università della Basilicata, Potenza, Italy, Università G. Marconi, Rome, Italy; 72 INFN Sezione di Padova, Università di Padova, Padova, Italy, Università di Trento, Trento, Italy; 73 INFN Sezione di Pavia, Università di Pavia, Pavia, Italy; 74 INFN Sezione di Perugia, Università di Perugia, Perugia, Italy; 75 INFN Sezione di Pisa, Università di Pisa, Scuola Normale Superiore di Pisa, Pisa, Italy; 76 INFN Sezione di Roma, Università di Roma, Rome, Italy; 77 INFN Sezione di Torino, Università di Torino, Torino, Italy, Università del Piemonte Orientale, Novara, Italy; 78 INFN Sezione di Trieste, Università di Trieste, Trieste, Italy; 79 Kyungpook National University, Taegu, Korea; 80 Chonbuk National University, Jeonju, Korea; 81 Chonnam National University, Institute for Universe and Elementary Particles, Kwangju, Korea; 82 Hanyang University, Seoul, Korea; 83 Korea University, Seoul, Korea; 84 Seoul National University, Seoul, Korea; 85 University of Seoul, Seoul, Korea; 86 Sungkyunkwan University, Suwon, Korea; 87 Vilnius University, Vilnius, Lithuania; 88 National Centre for Particle Physics, Universiti Malaya, Kuala Lumpur, Malaysia; 89 Centro de Investigacion y de Estudios Avanzados del IPN, Mexico City, Mexico; 90 Universidad Iberoamericana, Mexico City, Mexico; 91 Benemerita Universidad Autonoma de Puebla, Puebla, Mexico; 92 Universidad Autónoma de San Luis Potosí, San Luis Potosí, Mexico; 93 University of Auckland, Auckland, New Zealand; 94 University of Canterbury, Christchurch, New Zealand; 95 National Centre for Physics, Quaid-I-Azam University, Islamabad, Pakistan; 96 National Centre for Nuclear Research, Swierk, Poland; 97 Faculty of Physics, Institute of Experimental Physics, University of Warsaw, Warsaw, Poland; 98 Laboratório de Instrumentação e Física Experimental de Partículas, Lisbon, Portugal; 99 Joint Institute for Nuclear Research, Dubna, Russia; 100 Petersburg Nuclear Physics Institute, Gatchina (St. Petersburg), Russia; 101 Institute for Nuclear Research, Moscow, Russia; 102 Institute for Theoretical and Experimental Physics, Moscow, Russia; 103 Moscow Institute of Physics and Technology, Moscow, Russia; 104 National Research Nuclear University ‘Moscow Engineering Physics Institute’ (MEPhI), Moscow, Russia; 105 P.N. Lebedev Physical Institute, Moscow, Russia; 106 Skobeltsyn Institute of Nuclear Physics, Lomonosov Moscow State University, Moscow, Russia; 107 Novosibirsk State University (NSU), Novosibirsk, Russia; 108 State Research Center of Russian Federation, Institute for High Energy Physics, Protvino, Russia; 109 Faculty of Physics and Vinca Institute of Nuclear Sciences, University of Belgrade, Belgrade, Serbia; 110 Centro de Investigaciones Energéticas Medioambientales y Tecnológicas (CIEMAT), Madrid, Spain; 111 Universidad Autónoma de Madrid, Madrid, Spain; 112 Universidad de Oviedo, Oviedo, Spain; 113 Instituto de Física de Cantabria (IFCA), CSIC-Universidad de Cantabria, Santander, Spain; 114 CERN, European Organization for Nuclear Research, Geneva, Switzerland; 115 Paul Scherrer Institut, Villigen, Switzerland; 116 Institute for Particle Physics ETH Zurich, Zurich, Switzerland; 117 Universität Zürich, Zurich, Switzerland; 118 National Central University, Chung-Li, Taiwan; 119 National Taiwan University (NTU), Taipei, Taiwan; 120 Department of Physics, Faculty of Science, Chulalongkorn University, Bangkok, Thailand; 121 Physics Department Science and Art Faculty, Cukurova University, Adana, Turkey; 122 Physics Department, Middle East Technical University, Ankara, Turkey; 123 Bogazici University, Istanbul, Turkey; 124 Istanbul Technical University, Istanbul, Turkey; 125 Institute for Scintillation Materials of National Academy of Science of Ukraine, Kharkov, Ukraine; 126 National Scientific Center, Kharkov Institute of Physics and Technology, Kharkov, Ukraine; 127 University of Bristol, Bristol, UK; 128 Rutherford Appleton Laboratory, Didcot, UK; 129 Imperial College, London, UK; 130 Brunel University, Uxbridge, UK; 131 Baylor University, Waco, USA; 132 Catholic University of America, Washington, DC, USA; 133 The University of Alabama, Tuscaloosa, USA; 134 Boston University, Boston, USA; 135 Brown University, Providence, USA; 136 University of California, Davis, Davis, USA; 137 University of California, Los Angeles, USA; 138 University of California, Riverside, Riverside, USA; 139 University of California, San Diego, La Jolla, USA; 140 Department of Physics, University of California, Santa Barbara, Santa Barbara, USA; 141 California Institute of Technology, Pasadena, USA; 142 Carnegie Mellon University, Pittsburgh, USA; 143 University of Colorado Boulder, Boulder, USA; 144 Cornell University, Ithaca, USA; 145 Fairfield University, Fairfield, USA; 146 Fermi National Accelerator Laboratory, Batavia, USA; 147 University of Florida, Gainesville, USA; 148 Florida International University, Miami, USA; 149 Florida State University, Tallahassee, USA; 150 Florida Institute of Technology, Melbourne, USA; 151 University of Illinois at Chicago (UIC), Chicago, USA; 152 The University of Iowa, Iowa City, USA; 153 Johns Hopkins University, Baltimore, USA; 154 The University of Kansas, Lawrence, USA; 155 Kansas State University, Manhattan, USA; 156 Lawrence Livermore National Laboratory, Livermore, USA; 157 University of Maryland, College Park, USA; 158 Massachusetts Institute of Technology, Cambridge, USA; 159 University of Minnesota, Minneapolis, USA; 160 University of Mississippi, Oxford, USA; 161 University of Nebraska-Lincoln, Lincoln, USA; 162 State University of New York at Buffalo, Buffalo, USA; 163 Northeastern University, Boston, USA; 164 Northwestern University, Evanston, USA; 165 University of Notre Dame, Notre Dame, USA; 166 The Ohio State University, Columbus, USA; 167 Princeton University, Princeton, USA; 168 University of Puerto Rico, Mayagüez, USA; 169 Purdue University, West Lafayette, USA; 170 Purdue University Northwest, Hammond, USA; 171 Rice University, Houston, USA; 172 University of Rochester, Rochester, USA; 173 Rutgers, The State University of New Jersey, Piscataway, USA; 174 University of Tennessee, Knoxville, USA; 175 Texas A&M University, College Station, USA; 176 Texas Tech University, Lubbock, USA; 177 Vanderbilt University, Nashville, USA; 178 University of Virginia, Charlottesville, USA; 179 Wayne State University, Detroit, USA; 180 University of Wisconsin-Madison, Madison, WI USA; 181 CERN, 1211 Geneva 23, Switzerland

## Abstract

Normalized double-differential cross sections for top quark pair ($\mathrm{t}\overline{\mathrm{t}}$) production are measured in pp collisions at a centre-of-mass energy of 8$\,\text {TeV}$ with the CMS experiment at the LHC. The analyzed data correspond to an integrated luminosity of 19.7$\,\text {fb}^{-1}$. The measurement is performed in the dilepton $\mathrm {e}^{\pm }\mu ^{\mp }$ final state. The $\mathrm{t}\overline{\mathrm{t}}$ cross section is determined as a function of various pairs of observables characterizing the kinematics of the top quark and $\mathrm{t}\overline{\mathrm{t}}$ system. The data are compared to calculations using perturbative quantum chromodynamics at next-to-leading and approximate next-to-next-to-leading orders. They are also compared to predictions of Monte Carlo event generators that complement fixed-order computations with parton showers, hadronization, and multiple-parton interactions. Overall agreement is observed with the predictions, which is improved when the latest global sets of proton parton distribution functions are used. The inclusion of the measured $\mathrm{t}\overline{\mathrm{t}}$ cross sections in a fit of parametrized parton distribution functions is shown to have significant impact on the gluon distribution.

## Introduction

Understanding the production and properties of the top quark, discovered in 1995 at the Fermilab Tevatron [1, 2], is fundamental in testing the standard model and searching for new phenomena. A large sample of proton–proton (pp) collision events containing a top quark pair ($\mathrm{t}\overline{\mathrm{t}}$) has been recorded at the CERN LHC, facilitating precise top quark measurements. In particular, precise measurements of the $\mathrm{t}\overline{\mathrm{t}}$ production cross section as a function of $\mathrm{t}\overline{\mathrm{t}}$ kinematic observables have become possible, which allow for the validation of the most-recent predictions of perturbative quantum chromodynamics (QCD). At the LHC, top quarks are predominantly produced via gluon–gluon fusion. Thus, using measurements of the production cross section in a global fit of the parton distribution functions (PDFs) can help to better determine the gluon distribution at large values of *x*, where *x* is the fraction of the proton momentum carried by a parton [3–5]. In this context, $\mathrm{t}\overline{\mathrm{t}}$ measurements are complementary to studies [6–8] that exploit inclusive jet production cross sections at the LHC.

Normalized differential cross sections for $\mathrm{t}\overline{\mathrm{t}}$ production have been measured previously in proton–antiproton collisions at the Tevatron at a centre-of-mass energy of 1.96$\,\text {TeV}$ [9, 10] and in pp collisions at the LHC at $\sqrt{s} = 7$ $\,\text {TeV}$  [11–14], 8$\,\text {TeV}$  [14–16], and 13$\,\text {TeV}$  [17]. This paper presents the measurement of the normalized double-differential $\mathrm{t}\overline{\mathrm{t}} + \mathrm {X}$ production cross section, where X is inclusive in the number of extra jets in the event but excludes $\mathrm{t}\overline{\mathrm{t}} +\mathrm{Z}/\mathrm {W}/\gamma$ production. The cross section is measured as a function of observables describing the kinematics of the top quark and $\mathrm{t}\overline{\mathrm{t}}$: the transverse momentum of the top quark, $p_{\mathrm {T}} (\mathrm{t})$, the rapidity of the top quark, $y(\mathrm{t})$, the transverse momentum, $p_{\mathrm {T}} (\mathrm{t}\overline{\mathrm{t}})$, the rapidity, $y(\mathrm{t}\overline{\mathrm{t}})$, and the invariant mass, $M(\mathrm{t}\overline{\mathrm{t}})$, of $\mathrm{t}\overline{\mathrm{t}}$, the pseudorapidity between the top quark and antiquark, $\varDelta \eta (\mathrm{t},\overline{\mathrm{t}})$, and the angle between the top quark and antiquark in the transverse plane, $\varDelta \phi (\mathrm{t},\overline{\mathrm{t}})$. In total, the double-differential $\mathrm{t}\overline{\mathrm{t}}$ cross section is measured as a function of six different pairs of kinematic variables.

These measurements provide a sensitive test of the standard model by probing the details of the $\mathrm{t}\overline{\mathrm{t}}$ production dynamics. The double-differential measurement is expected to impose stronger constraints on the gluon distribution than single-differential measurements owing to the improved resolution of the momentum fractions carried by the two incoming partons.

The analysis uses the data recorded at $\sqrt{s}=8$ $\,\text {TeV}$ by the CMS experiment in 2012, corresponding to an integrated luminosity of $19.7 \pm 0.5{\,\text {fb}^{-1}}$. The measurement is performed using the $\mathrm {e}^{\pm }\mu ^{\mp }$ decay mode ($\mathrm {e}\mu$) of $\mathrm{t}\overline{\mathrm{t}}$, requiring two oppositely charged leptons and at least two jets. The analysis largely follows the procedures of the single-differential $\mathrm{t}\overline{\mathrm{t}}$ cross section measurement [15]. The restriction to the $\mathrm {e}\mu$ channel provides a pure $\mathrm{t}\overline{\mathrm{t}}$ event sample because of the negligible contamination from $\mathrm{Z}/\gamma ^{*}$ processes with same-flavour leptons in the final state.

The measurements are defined at parton level and thus are corrected for the effects of hadronization and detector resolutions and inefficiencies. A regularized unfolding process is performed simultaneously in bins of the two variables in which the cross sections are measured. The normalized differential $\mathrm{t}\overline{\mathrm{t}}$ cross section is determined by dividing by the measured total inclusive $\mathrm{t}\overline{\mathrm{t}}$ production cross section, where the latter is evaluated by integrating over all bins in the two observables. The parton level results are compared to different theoretical predictions from leading-order (LO) and next-to-leading-order (NLO) Monte Carlo (MC) event generators, as well as with fixed-order NLO [18] and approximate next-to-next-to-leading-order (NNLO) [19] calculations using several different PDF sets. Parametrized PDFs are fitted to the data in a procedure that is referred to as the PDF fit.

The structure of the paper is as follows: in Sect. 2 a brief description of the CMS detector is given. Details of the event simulation are provided in Sect. 3. The event selection, kinematic reconstruction, and comparisons between data and simulation are provided in Sect. 4. The two-dimensional unfolding procedure is detailed in Sect. 5; the method to determine the double-differential cross sections is presented in Sect. 6, and the assessment of the systematic uncertainties is described in Sect. 7. The results of the measurement are discussed and compared to theoretical predictions in Sect. 8. Section 9 presents the PDF fit. Finally, Sect. 10 provides a summary.

## The CMS detector

The central feature of the CMS apparatus is a superconducting solenoid of 13$\text { m}$ length and 6$\text { m}$ inner diameter, which provides an axial magnetic field of 3.8$\text { T}$. Within the field volume are a silicon pixel and strip tracker, a lead tungstate crystal electromagnetic calorimeter (ECAL), and a brass and scintillator hadron calorimeter (HCAL), each composed of a barrel and two endcap sections. Extensive forward calorimetry complements the coverage provided by the barrel and endcap sections up to $|\eta |<5.2$. Charged particle trajectories are measured by the inner tracking system, covering a range of $|\eta |<2.5$. The ECAL and HCAL surround the tracking volume, providing high-resolution energy and direction measurements of electrons, photons, and hadronic jets up to $|\eta |<3$. Muons are measured in gas-ionization detectors embedded in the steel flux-return yoke outside the solenoid covering the region $|\eta |<2.4$. The detector is nearly hermetic, allowing momentum balance measurements in the plane transverse to the beam directions. A more detailed description of the CMS detector, together with a definition of the coordinate system and the relevant kinematic variables, can be found in Ref. [20].

## Signal and background modelling

The $\mathrm{t}\overline{\mathrm{t}}$ signal process is simulated using the matrix element event generator MadGraph (version 5.1.5.11) [21], together with the MadSpin  [22] package for the modelling of spin correlations. The pythia 6 program (version 6.426) [23] is used to model parton showering and hadronization. In the signal simulation, the mass of the top quark, $m_{\mathrm{t}}$, is fixed to 172.5$\,\text {GeV}$. The proton structure is described by the CTEQ6L1 PDF set [24]. The same programs are used to model dependencies on the renormalization and factorization scales, $\mu _\mathrm {r}$ and $\mu _\mathrm {f}$, respectively, the matching threshold between jets produced at the matrix-element level and via parton showering, and $m_{\mathrm{t}}$.

The cross sections obtained in this paper are also compared to theoretical calculations obtained with the NLO event generators powheg (version 1.0 r1380) [25–27], interfaced with pythia 6 or Herwig6 (version 6.520) [28] for the subsequent parton showering and hadronization, and mc@nlo (version 3.41) [29], interfaced with herwig. Both pythia 6 and herwig 6 include a modelling of multiple-parton interactions and the underlying event. The pythia 6 Z2* tune [30] is used to characterize the underlying event in both the $\mathrm{t}\overline{\mathrm{t}}$ and the background simulations. The herwig 6 AUET2 tune [31] is used to model the underlying event in the powheg +herwig 6 simulation, while the default tune is used in the mc@nlo +herwig 6 simulation. The PDF sets CT10 [32] and CTEQ6M [24] are used for powheg and mc@nlo, respectively. Additional simulated event samples generated with powheg and interfaced with pythia 6 or herwig 6 are used to assess the systematic uncertainties related to the modelling of the hard-scattering process and hadronization, respectively, as described in Sect. 7.

The production of W and Z/$\gamma ^{*}$ bosons with additional jets, respectively referred to as W+jets and Z/$\gamma ^{*}$+jets in the following, and $\mathrm{t}\overline{\mathrm{t}} +\mathrm{Z}/\mathrm {W}/\gamma$ backgrounds are simulated using MadGraph, while $\mathrm {W}$ boson plus associated single top quark production ($\mathrm{t} \mathrm {W}$) is simulated using powheg. The showering and hadronization is modelled with pythia 6 for these processes. Diboson (WW, WZ, and ZZ) samples, as well as QCD multijet backgrounds, are produced with pythia 6. All of the background simulations are normalized to the fixed-order theoretical predictions as described in Ref. [15]. The CMS detector response is simulated using Geant4 (version 9.4) [33].

## Event selection

The event selection follows closely the one reported in Ref. [15]. The top quark decays almost exclusively into a W boson and a bottom quark, and only events in which the two W bosons decay into exactly one electron and one muon and corresponding neutrinos are considered. Events are triggered by requiring one electron and one muon of opposite charge, one of which is required to have $p_{\mathrm {T}} > 17$ $\,\text {GeV}$ and the other $p_{\mathrm {T}} > 8$ $\,\text {GeV}$.

Events are reconstructed using a particle-flow (PF) technique [34, 35], which combines signals from all subdetectors to enhance the reconstruction and identification of the individual particles observed in pp collisions. An interaction vertex [36] is required within 24$\text { cm}$ of the detector centre along the beam line direction, and within 2$\text { cm}$ of the beam line in the transverse plane. Among all such vertices, the primary vertex of an event is identified as the one with the largest value of the sum of the $p_{\mathrm {T}} ^2$ of the associated tracks. Charged hadrons from pileup events, i.e. those originating from additional pp interactions within the same or nearby bunch crossing, are subtracted on an event-by-event basis. Subsequently, the remaining neutral-hadron component from pileup is accounted for through jet energy corrections [37].

Electron candidates are reconstructed from a combination of the track momentum at the primary vertex, the corresponding energy deposition in the ECAL, and the energy sum of all bremsstrahlung photons associated with the track [38]. Muon candidates are reconstructed using the track information from the silicon tracker and the muon system. An event is required to contain at least two oppositely charged leptons, one electron and one muon, each with $p_{\mathrm {T}} > 20\,\text {GeV}$ and $|\eta | < 2.4$. Only the electron and the muon with the highest $p_{\mathrm {T}}$ are considered for the analysis. The invariant mass of the selected electron and muon must be larger than 20$\,\text {GeV}$ to suppress events from decays of heavy-flavour resonances. The leptons are required to be isolated with $I_\text {rel}\le 0.15$ inside a cone in $\eta$-$\phi$ space of $\varDelta R = \sqrt{(\varDelta \eta )^{2} + (\varDelta \phi )^{2}} = 0.3$ around the lepton track, where $\varDelta \eta$ and $\varDelta \phi$ are the differences in pseudorapidity and azimuthal angle (in radians), respectively, between the directions of the lepton and any other particle. The parameter $I_\text {rel}$ is the relative isolation parameter defined as the sum of transverse energy deposits inside the cone from charged and neutral hadrons, and photons, relative to the lepton $p_{\mathrm {T}}$, corrected for pileup effects. The efficiencies of the lepton isolation were determined in *Z* boson data samples using the “tag-and-probe” method of Ref. [39], and are found to be well described by the simulation for both electrons and muons. The overall difference between data and simulation is estimated to be ${<} 2\%$ for electrons, and ${<}1\%$ for muons. The simulation is adjusted for this by using correction factors parametrized as a function of the lepton $p_{\mathrm {T}}$ and $\eta$ and applied to simulated events, separately for electrons and muons.

Jets are reconstructed by clustering the PF candidates using the anti-$k_{\mathrm {T}}$ clustering algorithm [40, 41] with a distance parameter $R = 0.5$. Electrons and muons passing less-stringent selections on lepton kinematic quantities and isolation, relative to those specified above, are identified but excluded from clustering. A jet is selected if it has $p_{\mathrm {T}} > 30\,\text {GeV}$ and $|\eta | < 2.4$. Jets originating from the hadronization of b quarks (b jets) are identified using an algorithm [42] that provides a b tagging discriminant by combining secondary vertices and track-based lifetime information. This provides a b tagging efficiency of ${\approx }80$–85% for b jets and a mistagging efficiency of ${\approx }10\%$ for jets originating from gluons, as well as u, d, or s quarks, and ${\approx }30$–40% for jets originating from c quarks [42]. Events are selected if they contain at least two jets, and at least one of these jets is b-tagged. These requirements are chosen to reduce the background contribution while keeping a large fraction of the $\mathrm{t}\overline{\mathrm{t}}$ signal. The $\mathrm{b}$ tagging efficiency is adjusted in the simulation with the correction factors parametrized as a function of the jet $p_{\mathrm {T}}$ and $\eta$.

The missing transverse momentum vector is defined as the projection on the plane perpendicular to the beams of the negative vector sum of the momenta of all PF particles in an event [43]. Its magnitude is referred to as $p_{\mathrm {T}} ^\text {miss}$. To mitigate the pileup effects on the $p_{\mathrm {T}} ^\text {miss}$ resolution, a multivariate correction is used where the measured momentum is separated into components that originate from the primary and from other interaction vertices [44]. No selection requirement on $p_{\mathrm {T}} ^\text {miss}$ is applied.

The $\mathrm{t}\overline{\mathrm{t}}$ kinematic properties are determined from the four-momenta of the decay products using the same kinematic reconstruction method [45, 46] as that of the single-differential $\mathrm{t}\overline{\mathrm{t}}$ measurement [15]. The six unknown quantities are the three-momenta of the two neutrinos, which are reconstructed by imposing the following six kinematic constraints: $p_{\mathrm {T}}$ conservation in the event and the masses of the W bosons, top quark, and top antiquark. The top quark and antiquark are required to have a mass of 172.5$\,\text {GeV}$. It is assumed that the $p_{\mathrm {T}} ^\text {miss}$ in the event results from the two neutrinos in the top quark and antiquark decay chains. To resolve the ambiguity due to multiple algebraic solutions of the equations for the neutrino momenta, the solution with the smallest invariant mass of the $\mathrm{t}\overline{\mathrm{t}}$ system is taken. The reconstruction is performed 100 times, each time randomly smearing the measured energies and directions of the reconstructed leptons and jets within their resolution. This smearing recovers events that yielded no solution because of measurement fluctuations. The three-momenta of the two neutrinos are determined as a weighted average over all the smeared solutions. For each solution, the weight is calculated based on the expected invariant mass spectrum of a lepton and a bottom jet as the product of two weights for the top quark and antiquark decay chains. All possible lepton–jet combinations in the event are considered. Combinations are ranked based on the presence of b-tagged jets in the assignments, i.e. a combination with both leptons assigned to b-tagged jets is preferred over those with one or no b-tagged jet. Among assignments with equal number of b-tagged jets, the one with the highest average weight is chosen. Events with no solution after smearing are discarded. The method yields an average reconstruction efficiency of ${\approx }95\%$, which is determined in simulation as the fraction of selected signal events (which include only direct $\mathrm{t}\overline{\mathrm{t}}$ decays via the $\mathrm {e}^{\pm }\mu ^{\mp }$ channel, i.e. excluding cascade decays via $\tau$ leptons) passing the kinematic reconstruction. The overall difference in this efficiency between data and simulation is estimated to be ${\approx }1\%$, and a corresponding correction factor is applied to the simulation [47]. A more detailed description of the kinematic reconstruction procedure can be found in Ref. [47].

In total, 38, 569 events are selected in the data. The signal contribution to the event sample is 79.2%, as estimated from the simulation. The remaining fraction of events is dominated by $\mathrm{t}\overline{\mathrm{t}}$ decays other than via the $\mathrm {e}^{\pm }\mu ^{\mp }$ channel (14.2%). Other sources of background are single top quark production (3.6%), Z/$\gamma ^{*}$+jets events (1.4%), associated $\mathrm{t}\overline{\mathrm{t}} +\mathrm{Z}/\mathrm {W}/\gamma$ production (1.1%), and a negligible (${<} 0.5\%$) fraction of diboson, W+jets, and QCD multijet events.

**Fig. 1:**
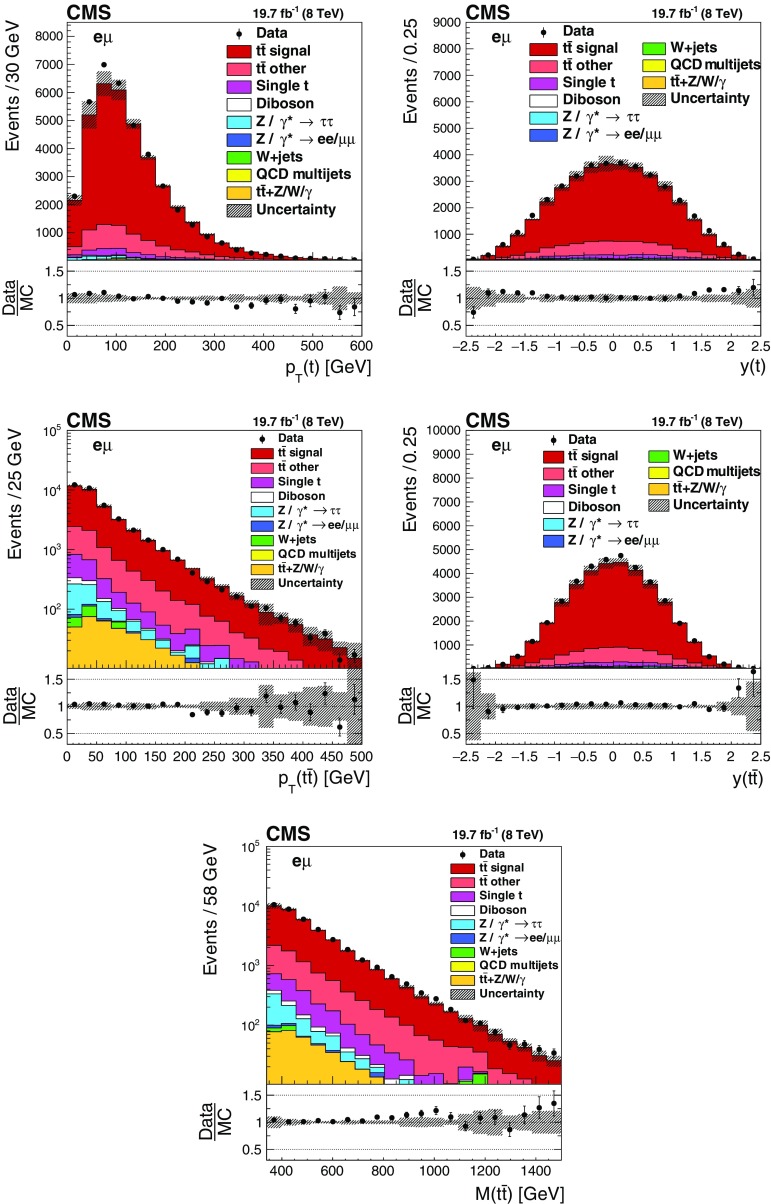
Distributions of $p_{\mathrm {T}} (\mathrm{t})$ (*upper left*), $y(\mathrm{t})$ (*upper right*), $p_{\mathrm {T}} (\mathrm{t}\overline{\mathrm{t}})$ (*middle left*), $y(\mathrm{t}\overline{\mathrm{t}})$ (*middle right*), and $M(\mathrm{t}\overline{\mathrm{t}})$ (*lower*) in selected events after the kinematic reconstruction. The experimental data with the *vertical bars* corresponding to their statistical uncertainties are plotted together with distributions of simulated signal and different background processes. The hatched regions correspond to the shape uncertainties in the signal and backgrounds (cf. Sect. 7). The *lower panel in each plot* shows the ratio of the observed data event yields to those expected in the simulation

Figure 1 shows the distributions of the reconstructed top quark and $\mathrm{t}\overline{\mathrm{t}}$ kinematic variables. In general, the data are reasonably well described by the simulation, however some trends are visible. In particular, the simulation shows a harder $p_{\mathrm {T}} (\mathrm{t})$ spectrum than the data, as observed in previous measurements [12–17]. The $y(\mathrm{t}\overline{\mathrm{t}})$ distribution is found to be less central in the simulation than in the data, while an opposite behavior is observed in the $y(\mathrm{t})$ distribution. The $M(\mathrm{t}\overline{\mathrm{t}})$ and $p_{\mathrm {T}} (\mathrm{t}\overline{\mathrm{t}})$ distributions are overall well described by the simulation.

## Signal extraction and unfolding

The number of signal events, $N^\text {sig}_i$, is extracted from the data in the *i*th bin of the reconstructed observables using

1 $$\begin{aligned} N^\mathrm{{sig}}_i = N^\mathrm{{sel}}_i - N^\mathrm{{bkg}}_i, \quad 1 \le i \le n, \end{aligned}$$

where *n* denotes the total number of bins, $N^\mathrm{{sel}}_i$ is the number of selected events in the *i*th bin, and $N^\mathrm{{bkg}}_i$ corresponds to the expected number of background events in this bin, except for $\mathrm{t}\overline{\mathrm{t}}$ final states other than the signal. The latter are dominated by events in which one or both of the intermediate W bosons decay into $\tau$ leptons with subsequent decay into an electron or muon. Since these events arise from the same $\mathrm{t}\overline{\mathrm{t}}$ production process as the signal, the normalisation of this background is fixed to that of the signal. The expected signal fraction is defined as the ratio of the number of selected $\mathrm{t}\overline{\mathrm{t}}$ signal events to the total number of selected $\mathrm{t}\overline{\mathrm{t}}$ events (i.e. the signal and all other $\mathrm{t}\overline{\mathrm{t}}$ events) in simulation. This procedure avoids the dependence on the total inclusive $\mathrm{t}\overline{\mathrm{t}}$ cross section used in the normalization of the simulated signal sample.

The signal yields $N^\mathrm{{sig}}_i$, determined in each *i*th bin of the reconstructed kinematic variables, may contain entries that were originally produced in other bins and have migrated because of the imperfect resolutions. This effect can be described as

2 $$\begin{aligned} {M}^\mathrm{{sig}}_i = \sum _{j = 1}^{m} A_{ij}{M}_{j}^\mathrm{{unf}}, \quad 1 \le i \le n, \end{aligned}$$

where *m* denotes the total number of bins in the true distribution, and ${M}_{j}^\mathrm{{unf}}$ is the number of events in the *j*th bin of the true distribution from data. The quantity ${M}^\mathrm{{sig}}_i$ is the expected number of events at detector level in the *i*th bin, and $A_{ij}$ is a matrix of probabilities describing the migrations from the *j*th bin of the true distribution to the *i*th bin of the detector-level distribution, including acceptance and detector efficiencies. In this analysis, the migration matrix $A_{ij}$ is defined such that the true level corresponds to the full phase space (with no kinematic restrictions) for $\mathrm{t}\overline{\mathrm{t}}$ production at parton level. At the detector level a binning is chosen in the same kinematic ranges as at the true level, but with the total number of bins typically a few times larger. The kinematic ranges of all variables are chosen such that the fraction of events that migrate into the regions outside the measured range is very small. It was checked that the inclusion of overflow bins outside the kinematic ranges does not significantly alter the unfolded results. The migration matrix $A_{ij}$ is taken from the signal simulation. The observed event counts $N^\mathrm{{sig}}_i$ may be different from ${M}_i^\mathrm{{sig}}$ owing to statistical fluctuations.

The estimated value of ${M}_{j}^\mathrm{{unf}}$, designated as $\hat{M_{j}}^\mathrm{{unf}}$, is found using the TUnfold algorithm [48]. The unfolding of multidimensional distributions is performed by mapping the multidimensional arrays to one-dimensional arrays internally [48]. The unfolding is realized by a $\chi ^2$ minimization and includes an additional $\chi ^2$ term representing the Tikhonov regularization [49]. The regularization reduces the effect of the statistical fluctuations present in $N^\mathrm{{sig}}_i$ on the high-frequency content of $\hat{M_{j}}^\mathrm{{unf}}$. The regularization strength is chosen such that the global correlation coefficient is minimal [50]. For the measurements presented here, this choice results in a small contribution from the regularization term to the total $\chi ^2$, on the order of 1%. A more detailed description of the unfolding procedure can be found in Ref. [47].

## Cross section determination

The normalized double-differential cross sections of $\mathrm{t}\overline{\mathrm{t}}$ production are measured in the full $\mathrm{t}\overline{\mathrm{t}}$ kinematic phase space at parton level. The number of unfolded signal events $\hat{M}^\mathrm{{unf}}_{ij}$ in bin *i* of variable *x* and bin *j* of variable *y* is used to define the normalized double-differential cross sections of the $\mathrm{t}\overline{\mathrm{t}}$ production process,

3 $$\begin{aligned} \left( \frac{1}{\sigma } \frac{\mathrm{d}^{2}\sigma }{\mathrm{d}x\,\mathrm{d}y}\right) _{ij} = \frac{1}{\sigma } \, \frac{1}{\varDelta x_{i}} \,\frac{1}{\varDelta y_{j}} \, \frac{\hat{M}^\mathrm{{unf}}_{ij}}{\mathcal {B} \, \mathcal {L}}, \end{aligned}$$

where $\sigma$ is the total cross section, which is evaluated by integrating $(\mathrm{d}^{2}\sigma /\mathrm{d}x\,\mathrm{d}y)_{ij}$ over all bins. The branching fraction of $\mathrm{t}\overline{\mathrm{t}}$ into $\mathrm {e}\mu$ final state is taken to be $\mathcal {B} = 2.3\%$ [51], and $\mathcal {L}$ is the integrated luminosity of the data sample. The bin widths of the *x* and *y* variables are denoted by $\varDelta x_{i}$ and $\varDelta y_{j}$, respectively. The bin widths are chosen based on the resolution, such that the purity and the stability of each bin is generally above 30%. For a given bin, the purity is defined as the fraction of events in the $\mathrm{t}\overline{\mathrm{t}}$ signal simulation that are generated and reconstructed in the same bin with respect to the total number of events reconstructed in that bin. To evaluate the stability, the number of events in the $\mathrm{t}\overline{\mathrm{t}}$ signal simulation that are generated and reconstructed in a given bin are divided by the total number of reconstructed events generated in the bin.

## Systematic uncertainties

The measurement is affected by systematic uncertainties that originate from detector effects and from the modelling of the processes. Each source of systematic uncertainty is assessed individually by changing in the simulation the corresponding efficiency, resolution, or scale by its uncertainty, using a prescription similar to the one followed in Ref. [15]. For each change made, the cross section determination is repeated, and the difference with respect to the nominal result in each bin is taken as the systematic uncertainty.

To account for the pileup uncertainty, the value of the total $\mathrm {p}\mathrm {p}$ inelastic cross section, which is used to estimate the mean number of additional pp interactions, is varied by ${\pm }5\%$ [52]. The data-to-simulation correction factors for $\mathrm{b}$ tagging and mistagging efficiencies are varied within their uncertainties [42] as a function of the $p_{\mathrm {T}}$ and $|\eta |$ of the jet, following the procedure described in Ref. [15]. The data-to-simulation correction factors for the trigger efficiency, determined relatively to the triggers based on $p_{\mathrm {T}} ^\text {miss}$, are varied within their uncertainty of 1%. The systematic uncertainty related to the kinematic reconstruction of top quarks is assessed by varying the MC correction factor by its estimated uncertainty of ${\pm }1$% [47]. For the uncertainties related to the jet energy scale, the jet energy is varied in the simulation within its uncertainty [53]. The uncertainty owing to the limited knowledge of the jet energy resolution is determined by changing the latter in the simulation by ${\pm }1$ standard deviation in different $\eta$ regions [54]. The normalizations of the background processes are varied by 30% to account for the corresponding uncertainty. The uncertainty in the integrated luminosity of 2.6% [55] is propagated to the measured cross sections.

The impact of theoretical assumptions on the measurement is determined by repeating the analysis replacing the standard MadGraph $\mathrm{t}\overline{\mathrm{t}}$ simulation with simulated samples in which specific parameters or assumptions are altered. The PDF systematic uncertainty is estimated by reweighting the MadGraph $\mathrm{t}\overline{\mathrm{t}}$ signal sample according to the uncertainties in the CT10 PDF set, evaluated at 90% confidence level (CL) [32], and then rescaled to 68% CL. To estimate the uncertainty related to the choice of the tree-level multijet scattering model used in MadGraph, the results are recalculated using an alternative prescription for interfacing NLO calculations with parton showering as implemented in powheg. For $\mu _\mathrm {r}$ and $\mu _\mathrm {f}$, two samples are used with the scales being simultaneously increased or decreased by a factor of two relative to their common nominal value $\mu _\mathrm {r} = \mu _\mathrm {f} = \sqrt{\smash [b]{m^2_{\mathrm{t}} + \Sigma p_{\mathrm {T}} ^2}}$, where the sum is over all additional final-state partons in the matrix element. The effect of additional jet production is studied by varying in MadGraph the matching threshold between jets produced at the matrix-element level and via parton showering. The uncertainty in the effect of the initial- and final-state radiation on the signal efficiency is covered by the uncertainty in $\mu _\mathrm {r}$ and $\mu _\mathrm {f}$, as well as in the matching threshold. The samples generated with powheg +herwig 6 and powheg +pythia 6 are used to estimate the uncertainty related to the choice of the showering and hadronization model. The effect due to uncertainties in $m_{\mathrm{t}}$ is estimated using simulations with altered top quark masses. The cross section differences observed for an $m_{\mathrm{t}}$ variation of 1$\,\text {GeV}$ around the central value of 172.5$\,\text {GeV}$ used in the simulation is quoted as the uncertainty.

The total systematic uncertainty is estimated by adding all the contributions described above in quadrature, separately for positive and negative cross section variations. If a systematic uncertainty results in two cross section variations of the same sign, the largest one is taken, while the opposite variation is set to zero.

## Results

Normalized differential $\mathrm{t}\overline{\mathrm{t}}$ cross sections are measured as a function of pairs of variables representing the kinematics of the top quark (only the top quark is taken and not the top antiquark, thus avoiding any double counting of events), and $\mathrm{t}\overline{\mathrm{t}}$ system, defined in Sect. 1: $[p_{\mathrm {T}} (\mathrm{t}), y(\mathrm{t}) ]$, $[y(\mathrm{t}), M(\mathrm{t}\overline{\mathrm{t}}) ]$, $[y(\mathrm{t}\overline{\mathrm{t}}), M(\mathrm{t}\overline{\mathrm{t}}) ]$, $[\varDelta \eta (\mathrm{t},\overline{\mathrm{t}}), M(\mathrm{t}\overline{\mathrm{t}}) ]$, $[p_{\mathrm {T}} (\mathrm{t}\overline{\mathrm{t}}), M(\mathrm{t}\overline{\mathrm{t}}) ]$, and $[\varDelta \phi (\mathrm{t},\overline{\mathrm{t}}), M(\mathrm{t}\overline{\mathrm{t}}) ]$. These pairs are chosen in order to obtain representative combinations that are sensitive to different aspects of the $\mathrm{t}\overline{\mathrm{t}}$ production dynamics, as will be discussed in the following.

**Fig. 2:**
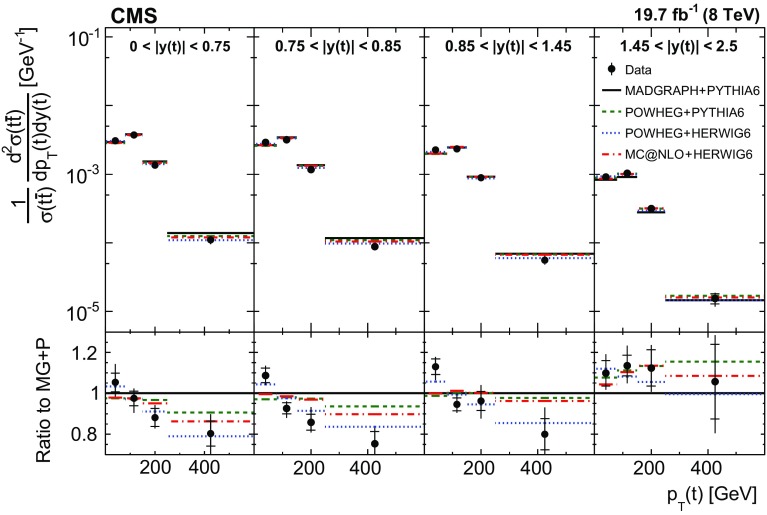
Comparison of the measured normalized $\mathrm{t}\overline{\mathrm{t}}$ double-differential cross section as a function of $p_{\mathrm {T}} (\mathrm{t})$ in different $|y(\mathrm{t}) |$ ranges to MC predictions calculated using MadGraph +pythia 6, powheg +pythia 6, powheg +herwig 6, and mc@nlo +herwig 6. The *inner vertical bars on the data points* represent the statistical uncertainties and the *full bars* include also the systematic uncertainties added in quadrature. In the *bottom panel*, the ratios of the data and other simulations to the MadGraph +pythia 6 (MG+P) predictions are shown

**Fig. 3:**
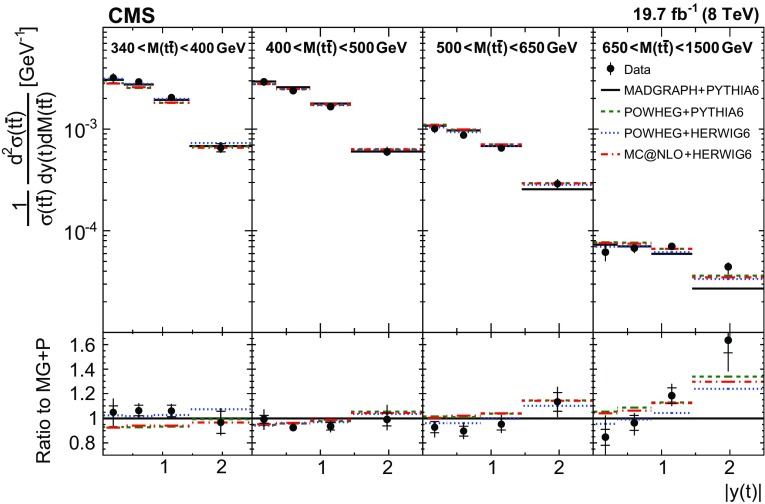
Comparison of the measured normalized $\mathrm{t}\overline{\mathrm{t}}$ double-differential cross section as a function of $|y(\mathrm{t}) |$ in different $M(\mathrm{t}\overline{\mathrm{t}})$ ranges to MC predictions. Details can be found in the caption of Fig. 2

**Fig. 4:**
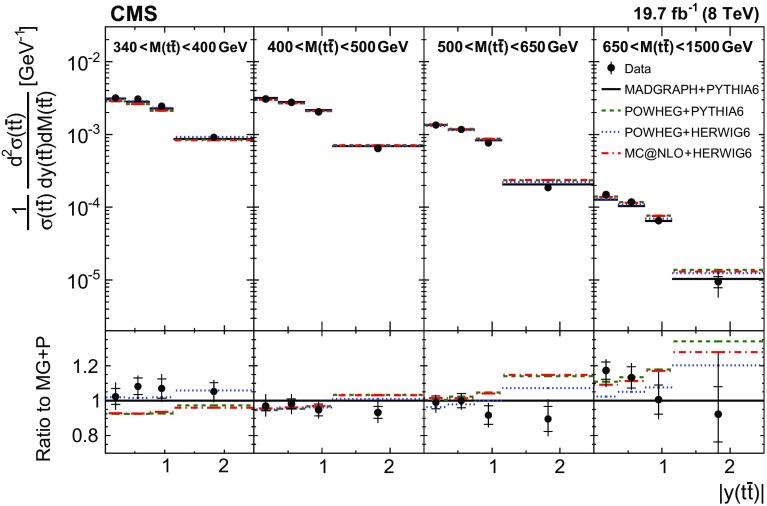
Comparison of the measured normalized $\mathrm{t}\overline{\mathrm{t}}$ double-differential cross section as a function of $|y(\mathrm{t}\overline{\mathrm{t}}) |$ in different of $M(\mathrm{t}\overline{\mathrm{t}})$ ranges to MC predictions. Details can be found in the caption of Fig. 2

**Fig. 5:**
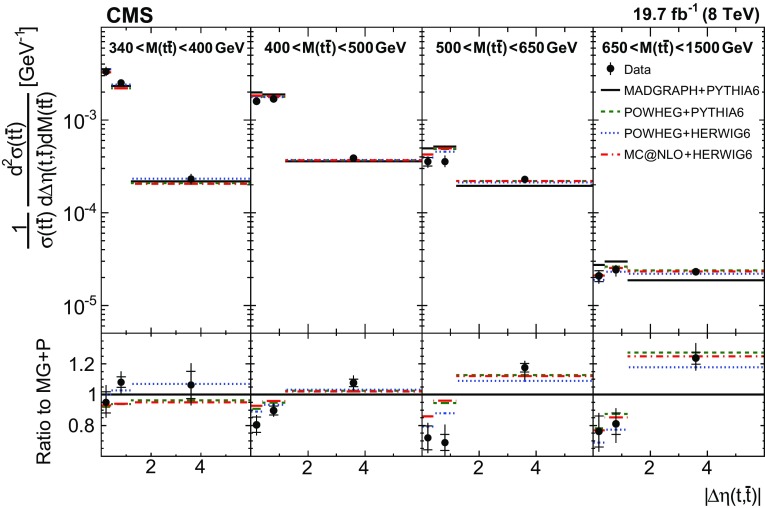
Comparison of the measured normalized $\mathrm{t}\overline{\mathrm{t}}$ double-differential cross section as a function of $\varDelta \eta (\mathrm{t},\overline{\mathrm{t}})$ in different $M(\mathrm{t}\overline{\mathrm{t}})$ ranges to MC predictions. Details can be found in the caption of Fig. 2

**Fig. 6:**
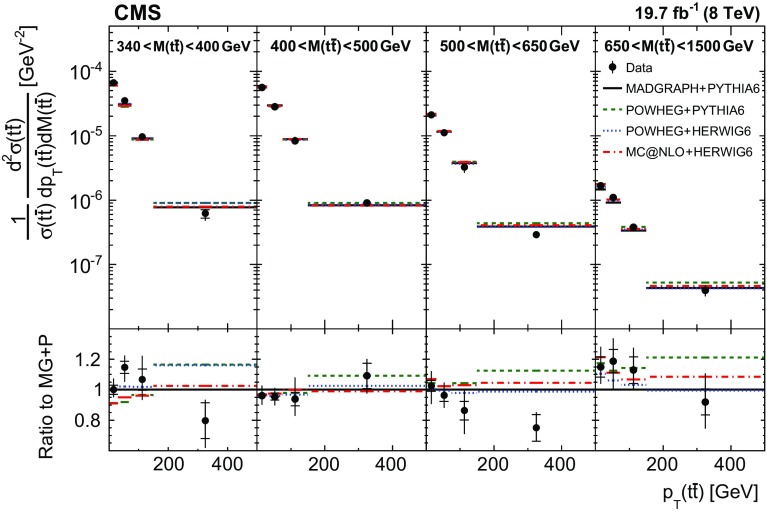
Comparison of the measured normalized $\mathrm{t}\overline{\mathrm{t}}$ double-differential cross section as a function of $p_{\mathrm {T}} (\mathrm{t}\overline{\mathrm{t}})$ in different $M(\mathrm{t}\overline{\mathrm{t}})$ ranges to MC predictions. Details can be found in the caption of Fig. 2

**Fig. 7:**
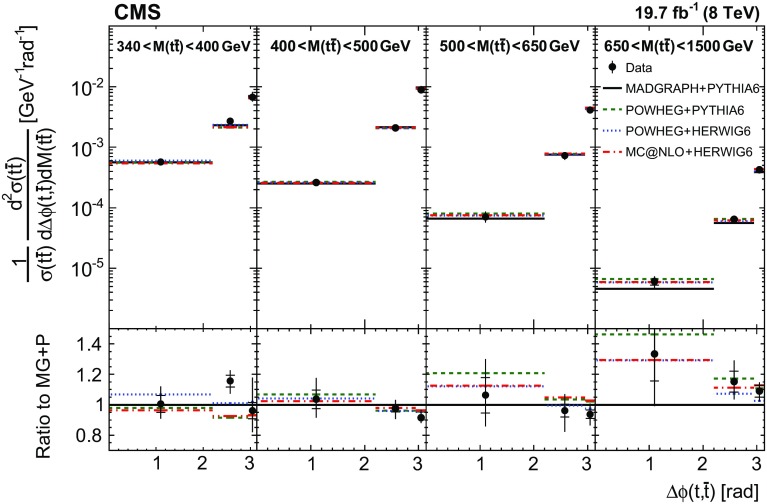
Comparison of the measured normalized $\mathrm{t}\overline{\mathrm{t}}$ double-differential cross section as a function of $\varDelta \phi (\mathrm{t},\overline{\mathrm{t}})$ in different $M(\mathrm{t}\overline{\mathrm{t}})$ ranges to MC predictions. Details can be found in the caption of Fig. 2

**Fig. 8:**
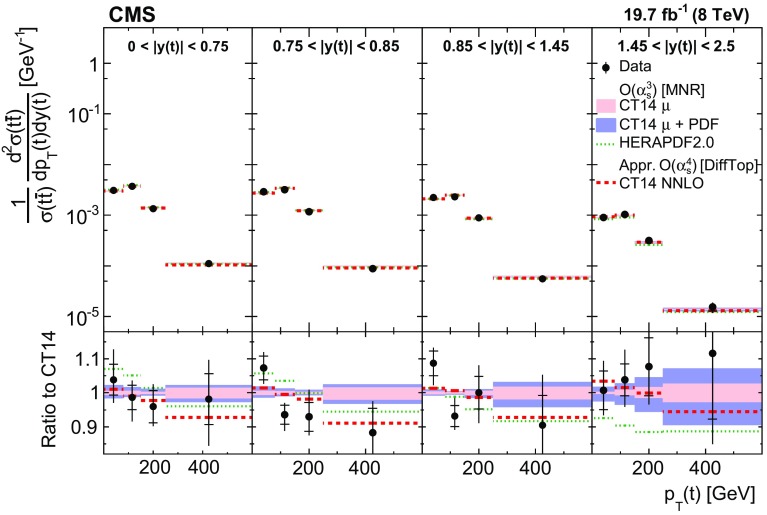
Comparison of the measured normalized $\mathrm{t}\overline{\mathrm{t}}$ double-differential cross section as a function of $p_{\mathrm {T}} (\mathrm{t})$ in different $|y(\mathrm{t}) |$ ranges to NLO $O(\alpha _s^3)$ (MNR) predictions calculated with CT14 and HERAPDF2.0, and approximate NNLO $O(\alpha _s^4)$ (DiffTop) prediction calculated with CT14. The *inner vertical bars on the data points* represent the statistical uncertainties and the *full bars* include also the systematic uncertainties added in quadrature. The *light band* shows the scale uncertainties ($\mu$) for the NLO predictions using CT14, while the *dark band* includes also the PDF uncertainties added in quadrature ($\mu + \mathrm {PDF}$). The *dotted line* shows the NLO predictions calculated with HERAPDF2.0. The *dashed line* shows the approximate NNLO predictions calculated with CT14. In the *bottom panel*, the ratios of the data and other calculations to the NLO prediction using CT14 are shown

**Fig. 9:**
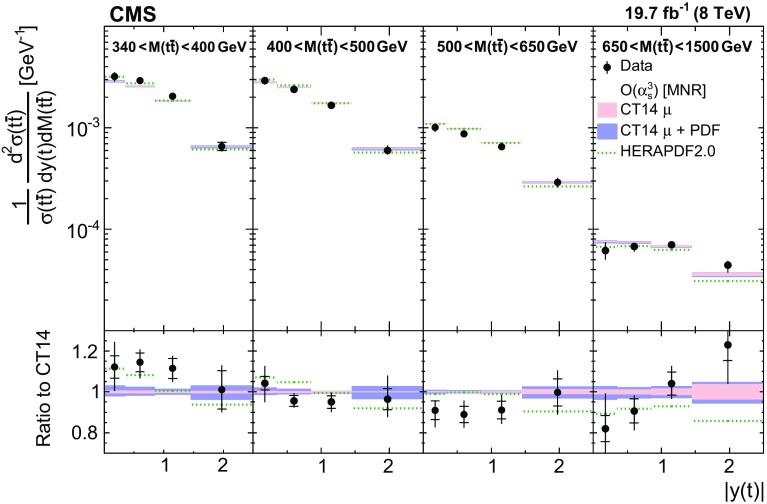
Comparison of the measured normalized $\mathrm{t}\overline{\mathrm{t}}$ double-differential cross section as a function of $|y(\mathrm{t}) |$ in different $M(\mathrm{t}\overline{\mathrm{t}})$ ranges to NLO $O(\alpha _s^3)$ predictions. Details can be found in the caption of Fig. 8. Approximate NNLO $O(\alpha _s^4)$ predictions are not available for this cross section

**Fig. 10:**
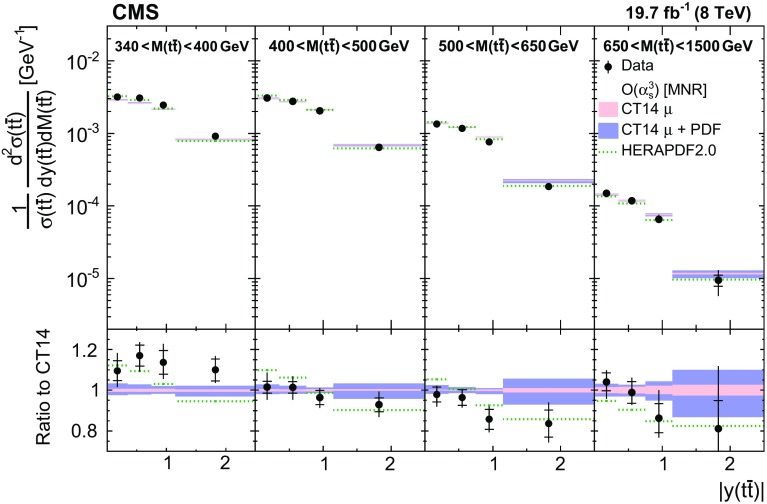
Comparison of the measured normalized $\mathrm{t}\overline{\mathrm{t}}$ double-differential cross section as a function of $|y(\mathrm{t}\overline{\mathrm{t}}) |$ in different $M(\mathrm{t}\overline{\mathrm{t}})$ ranges to NLO $O(\alpha _s^3)$ predictions. Details can be found in the caption of Fig. 8. Approximate NNLO $O(\alpha _s^4)$ predictions are not available for this cross section

**Fig. 11:**
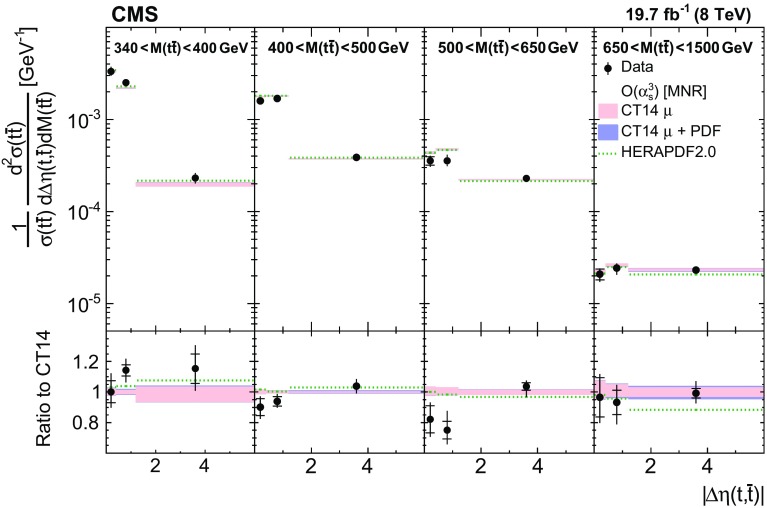
Comparison of the measured normalized $\mathrm{t}\overline{\mathrm{t}}$ double-differential cross section as a function of $\varDelta \eta (\mathrm{t},\overline{\mathrm{t}})$ in different $M(\mathrm{t}\overline{\mathrm{t}})$ ranges to NLO $O(\alpha _s^3)$ predictions. Details can be found in the caption of Fig. 8. Approximate NNLO $O(\alpha _s^4)$ predictions are not available for this cross section

**Fig. 12:**
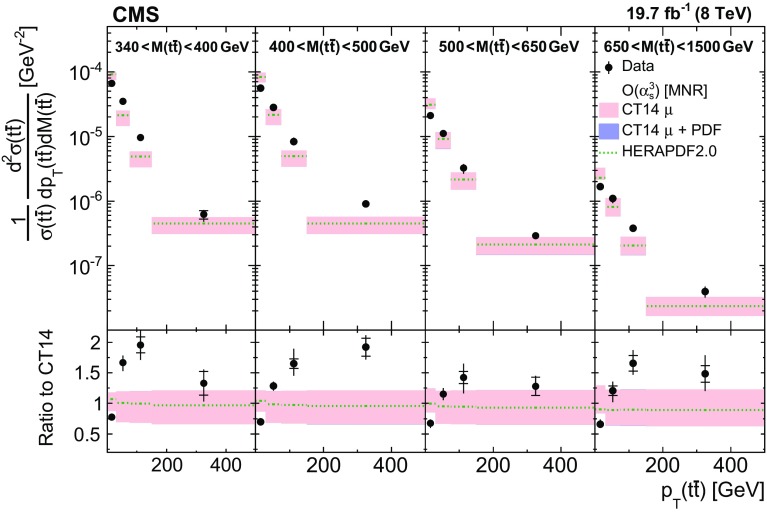
Comparison of the measured normalized $\mathrm{t}\overline{\mathrm{t}}$ double-differential cross section as a function of $p_{\mathrm {T}} (\mathrm{t}\overline{\mathrm{t}})$ in different $M(\mathrm{t}\overline{\mathrm{t}})$ ranges to NLO $O(\alpha _s^3)$ predictions. Details can be found in the caption of Fig. 8. Approximate NNLO $O(\alpha _s^4)$ predictions are not available for this cross section

**Fig. 13:**
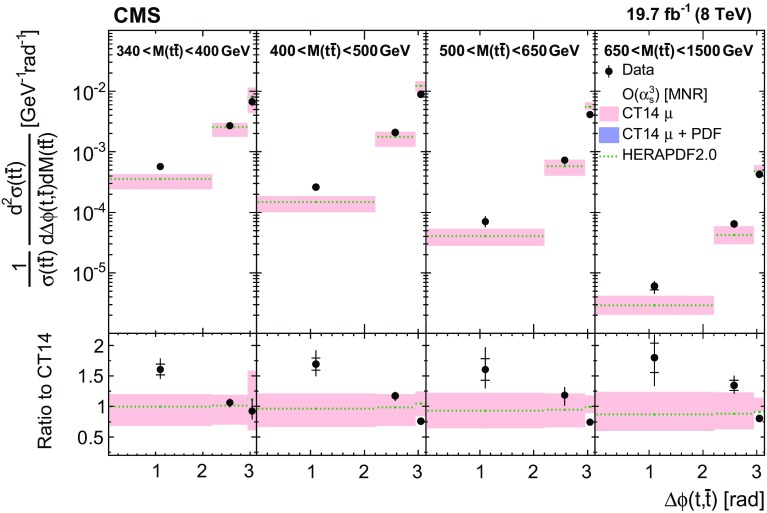
Comparison of the measured normalized $\mathrm{t}\overline{\mathrm{t}}$ double-differential cross section as a function of $\varDelta \phi (\mathrm{t},\overline{\mathrm{t}})$ in different $M(\mathrm{t}\overline{\mathrm{t}})$ ranges to NLO $O(\alpha _s^3)$ predictions. Details can be found in the caption of Fig. 8. Approximate NNLO $O(\alpha _s^4)$ predictions are not available for this cross section

In general, the systematic uncertainties are of similar size to the statistical uncertainties. The dominant systematic uncertainties are those in the signal modelling, which also are affected by the statistical uncertainties in the simulated samples that are used for the evaluation of these uncertainties. The largest experimental systematic uncertainty is the jet energy scale. The measured double-differential normalized $\mathrm{t}\overline{\mathrm{t}}$ cross sections are compared in Figs. 2, 3, 4, 5, 6, 7, 8, 9, 10, 11, 12 and 13 to theoretical predictions obtained using different MC generators and fixed-order QCD calculations. The numerical values of the measured cross sections and their uncertainties are provided in Appendix A.

### Comparison to MC models

In Fig. 2, the $p_{\mathrm {T}} (\mathrm{t})$ distribution is compared in different ranges of $|y(\mathrm{t}) |$ to predictions from MadGraph +pythia 6, powheg +pythia 6, powheg +herwig 6, and mc@nlo +herwig 6. The data distribution is softer than that of the MC expectation over almost the entire $y(\mathrm{t})$ range, except at high $|y(\mathrm{t}) |$ values. The disagreement level is the strongest for MadGraph +pythia 6, while powheg +herwig 6 describes the data best.

Figures 3 and 4 illustrate the distributions of $|y(\mathrm{t}) |$ and $|y(\mathrm{t}\overline{\mathrm{t}}) |$ in different $M(\mathrm{t}\overline{\mathrm{t}})$ ranges compared to the same set of MC models. While the agreement between the data and MC predictions is good in the lower ranges of $M(\mathrm{t}\overline{\mathrm{t}})$, the simulation starts to deviate from the data at higher $M(\mathrm{t}\overline{\mathrm{t}})$, where the predictions are more central than the data for $y(\mathrm{t})$ and less central for $y(\mathrm{t}\overline{\mathrm{t}})$.

In Fig. 5, the $\varDelta \eta (\mathrm{t},\overline{\mathrm{t}})$ distribution is compared in the same $M(\mathrm{t}\overline{\mathrm{t}})$ ranges to the MC predictions. For all generators there is a discrepancy between the data and simulation for the medium $M(\mathrm{t}\overline{\mathrm{t}})$ bins, where the predicted $\varDelta \eta (\mathrm{t},\overline{\mathrm{t}})$ values are too low. The disagreement is the strongest for MadGraph +pythia 6.

Figures 6 and 7 illustrate the comparison of the distributions of $p_{\mathrm {T}} (\mathrm{t}\overline{\mathrm{t}})$ and $\varDelta \phi (\mathrm{t},\overline{\mathrm{t}})$ in the same $M(\mathrm{t}\overline{\mathrm{t}})$ ranges to the MC models. For the $p_{\mathrm {T}} (\mathrm{t}\overline{\mathrm{t}})$ distribution (Fig. 6), which is sensitive to radiation, none of the MC generators provide a good description. The largest differences are observed between the data and powheg +pythia 6 for the highest values of $p_{\mathrm {T}} (\mathrm{t}\overline{\mathrm{t}})$, where the predictions lie above the data. For the $\varDelta \phi (\mathrm{t},\overline{\mathrm{t}})$ distribution (Fig. 7), all MC models describe the data reasonably well.

In order to perform a quantitative comparison of the measured cross sections to all considered MC generators, $\chi ^2$ values are calculated as follows:

4 $$\begin{aligned} \chi ^2 = \mathbf {R}^{T}_{N-1} \mathbf {Cov}^{-1}_{N-1} \mathbf {R}_{N-1}, \end{aligned}$$

where $\mathbf {R}_{N-1}$ is the column vector of the residuals calculated as the difference of the measured cross sections and the corresponding predictions obtained by discarding one of the *N* bins, and $\mathbf {Cov}_{N-1}$ is the $(N-1)\times (N-1)$ submatrix obtained from the full covariance matrix by discarding the corresponding row and column. The matrix $\mathbf {Cov}_{N-1}$ obtained in this way is invertible, while the original covariance matrix $\mathbf {Cov}$ is singular. This is because for normalized cross sections one loses one degree of freedom, as can be deduced from Eq. (3). The covariance matrix $\mathbf {Cov}$ is calculated as:

5 $$\begin{aligned} \mathbf {Cov} = \mathbf {Cov}^\mathrm{{unf}} + \mathbf {Cov}^\mathrm{{syst}}, \end{aligned}$$

where $\mathbf {Cov}^\text {unf}$ and $\mathbf {Cov}^\mathrm{{syst}}$ are the covariance matrices accounting for the statistical uncertainties from the unfolding, and the systematic uncertainties, respectively. The systematic covariance matrix $\mathbf {Cov}^\mathrm{{syst}}$ is calculated as:

6 $$\begin{aligned} \mathbf {Cov}^\mathrm{{syst}}_{ij}= & {} \sum _{k}C_{j,k}C_{i,k} \nonumber \\&+\, \frac{1}{2} \left( \sum _{k'}C^{+}_{j,k'}C^{+}_{i,k'} + \sum _{k'}C^{-}_{j,k'}C^{-}_{i,k'} \right) ,\nonumber \\&\qquad 1 \le i \le N, \quad 1 \le j \le N, \end{aligned}$$

where $C_{i,k}$ stands for the systematic uncertainty from source *k* in the *i*th bin, which consists of one variation only, and $C^{+}_{i,k'}$ and $C^{-}_{i,k'}$ stand for the positive and negative variations, respectively, of the systematic uncertainty due to source $k'$ in the *i*th bin. The sums run over all sources of the corresponding systematic uncertainties. All systematic uncertainties are treated as additive, i.e. the relative uncertainties are used to scale the corresponding measured value in the construction of $\mathbf {Cov}^\mathrm{{syst}}$. This treatment is consistent with the cross section normalization. The cross section measurements for different pairs of observables are statistically and systematically correlated. No attempt is made to quantify the correlations between bins from different double-differential distributions. Thus, quantitative comparisons between theoretical predictions and the data can only be made for individual distributions.

The obtained $\chi ^2$ values, together with the corresponding numbers of degrees of freedom (dof), are listed in Table 1. From these values one can conclude that none of the considered MC generators is able to correctly describe all distributions. In particular, for $[\varDelta \eta (\mathrm{t},\overline{\mathrm{t}}), M(\mathrm{t}\overline{\mathrm{t}}) ]$ and $[p_{\mathrm {T}} (\mathrm{t}\overline{\mathrm{t}}), M(\mathrm{t}\overline{\mathrm{t}}) ]$, the $\chi ^2$ values are relatively large for all MC generators. The best agreement with the data is provided by powheg +herwig 6.

**Table 1:** The $\chi ^2$ values and dof of the measured normalized double-differential $\mathrm{t}\overline{\mathrm{t}}$ cross sections with respect to the various MC predictions

Cross section variables	dof	$\chi ^2$			
		MadGraph +pythia 6	powheg +pythia 6	powheg +herwig 6	mc@nlo +herwig 6
$[p_{\mathrm {T}} (\mathrm{t}), y(\mathrm{t}) ]$	15	96	58	14	46
$[y(\mathrm{t}), M(\mathrm{t}\overline{\mathrm{t}}) ]$	15	53	20	13	21
$[y(\mathrm{t}\overline{\mathrm{t}}), M(\mathrm{t}\overline{\mathrm{t}}) ]$	15	19	21	15	22
$[\varDelta \eta (\mathrm{t},\overline{\mathrm{t}}), M(\mathrm{t}\overline{\mathrm{t}}) ]$	11	163	33	20	39
$[p_{\mathrm {T}} (\mathrm{t}\overline{\mathrm{t}}), M(\mathrm{t}\overline{\mathrm{t}}) ]$	15	31	83	30	33
$[\varDelta \phi (\mathrm{t},\overline{\mathrm{t}}), M(\mathrm{t}\overline{\mathrm{t}}) ]$	11	21	21	10	17

### Comparison to fixed-order calculations

Fixed-order theoretical calculations for fully differential cross sections in inclusive $\mathrm{t}\overline{\mathrm{t}}$ production are publicly available at NLO $O(\alpha _s^3)$ in the fixed-flavour number scheme [18], where $\alpha _s$ is the strong coupling strength. The exact fully differential NNLO $O(\alpha _s^4)$ calculations for $\mathrm{t}\overline{\mathrm{t}}$ production have recently appeared in the literature [56, 57], but are not yet publicly available. For higher orders, the cross sections as functions of single-particle kinematic variables have been calculated at approximate NNLO $O(\alpha _s^4)$ [19] and next-to-next-to-next-to-leading-order $O(\alpha _s^5)$ [58], using methods of threshold resummation beyond the leading-logarithmic accuracy.

The measured cross sections are compared with NLO QCD predictions based on several PDF sets. The predictions are calculated using the mcfm program (version 6.8) [59] and a number of the latest PDF sets, namely: ABM11 [60], CJ15 [61], CT14 [62], HERAPDF2.0 [63], JR14 [64], MMHT2014 [65], and NNPDF3.0 [66], available via the lhapdf interface (version 6.1.5) [67]. The number of active flavours is set to $n_f = 5$ and the top quark pole mass $m_{\mathrm{t}} = 172.5$ $\,\text {GeV}$ is used. The effect of using $n_f = 6$ in the PDF evolution, i.e. treating the top quark as a massless parton above threshold (as was done, e.g. in HERAPDF2.0 [63]), has been checked and the differences were found to be ${<}0.1\%$ (also see the corresponding discussion in Ref. [66]). The renormalization and factorization scales are chosen to be $\mu _\mathrm {r} = \mu _\mathrm {f} = \sqrt{\smash [b]{m_{\mathrm{t}}^2+[p_{\mathrm {T}} ^2({\mathrm{t}})+p_{\mathrm {T}} ^2(\overline{\mathrm{t}})]/2}}$, whereas $\alpha _s$ is set to the value used for the corresponding PDF extraction. The theoretical uncertainty is estimated by varying $\mu _\mathrm {r}$ and $\mu _\mathrm {f}$ independently up and down by a factor of 2, subject to the additional restriction that the ratio $\mu _\mathrm {r} / \mu _\mathrm {f}$ be between 0.5 and 2 [68] (referred to hereafter as scale uncertainties). These uncertainties are supposed to estimate the missing higher-order corrections. The PDF uncertainties are taken into account in the theoretical predictions for each PDF set. The PDF uncertainties of CJ15  [61] and CT14  [62], evaluated at 90% CL, are rescaled to the 68% CL. The uncertainties in the normalized $\mathrm{t}\overline{\mathrm{t}}$ cross sections originating from $\alpha _s$ and $m_{\mathrm{t}}$ are found to be negligible (${<}1\%$) compared to the current data precision and thus are not considered.

For the double-differential cross section as a function of $p_{\mathrm {T}} (\mathrm{t})$ and $y(\mathrm{t})$, approximate NNLO predictions [19] are obtained using the DiffTop program [4, 69, 70]. In this calculation, the scales are set to $\mu _\mathrm {r} = \mu _\mathrm {f} = \sqrt{\smash [b]{m_{\mathrm{t}}^2+p_{\mathrm {T}} ^2({\mathrm{t}})}}$ and NNLO variants of the PDF sets are used. For the ABM PDFs, the recent version ABM12 [71] is used, which is available only at NNLO. Predictions using DiffTop are not available for the rest of the measured cross sections that involve $\mathrm{t}\overline{\mathrm{t}}$ kinematic variables.

A quantitative comparison of the measured double-differential cross sections to the theoretical predictions is performed by evaluating the $\chi ^2$ values, as described in Sect. 8.1. The results are listed in Tables 2 and 3 for the NLO and approximate NNLO calculations, respectively. For the NLO predictions, additional $\chi ^2$ values are reported including the corresponding PDF uncertainties, i.e. Eq. (5) becomes $\mathbf {Cov} = \mathbf {Cov}^\text {unf} + \mathbf {Cov}^\text {syst} + \mathbf {Cov}^\mathrm {PDF}$, where $\mathbf {Cov}^\mathrm {PDF}$ is a covariance matrix that accounts for the PDF uncertainties. Theoretical uncertainties from scale variations are not included in this $\chi ^2$ calculation. The NLO predictions with recent global PDFs using LHC data, namely MMHT2014, CT14, and NNPDF3.0, are found to describe the $p_{\mathrm {T}} (\mathrm{t})$, $y(\mathrm{t})$, and $y(\mathrm{t}\overline{\mathrm{t}})$ cross sections reasonably, as illustrated by the $\chi ^2$ values. The CJ15 PDF set also provides a good description of these cross sections, although it does not include LHC data [61]. The ABM11, JR14, and HERAPDF2.0 sets yield a poorer description of the data. Large differences between the data and the nominal NLO calculations are observed for the $\varDelta \eta (\mathrm{t},\overline{\mathrm{t}})$, $p_{\mathrm {T}} (\mathrm{t}\overline{\mathrm{t}})$, and $\varDelta \phi (\mathrm{t},\overline{\mathrm{t}})$ cross sections. It is noteworthy that the scale uncertainties in the predictions, which are of comparable size or exceed the experimental uncertainties, are not taken into account in the $\chi ^2$ calculations. The $p_{\mathrm {T}} (\mathrm{t}\overline{\mathrm{t}})$ and $\varDelta \phi (\mathrm{t},\overline{\mathrm{t}})$ normalized cross sections are represented at LO $O(\alpha _s^2)$ by delta functions, and nontrivial shapes appear at $O(\alpha _s^3)$, thus resulting in large NLO scale uncertainties [18]. Compared to the NLO predictions, the approximate NNLO predictions using NNLO PDF sets (where available) provide an improved description of the $p_{\mathrm {T}} (\mathrm{t})$ cross sections in different $|y(\mathrm{t}) |$ ranges.

**Table 2:** The $\chi ^2$ values and dof of the double-differential normalized $\mathrm{t}\overline{\mathrm{t}}$ cross sections with respect to NLO $O(\alpha _s^3)$ theoretical calculations [18] using different PDF sets. The $\chi ^2$ values that include PDF uncertainties are shown in parentheses

Cross section variables	dof	$\chi ^2$ NLO $O(\alpha _s^3)$ (including PDF uncertainties)						
		HERAPDF2.0	MMHT2014	CT14	NNPDF3.0	ABM11	JR14	CJ15
$[p_{\mathrm {T}} (\mathrm{t}), y(\mathrm{t}) ]$	15	46 (40)	26 (24)	24 (21)	28 (25)	62 (51)	47 (47)	27 (24)
$[y(\mathrm{t}), M(\mathrm{t}\overline{\mathrm{t}}) ]$	15	52 (44)	22 (20)	19 (18)	14 (14)	71 (55)	44 (44)	26 (24)
$[y(\mathrm{t}\overline{\mathrm{t}}), M(\mathrm{t}\overline{\mathrm{t}}) ]$	15	29 (25)	15 (15)	16 (15)	10 (10)	42 (31)	25 (25)	16 (16)
$[\varDelta \eta (\mathrm{t},\overline{\mathrm{t}}), M(\mathrm{t}\overline{\mathrm{t}}) ]$	11	46 (43)	31 (31)	32 (31)	45 (42)	48 (44)	39 (39)	33 (33)
$[p_{\mathrm {T}} (\mathrm{t}\overline{\mathrm{t}}), M(\mathrm{t}\overline{\mathrm{t}}) ]$	15	485 (429)	377 (310)	379 (264)	251 (212)	553 (426)	428 (415)	413 (398)
$[\varDelta \phi (\mathrm{t},\overline{\mathrm{t}}), M(\mathrm{t}\overline{\mathrm{t}}) ]$	11	354 (336)	293 (272)	296 (259)	148 (143)	386 (335)	329 (324)	312 (308)

**Table 3:** The $\chi ^2$ values and dof of the double-differential normalized $\mathrm{t}\overline{\mathrm{t}}$ cross sections with respect to approximate NNLO $O(\alpha _s^4)$ theoretical calculations [4, 19, 69, 70] using different PDF sets

Cross section variables	dof	$\chi ^2$ approximate NNLO $O(\alpha _s^4)$					
		HERAPDF2.0	MMHT2014	CT14	NNPDF3.0	ABM12	JR14
$[p_{\mathrm {T}} (\mathrm{t}), y(\mathrm{t}) ]$	15	22	11	13	15	54	44

To visualize the comparison of the measurements to the theoretical predictions, the results obtained using the NLO and approximate NNLO calculations with the CT14 PDF set are compared to the measured $p_{\mathrm {T}} (\mathrm{t})$ cross sections in different $|y(\mathrm{t}) |$ ranges in Fig. 8. To further illustrate the sensitivity to PDFs, the nominal values of the NLO predictions using HERAPDF2.0 are shown as well. Similar comparisons, in regions of $M(\mathrm{t}\overline{\mathrm{t}})$, for the $|y(\mathrm{t}) |$, $|y(\mathrm{t}\overline{\mathrm{t}}) |$, $\varDelta \eta (\mathrm{t},\overline{\mathrm{t}})$, $p_{\mathrm {T}} (\mathrm{t}\overline{\mathrm{t}})$, and $\varDelta \phi (\mathrm{t},\overline{\mathrm{t}})$ cross sections are presented in Figs. 9, 10, 11, 12 and 13. Considering the scale uncertainties in the predictions, the agreement between the measurement and predictions is reasonable for all distributions. For the $p_{\mathrm {T}} (\mathrm{t})$, $y(\mathrm{t})$, and $y(\mathrm{t}\overline{\mathrm{t}})$ cross sections, the scale uncertainties in the predictions reach 4% at maximum. They increase to 8% for the $\varDelta \eta (\mathrm{t},\overline{\mathrm{t}})$ cross section, and vary within $20\text {--}50\%$ for the $p_{\mathrm {T}} (\mathrm{t}\overline{\mathrm{t}})$ and $\varDelta \phi (\mathrm{t},\overline{\mathrm{t}})$ cross sections, where larger differences between data and predictions are observed. For the $p_{\mathrm {T}} (\mathrm{t})$, $y(\mathrm{t})$, and $y(\mathrm{t}\overline{\mathrm{t}})$ cross sections, the PDF uncertainties as estimated from the CT14 PDF set are of the same size or larger than the scale uncertainties. The HERAPDF2.0 predictions are mostly outside the total CT14 uncertainty band, showing also some visible shape differences with respect to CT14. The approximate NNLO predictions provide an improved description of the $p_{\mathrm {T}} (\mathrm{t})$ shape.

The data-to-theory comparisons illustrate the power of the measured normalized cross sections as a function of $[p_{\mathrm {T}} (\mathrm{t}), y(\mathrm{t}) ]$, $[y(\mathrm{t}), M(\mathrm{t}\overline{\mathrm{t}}) ]$, and $[y(\mathrm{t}\overline{\mathrm{t}}), M(\mathrm{t}\overline{\mathrm{t}}) ]$ to eventually distinguish between modern PDF sets. Such a study is performed on these data and described in the next section. The remaining measured normalized cross sections as a function of $[\varDelta \eta (\mathrm{t},\overline{\mathrm{t}}), M(\mathrm{t}\overline{\mathrm{t}}) ]$, $[p_{\mathrm {T}} (\mathrm{t}\overline{\mathrm{t}}), M(\mathrm{t}\overline{\mathrm{t}}) ]$, and $[\varDelta \phi (\mathrm{t},\overline{\mathrm{t}}), M(\mathrm{t}\overline{\mathrm{t}}) ]$ could be used for this purpose as well, once higher-order QCD calculations become publicly available to match the data precision. Moreover, since the latter distributions are more sensitive to QCD radiation, they will provide additional input in testing improvements to the perturbative calculations.

## The PDF fit

The double-differential normalized $\mathrm{t}\overline{\mathrm{t}}$ cross sections are used in a PDF fit at NLO, together with the combined HERA inclusive deep inelastic scattering (DIS) data [63] and the CMS measurement of the $\mathrm {W}^\pm$ boson charge asymmetry at $\sqrt{s} = 8$ $\,\text {TeV}$  [72]. The fitted PDFs are also compared to the ones obtained in the recently published CMS measurement of inclusive jet production at 8$\,\text {TeV}$  [8]. The xFitter program (formerly known as HERAFitter) [73] (version 1.2.0), an open-source QCD fit framework for PDF determination, is used. The precise HERA DIS data, obtained from the combination of individual H1 and ZEUS results, are directly sensitive to the valence and sea quark distributions and probe the gluon distribution through scaling violations. Therefore, these data form the core of all PDF fits. The CMS $\mathrm {W}^\pm$ boson charge asymmetry data provide further constraints on the valence quark distributions, as discussed in Ref. [72]. The measured double-differential normalized $\mathrm{t}\overline{\mathrm{t}}$ cross sections are included in the fit to constrain the gluon distribution at high *x* values. The typical probed *x* values can be estimated using the LO kinematic relation $x = (M(\mathrm{t}\overline{\mathrm{t}})/\sqrt{s})\exp {[\pm y(\mathrm{t}\overline{\mathrm{t}})]}$. Therefore, the present measurement is expected to be sensitive to *x* values in the region $0.01 \lesssim x \lesssim 0.25$, as estimated using the highest or lowest $|y(\mathrm{t}\overline{\mathrm{t}}) |$ or $M(\mathrm{t}\overline{\mathrm{t}})$ bins and taking the low or high bin edge where the cross section is largest (see Table 11).

### Details of the PDF fit

The scale evolution of partons is calculated through DGLAP equations [74–80] at NLO, as implemented in the qcdnum program [81] (version 17.01.11). The Thorne–Roberts [82–84] variable-flavour number scheme at NLO is used for the treatment of the heavy-quark contributions. The number of flavours is set to 5, with c and b quark mass parameters $M_{\mathrm{c}}= 1.47$ $\,\text {GeV}$ and $M_{\mathrm{b}} = 4.5$ $\,\text {GeV}$  [63]. The theoretical predictions for the $\mathrm {W}^\pm$ boson charge asymmetry data are calculated at NLO [85] using the mcfm program, which is interfaced with ApplGrid (version 1.4.70) [86], as described in Ref. [72]. For the DIS and $\mathrm {W}^\pm$ boson charge asymmetry data $\mu _\mathrm {r}$ and $\mu _\mathrm {f}$ are set to *Q*, which denotes the four-momentum transfer in the case of the DIS data, and the mass of the $\mathrm {W}^{\pm }$ boson in the case of the $\mathrm {W}^\pm$ boson charge asymmetry. The theoretical predictions for the $\mathrm{t}\overline{\mathrm{t}}$ cross sections are calculated as described in Sect. 8.2 and included in the fit using the mcfm and ApplGrid programs. The strong coupling strength is set to $\alpha _s(m_{\mathrm{Z}}) = 0.118$. The $Q^2$ range of the HERA data is restricted to $Q^2 > Q^2_\text {min} = 3.5\,\text {GeV} ^2$ [63].

The procedure for the determination of the PDFs follows the approach of HERAPDF2.0  [63]. The parametrized PDFs are the gluon distribution $x\mathrm{g} (x)$, the valence quark distributions $x\mathrm{u}_v(x)$ and $x\mathrm{d}_v(x)$, and the $\mathrm{u}$- and $\mathrm{d}$-type antiquark distributions $x\overline{U}(x)$ and $x\overline{D}(x)$. At the initial QCD evolution scale $\mu _\mathrm {f0}^2 = 1.9\,\text {GeV} ^2$, the PDFs are parametrized as:

7 $$\begin{aligned} x\mathrm{g} (x)&= A_{\mathrm{g}} x^{B_{\mathrm{g}}}\,(1-x)^{C_{\mathrm{g}}}\, (1 + E_{\mathrm{g}} x^2 + F_{\mathrm{g}} x^3) - A'_{\mathrm{g}} x^{B'_{\mathrm{g}}}\,(1-x)^{C'_{\mathrm{g}}}, \nonumber \\ x\mathrm{u}_v(x)&= A_{\mathrm{u}_v}x^{B_{\mathrm{u}_v}}\,(1-x)^{C_{\mathrm{u}_v}}\,(1+D_{\mathrm{u}_v}x+E_{\mathrm{u}_v}x^2) ,\nonumber \\ x\mathrm{d}_v(x)&= A_{\mathrm{d}_v}x^{B_{\mathrm{d}_v}}\,(1-x)^{C_{\mathrm{d}_v}},\nonumber \\ x\overline{U}(x)&= A_{\overline{U}}x^{B_{\overline{U}}}\, (1-x)^{C_{\overline{U}}}\, (1+D_{\overline{U}}x+F_{\overline{U}}x^3), \nonumber \\ x\overline{D}(x)&= A_{\overline{D}}x^{B_{\overline{D}}} \, (1-x)^{C_{\overline{D}}}, \end{aligned}$$

assuming the relations $x\overline{U}(x) = x\overline{\mathrm{u}}(x)$ and $x\overline{D}(x) = x\overline{\mathrm{d}}(x) + x\overline{\mathrm{s}}(x)$. Here, $x\overline{\mathrm{u}}(x)$, $x\overline{\mathrm{d}}(x)$, and $x\overline{\mathrm{s}}(x)$ are the up, down, and strange antiquark distributions, respectively. The sea quark distribution is defined as x$\varSigma (x)=x\overline{\mathrm{u}}(x)+x\overline{\mathrm{d}}(x)+x \overline{\mathrm{s}}(x)$. The normalization parameters $A_{\mathrm{u}_{{v}}}$, $A_{\mathrm{d}_{v}}$, and $A_{\mathrm{g}}$ are determined by the QCD sum rules. The *B* and $B'$ parameters determine the PDFs at small *x*, and the *C* parameters describe the shape of the distributions as $x\,{\rightarrow }\,1$. The parameter $C'_{\mathrm{g}}$ is fixed to 25 [87]. Additional constraints $B_{\overline{{U}}} = B_{\overline{{D}}}$ and $A_{\overline{{U}}} = A_{\overline{{D}}}(1 - f_{\mathrm{s}})$ are imposed to ensure the same normalization for the $x\overline{\mathrm{u}}$ and $x\overline{\mathrm{d}}$ distributions as $x \rightarrow 0$. The strangeness fraction $f_{\mathrm{s}} = x\overline{\mathrm{s}}/( x\overline{\mathrm{d}}+ x\overline{\mathrm{s}})$ is fixed to $f_{\mathrm{s}}=0.4$ as in the HERAPDF2.0 analysis [63]. This value is consistent with the determination of the strangeness fraction when using the CMS measurements of $\mathrm {W}+{\mathrm{c}}$ production [88].

The parameters in Eq. (7) are selected by first fitting with all *D*, *E*, and *F* parameters set to zero, and then including them independently one at a time in the fit. The improvement in the $\chi ^2$ of the fit is monitored and the procedure is stopped when no further improvement is observed. This leads to an 18-parameter fit. The $\chi ^2$ definition used for the HERA DIS data follows that of Eq. (32) in Ref. [63]. It includes an additional logarithmic term that is relevant when the estimated statistical and uncorrelated systematic uncertainties in the data are rescaled during the fit [89]. For the CMS $\mathrm {W}^\pm$ boson charge asymmetry and $\mathrm{t}\overline{\mathrm{t}}$ data presented here a $\chi ^2$ definition without such a logarithmic term is employed. The full covariance matrix representing the statistical and uncorrelated systematic uncertainties of the data is used in the fit. The correlated systematic uncertainties are treated through nuisance parameters. For each nuisance parameter a penalty term is added to the $\chi ^2$, representing the prior knowledge of the parameter. The treatment of the experimental uncertainties for the HERA DIS and CMS $\mathrm {W}^\pm$ boson charge asymmetry data follows the prescription given in Refs. [63] and [72], respectively. The treatment of the experimental uncertainties in the $\mathrm{t}\overline{\mathrm{t}}$ double-differential cross section measurements follows the prescription given in Sect. 8.1. The experimental systematic uncertainties owing to the PDFs are omitted in the PDF fit.

The PDF uncertainties are estimated according to the general approach of HERAPDF2.0  [63] in which the fit, model, and parametrization uncertainties are taken into account. Fit uncertainties are determined using the tolerance criterion of $\varDelta \chi ^2 =1$. Model uncertainties arise from the variations in the values assumed for the b and c quark mass parameters of $4.25\le M_{\mathrm{b}}\le 4.75\,\text {GeV}$ and $1.41\le M_{\mathrm{c}}\le 1.53\,\text {GeV}$, the strangeness fraction $0.3 \le f_{\mathrm{s}} \le 0.4$, and the value of $Q^2_{\text {min}}$ imposed on the HERA data. The latter is varied within $2.5 \le Q^2_{\text {min}}\le 5.0\,\text {GeV} ^2$, following Ref. [63]. The parametrization uncertainty is estimated by extending the functional form in Eq. (7) of all parton distributions with additional parameters *D*, *E*, and *F* added one at a time. Furthermore, $\mu _\mathrm {f0}^2$ is changed to 1.6 and $2.2\,\text {GeV} ^2$. The parametrization uncertainty is constructed as an envelope at each *x* value, built from the maximal differences between the PDFs resulting from the central fit and all parametrization variations. This uncertainty is valid in the *x* range covered by the PDF fit to the data. The total PDF uncertainty is obtained by adding the fit, model, and parametrization uncertainties in quadrature. In the following, the quoted uncertainties correspond to 68% CL.

### Impact of the double-differential $\mathrm{t}\overline{\mathrm{t}}$ cross section measurements

The PDF fit is first performed using only the HERA DIS and CMS $\mathrm {W}^\pm$ boson charge asymmetry data. To demonstrate the added value of the double-differential normalized $\mathrm{t}\overline{\mathrm{t}}$ cross sections, $[p_{\mathrm {T}} (\mathrm{t}), y(\mathrm{t}) ]$, $[y(\mathrm{t}), M(\mathrm{t}\overline{\mathrm{t}}) ]$, and $[y(\mathrm{t}\overline{\mathrm{t}}), M(\mathrm{t}\overline{\mathrm{t}}) ]$ measurements are added to the fit one at a time. The global and partial $\chi ^2$ values for all variants of the fit are listed in Table 4, illustrating the consistency among the input data. The DIS data show $\chi ^2$/dof values slightly larger than unity. This is similar to what is observed and investigated in Ref. [63]. Fit results consistent with those from Ref. [72] are obtained using the $\mathrm {W}^\pm$ boson charge asymmetry measurements.

**Table 4:** The global and partial $\chi ^2$/dof values for all variants of the PDF fit. The variant of the fit that uses the DIS and $\mathrm {W}^\pm$ boson charge asymmetry data only is denoted as ‘Nominal fit’. Each double-differential $\mathrm{t}\overline{\mathrm{t}}$ cross section is added ($+$) to the nominal data, one at a time. For the HERA measurements, the energy of the proton beam, $E_{\mathrm {p}}$, is listed for each data set, with the electron energy being $E_{\mathrm {e}}=27.5\,\text {GeV}$, CC and NC stand for charged and neutral current, respectively. The correlated $\chi ^2$ and the log-penalty $\chi ^2$ entries refer to the $\chi ^2$ contributions from the nuisance parameters and from the logarithmic term, respectively, as described in the text

Data sets	$\chi ^2$/dof			
	Nominal fit	$+ [p_{\mathrm {T}} (\mathrm{t}), y(\mathrm{t}) ]$	$+ [y(\mathrm{t}), M(\mathrm{t}\overline{\mathrm{t}}) ]$	$+ [y(\mathrm{t}\overline{\mathrm{t}}), M(\mathrm{t}\overline{\mathrm{t}}) ]$
CMS double-differential $\mathrm{t}\overline{\mathrm{t}}$		10 / 15	7.4 / 15	7.6 / 15
HERA CC $\mathrm {e}^-\mathrm {p}$, $E_{\mathrm {p}}=920$ $\,\text {GeV}$	57 / 42	56 / 42	56 / 42	57 / 42
HERA CC $\mathrm {e}^+\mathrm {p}$, $E_{\mathrm {p}}=920$ $\,\text {GeV}$	44 / 39	44 / 39	44 / 39	43 / 39
HERA NC $\mathrm {e}^-\mathrm {p}$, $E_{\mathrm {p}}=920$ $\,\text {GeV}$	219 / 159	219 / 159	219 / 159	218 / 159
HERA NC $\mathrm {e}^+\mathrm {p}$, $E_{\mathrm {p}}=920$ $\,\text {GeV}$	440 / 377	437 / 377	439 / 377	441 / 377
HERA NC $\mathrm {e}^+\mathrm {p}$, $E_{\mathrm {p}}=820$ $\,\text {GeV}$	69 / 70	68 / 70	68 / 70	69 / 70
HERA NC $\mathrm {e}^+\mathrm {p}$, $E_{\mathrm {p}}=575$ $\,\text {GeV}$	221 / 254	220 / 254	221 / 254	221 / 254
HERA NC $\mathrm {e}^+\mathrm {p}$, $E_{\mathrm {p}}=460$ $\,\text {GeV}$	219 / 204	219 / 204	219 / 204	219 / 204
CMS $\mathrm {W}^{\pm }$ asymmetry	4.7 / 11	4.6 / 11	4.8 / 11	4.9 / 11
Correlated $\chi ^2$	82	87	91	89
Log-penalty $\chi ^2$	$-2.5$	$+2.6$	$-2.2$	$-3.3$
Total $\chi ^2$/dof	1352 / 1138	1368 / 1153	1368 / 1153	1366 / 1153

**Fig. 14:**
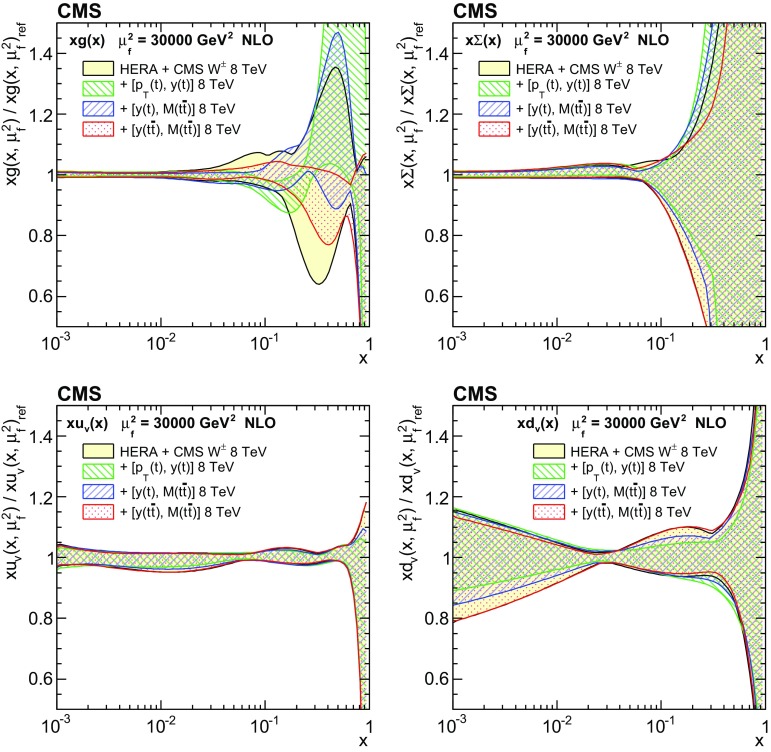
The gluon (*upper left*), sea quark (*upper right*), u valence quark (*lower left*), and d valence quark (*lower right*) PDFs at $\mu _\mathrm{{f}}^2=30{,} 000\,\text {GeV} ^2$, as obtained in all variants of the PDF fit, normalized to the results from the fit using the HERA DIS and CMS $\mathrm {W}^\pm$ boson charge asymmetry measurements only. The *shaded*, *hatched*, and *dotted areas* represent the total uncertainty in each of the fits

**Fig. 15:**
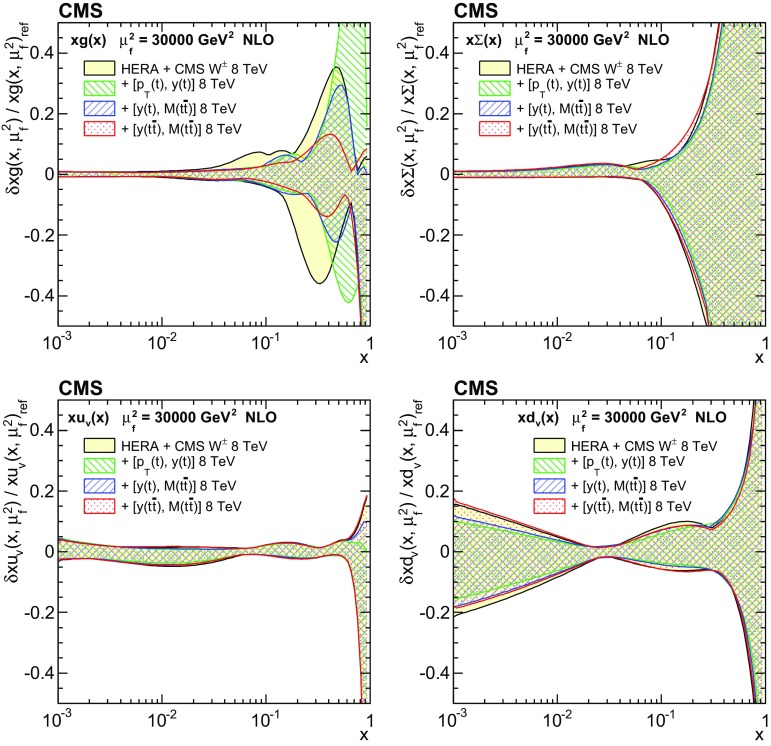
Relative total uncertainties of the gluon (*upper left*), sea quark (*upper right*), u valence quark (*lower left*), and d valence quark (*lower right*) distributions at $\mu _\mathrm{{f}}^2=30{,} 000\,\text {GeV} ^2$, shown by *shaded*, *hatched*, and *dotted areas*, as obtained in all variants of the PDF fit

The resulting gluon, valence quark, and sea quark distributions are shown in Fig. 14 at the scale $\mu _\mathrm{{f}}^2=30{,} 000\,\text {GeV} ^2 \simeq m_{\mathrm{t}}^2$ relevant for $\mathrm{t}\overline{\mathrm{t}}$ production. For a direct comparison, the distributions for all variants of the fit are normalized to the results from the fit using only the DIS and $\mathrm {W}^\pm$ boson charge asymmetry data. The reduction of the uncertainties is further illustrated in Fig. 15. The uncertainties in the gluon distribution at $x>0.01$ are significantly reduced once the $\mathrm{t}\overline{\mathrm{t}}$ data are included in the fit. The largest improvement comes from the $[y(\mathrm{t}\overline{\mathrm{t}}), M(\mathrm{t}\overline{\mathrm{t}}) ]$ cross section by which the total gluon PDF uncertainty is reduced by more than a factor of two at $x \simeq 0.3$. This value of *x* is at the edge of kinematic reach of the current $\mathrm{t}\overline{\mathrm{t}}$ measurement. At higher values $x \gtrsim 0.3$, the gluon distribution is not directly constrained by the data and should be considered as an extrapolation that relies on the PDF parametrization assumptions. No substantial effects on the valence quark and sea quark distributions are observed. The variation of $\mu _\mathrm {r}$ and $\mu _\mathrm {f}$ in the prediction of the normalized $\mathrm{t}\overline{\mathrm{t}}$ cross sections has been performed and the effect on the fitted PDFs is found to be well within the total uncertainty.

**Fig. 16:**
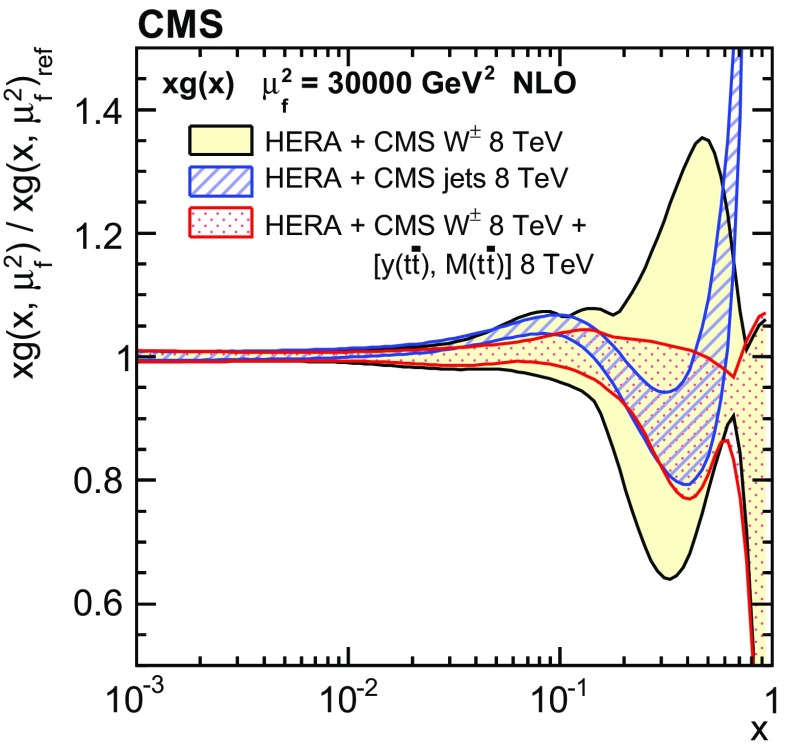
The gluon distribution at $\mu _\mathrm{{f}}^2=30{,} 000\,\text {GeV} ^2$, as obtained from the PDF fit to the HERA DIS data and CMS $\mathrm {W}^\pm$ boson charge asymmetry measurements (*shaded area*), the CMS inclusive jet production cross sections (*hatched area*), and the $\mathrm {W}^\pm$ boson charge asymmetry plus the double-differential $\mathrm{t}\overline{\mathrm{t}}$ cross section (*dotted area*). All presented PDFs are normalized to the results from the fit using the DIS and $\mathrm {W}^\pm$ boson charge asymmetry measurements. The *shaded*, *hatched*, and *dotted areas* represent the total uncertainty in each of the fits

The gluon distribution obtained from fitting the measured $[y(\mathrm{t}\overline{\mathrm{t}}), M(\mathrm{t}\overline{\mathrm{t}}) ]$ cross section is compared in Fig. 16 to the one obtained in a similar study using the CMS measurement of inclusive jet production at 8$\,\text {TeV}$  [8]. The two results are in agreement in the probed *x* range. The constraints provided by the double-differential $\mathrm{t}\overline{\mathrm{t}}$ measurement are competitive with those from the inclusive jet data.

### Comparison to the impact of single-differential $\mathrm{t}\overline{\mathrm{t}}$ cross section measurements

**Fig. 17:**
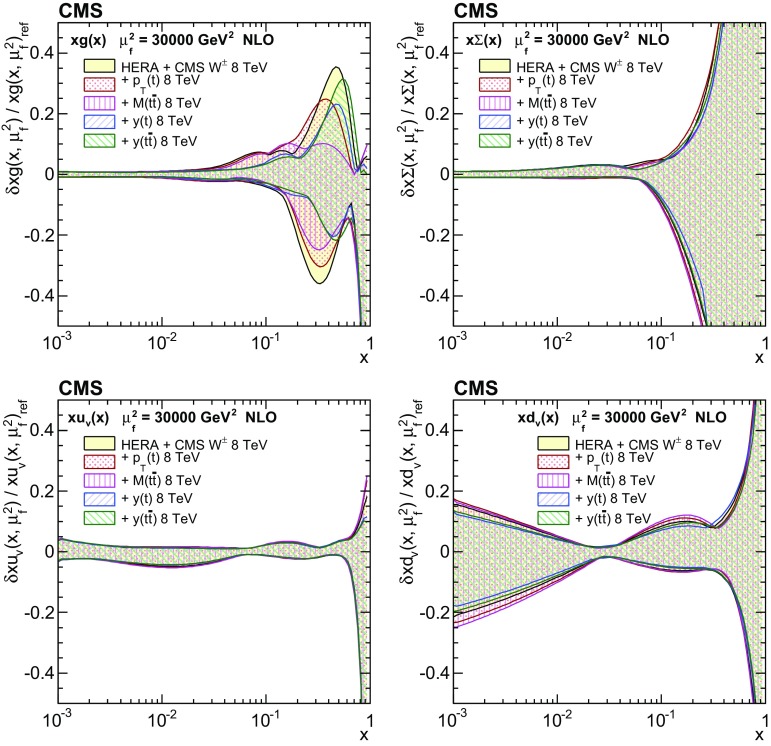
The same as in Fig. 15 for the variants of the PDF fit using the single-differential $\mathrm{t}\overline{\mathrm{t}}$ cross sections

The power of the double-differential $\mathrm{t}\overline{\mathrm{t}}$ measurement in fitting PDFs is compared with that of the single-differential analysis, where the $\mathrm{t}\overline{\mathrm{t}}$ cross section is measured as a function of $p_{\mathrm {T}} (\mathrm{t})$, $y(\mathrm{t})$, $y(\mathrm{t}\overline{\mathrm{t}})$, and $M(\mathrm{t}\overline{\mathrm{t}})$, employing in one dimension the same procedure described in this paper. The measurements are added, one at a time, to the HERA DIS and CMS $\mathrm {W}^\pm$ boson charge asymmetry data in the PDF fit. The reduction of the uncertainties for the resulting PDFs is illustrated in Fig. 17. Similar effects are observed from all measurements, with the largest impact coming from $y(\mathrm{t})$ and $y(\mathrm{t}\overline{\mathrm{t}})$. For the single-differential $\mathrm{t}\overline{\mathrm{t}}$ data one can extend the studies using the approximate NNLO calculations [4, 19, 69, 70]. An example, using the $y(\mathrm{t})$ distribution, is presented in Appendix B.

**Fig. 18:**
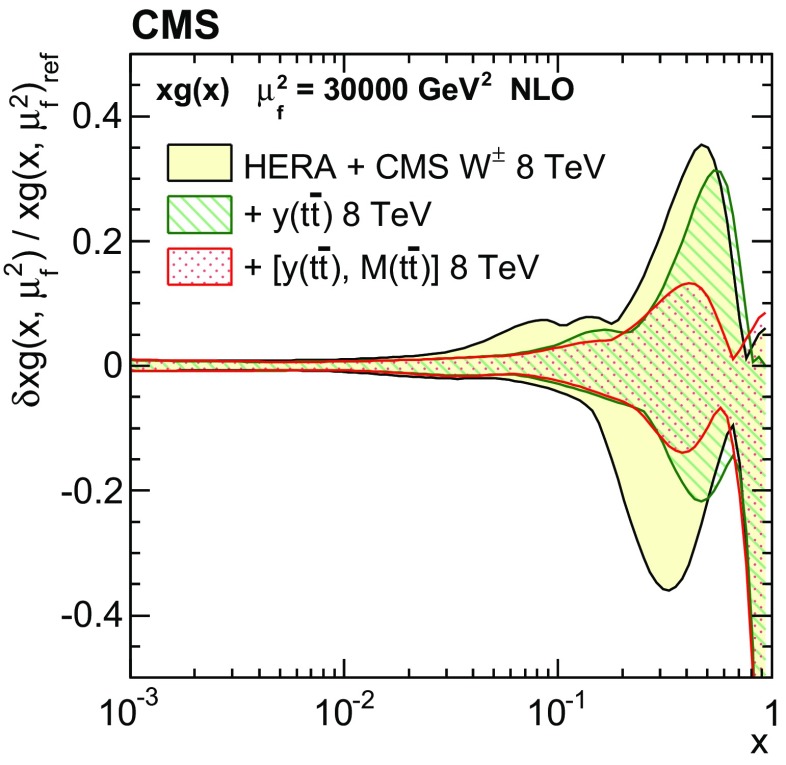
Relative total uncertainties of the gluon distribution at $\mu _\mathrm{{f}}^2=30{,} 000\,\text {GeV} ^2$, shown by *shaded* (or *hatched*) *bands*, as obtained in the PDF fit using the DIS and $\mathrm {W}^\pm$ boson charge asymmetry data only, as well as single- and double-differential $\mathrm{t}\overline{\mathrm{t}}$ cross sections

A comparison of the PDF uncertainties from the double-differential cross section as a function of $[y(\mathrm{t}\overline{\mathrm{t}}), M(\mathrm{t}\overline{\mathrm{t}}) ]$, and single-differential cross section as a function of $y(\mathrm{t}\overline{\mathrm{t}})$ is presented in Fig. 18. Only the gluon distribution is shown, since no substantial impact on the other distributions is observed (see Figs. 14, 15, 17). The total gluon PDF uncertainty becomes noticeably smaller once the double-differential cross sections are included. The observed improvement makes future PDF fits at NNLO using the fully differential calculations [56, 57], once they become available, very interesting.

## Summary

A measurement of normalized double-differential $\mathrm{t}\overline{\mathrm{t}}$ production cross sections in pp collisions at $\sqrt{s}=8\,\text {TeV}$ has been presented. The measurement is performed in the $\mathrm {e}^{\pm }\mu ^{\mp }$ final state, using data collected with the CMS detector at the LHC, corresponding to an integrated luminosity of 19.7$\,\text {fb}^{-1}$. The normalized $\mathrm{t}\overline{\mathrm{t}}$ cross section is measured in the full phase space as a function of different pairs of kinematic variables describing the top quark or $\mathrm{t}\overline{\mathrm{t}}$ system. None of the tested MC models is able to correctly describe all the double-differential distributions. The data exhibit a softer transverse momentum $p_{\mathrm {T}} (\mathrm{t})$ distribution, compared to the Monte Carlo predictions, as was reported in previous single-differential $\mathrm{t}\overline{\mathrm{t}}$ cross section measurements. The double-differential studies reveal a broader distribution of rapidity $y(\mathrm{t})$ at high $\mathrm{t}\overline{\mathrm{t}}$ invariant mass $M(\mathrm{t}\overline{\mathrm{t}})$ and a larger pseudorapidity separation $\varDelta \eta (\mathrm{t},\overline{\mathrm{t}})$ at moderate $M(\mathrm{t}\overline{\mathrm{t}})$ in data compared to simulation. The data are in reasonable agreement with next-to-leading-order predictions of quantum chromodynamics using recent sets of parton distribution functions (PDFs).

The measured double-differential cross sections have been incorporated into a PDF fit, together with other data from HERA and the LHC. Including the $\mathrm{t}\overline{\mathrm{t}}$ data, one observes a significant reduction in the uncertainties in the gluon distribution at large values of parton momentum fraction *x*, in particular when using the double-differential $\mathrm{t}\overline{\mathrm{t}}$ cross section as a function of $y(\mathrm{t}\overline{\mathrm{t}})$ and $M(\mathrm{t}\overline{\mathrm{t}})$. The constraints provided by these data are competitive with those from inclusive jet data. This improvement exceeds that from using single-differential $\mathrm{t}\overline{\mathrm{t}}$ cross section data, thus strongly suggesting the use of the double-differential $\mathrm{t}\overline{\mathrm{t}}$ measurements in PDF fits.

## Figures and Tables

**Fig. 19:**
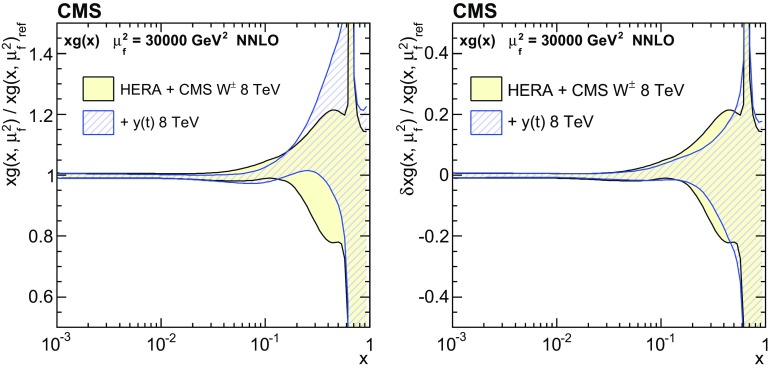
The gluon distribution (*left*) and its fractional total uncertainty (*right*) at $\mu _\mathrm{{f}}^2=30{,} 000\,\text {GeV} ^2$, as obtained in the PDF fit at NNLO using the DIS and $\mathrm {W}^\pm$ boson charge asymmetry data only, as well as $y(\mathrm{t})$ cross sections. The distributions shown in the *left panel* are normalized to the results from the fit using the DIS and $\mathrm {W}^\pm$ boson charge asymmetry data only. The total uncertainty of each distribution is shown by a *shaded* (or *hatched*) *band*

## Appendix A: Values of the normalized double-differential cross sections

Tables 5, 6, 7, 8, 9, 10, 11, 12, 13, 14, 15, 16, 17, 18, 19, 20, 21 and 22 provide the measured $\mathrm{t}\overline{\mathrm{t}}$ double-differential cross sections for all pairs of variables, including their correlation matrices of statistical uncertainties and detailed breakdown of systematic uncertainties. The b tagging systematic uncertainty is obtained by combining in quadrature variations of the data-to-simulation correction factors as a function of $p_{\mathrm {T}}$ and $|\eta |$, performed separately for jets originating from $\mathrm{b}$ quarks and other partons, as presented in Sects. 4 and 7. The PDF systematic uncertainty is obtained by combining in quadrature variations corresponding to the 52 eigenvectors of the CT10 PDF set [32].

**Table 5:** The measured normalized $\mathrm{t}\overline{\mathrm{t}}$ double-differential cross sections in different bins of $y(\mathrm{t})$ and $p_{\mathrm {T}} (\mathrm{t})$, along with their relative statistical and systematic uncertainties

$|y(\mathrm{t}) |$	$p_{\mathrm {T}} (\mathrm{t})$ ($\text {GeV}$ )	$\frac{1}{\sigma (\mathrm{t}\overline{\mathrm{t}})} \frac{\mathrm{d}^{2}\sigma (\mathrm{t}\overline{\mathrm{t}})}{\mathrm{d}y(\mathrm{t}) \mathrm{d}p_{\mathrm {T}} (\mathrm{t})}$ ($\text {GeV}$ $^{-1}$)	Stat. (%)	Syst. (%)	Bin
0–0.35	0–80	$3.08 \times 10^{-3}$	4.4	${}_{ -4.9}^{ +7.4}$	1
	80–150	$3.71 \times 10^{-3}$	3.6	${}_{ -6.2}^{ +3.5}$	2
	150–250	$1.36 \times 10^{-3}$	5.0	${}_{ -3.4}^{ +4.8}$	3
	250–600	$1.11 \times 10^{-4}$	7.7	${}_{-11.7}^{ +9.0}$	4
0.35–0.85	0–80	$2.90 \times 10^{-3}$	3.2	${}_{ -2.9}^{ +3.0}$	5
	80–150	$3.17 \times 10^{-3}$	3.0	${}_{ -4.2}^{ +2.3}$	6
	150–250	$1.17 \times 10^{-3}$	4.5	${}_{ -3.8}^{ +7.3}$	7
	250–600	$8.78 \times 10^{-5}$	8.1	${}_{ -8.3}^{ +6.7}$	8
0.85–1.45	0–80	$2.25 \times 10^{-3}$	3.4	${}_{ -4.9}^{ +2.6}$	9
	80–150	$2.32 \times 10^{-3}$	3.3	${}_{ -2.8}^{ +4.8}$	10
	150–250	$8.85 \times 10^{-4}$	4.8	${}_{ -7.6}^{ +6.5}$	11
	250–600	$5.58 \times 10^{-5}$	9.6	${}_{ -9.9}^{+13.3}$	12
1.45–2.5	0–80	$9.08 \times 10^{-4}$	5.6	${}_{ -4.7}^{ +6.5}$	13
	80–150	$1.03 \times 10^{-3}$	4.5	${}_{ -6.3}^{ +7.3}$	14
	150–250	$3.14 \times 10^{-4}$	7.9	${}_{ -6.7}^{ +6.2}$	15
	250–600	$1.55 \times 10^{-5}$	17.3	${}_{-16.6}^{+12.7}$	16

**Table 6:** The correlation matrix of statistical uncertainties for the normalized $\mathrm{t}\overline{\mathrm{t}}$ double-differential cross sections as a function of $y(\mathrm{t})$ and $p_{\mathrm {T}} (\mathrm{t})$. The values are expressed as percentages. For bin indices see Table 5

Bin	1	2	3	4	5	6	7	8	9	10	11	12	13	14	15	16
1	$+100.0$	$-18.5$	$-16.4$	$+5.0$	$-31.8$	$-23.4$	$+8.6$	$-2.2$	$-46.5$	$+1.4$	$+7.5$	$-3.8$	$+11.3$	$+4.7$	$-7.9$	$+1.8$
2		$+100.0$	$-16.1$	$-4.1$	$-24.3$	$-10.6$	$-16.3$	$+5.2$	$-0.8$	$-22.5$	$+5.0$	$-0.3$	$+3.6$	$-1.4$	$-0.5$	$-1.1$
3			$+100.0$	$-35.2$	$+11.7$	$-17.4$	$-23.5$	$+1.8$	$+7.9$	$+4.6$	$-6.6$	$+4.2$	$-9.8$	$-0.9$	$+2.2$	$-1.4$
4				$+100.0$	$-1.7$	$+7.1$	$+2.0$	$-23.0$	$-3.7$	$+0.3$	$+3.5$	$+1.3$	$-1.2$	$-3.0$	$-1.2$	$+0.1$
5					$+100.0$	$-4.0$	$-19.0$	$+5.3$	$+30.9$	$-17.8$	$-3.4$	$+2.4$	$-46.9$	$-4.0$	$+10.3$	$-4.3$
6						$+100.0$	$+1.5$	$-9.3$	$-19.3$	$+19.3$	$-17.9$	$+4.1$	$-6.3$	$-25.3$	$+3.0$	$+1.2$
7							$+100.0$	$-29.9$	$-4.8$	$-16.5$	$-6.5$	$-3.5$	$+4.8$	$+0.5$	$-8.7$	$+4.6$
8								$+100.0$	$+1.8$	$+3.7$	$-1.6$	$-17.5$	$-5.2$	$-1.2$	$+2.9$	$+1.0$
9									$+100.0$	$-14.1$	$-18.3$	$+5.7$	$-19.6$	$-26.1$	$+7.0$	$-1.6$
10										$+100.0$	$-5.6$	$-7.2$	$-24.8$	$-4.3$	$-13.7$	$+5.7$
11											$+100.0$	$-34.1$	$+3.5$	$-15.0$	$-14.3$	$+0.7$
12												$+100.0$	$-3.1$	$+3.4$	$+2.7$	$-16.5$
13													$+100.0$	$-16.2$	$-29.8$	$+10.3$
14														$+100.0$	$-11.5$	$-7.6$
15															$+100.0$	$-43.7$
16																$+100.0$

**Table 7:** Sources and values of the relative systematic uncertainties in percent of the measured normalized $\mathrm{t}\overline{\mathrm{t}}$ double-differential cross sections as a function of $y(\mathrm{t})$ and $p_{\mathrm {T}} (\mathrm{t})$. For bin indices see Table 5

Syst. source\ Bin	1	2	3	4	5	6	7	8	9	10	11	12	13	14	15	16
Jet energy scale	${}^{ +2.7}_{ -0.5}$	${}^{ -1.9}_{ -1.0}$	${}^{ -0.1}_{ -0.5}$	${}^{ +2.6}_{ -1.4}$	${}^{ +0.8}_{ -1.5}$	${}^{ -1.4}_{ +0.3}$	${}^{ +2.3}_{ +0.8}$	${}^{ +0.3}_{ -3.8}$	${}^{ -1.8}_{ -1.0}$	${}^{ -1.9}_{ +2.6}$	${}^{ -2.7}_{ -1.0}$	${}^{ +3.8}_{ -1.8}$	${}^{ +2.1}_{ +3.0}$	${}^{ +1.7}_{ +1.2}$	${}^{ +0.6}_{ -1.1}$	${}^{ +1.5}_{ -5.0}$
Jet energy resolution	${}^{ +2.1}_{ +0.4}$	${}^{ -0.6}_{ -0.8}$	${}^{ -1.3}_{ -0.5}$	${}^{ +0.0}_{ -0.0}$	${}^{ -0.8}_{ -0.2}$	${}^{ -1.4}_{ -0.6}$	${}^{ +1.7}_{ +1.5}$	${}^{ -3.0}_{ +0.4}$	${}^{ -1.4}_{ -1.4}$	${}^{ +0.2}_{ +1.2}$	${}^{ +0.0}_{ -1.7}$	${}^{ -0.1}_{ +3.5}$	${}^{ +2.7}_{ +1.0}$	${}^{ +1.8}_{ +1.2}$	${}^{ -2.9}_{ -0.5}$	${}^{ +1.9}_{ -0.4}$
Kin. reconstruction	${}^{ +0.0}_{ -0.0}$	${}^{ +0.0}_{ -0.0}$	${}^{ -0.0}_{ +0.0}$	${}^{ -0.0}_{ +0.0}$	${}^{ -0.0}_{ +0.0}$	${}^{ -0.0}_{ +0.0}$	${}^{ -0.0}_{ +0.0}$	${}^{ -0.0}_{ +0.0}$	${}^{ +0.0}_{ +0.0}$	${}^{ -0.0}_{ +0.0}$	${}^{ +0.0}_{ -0.0}$	${}^{ -0.0}_{ +0.0}$	${}^{ +0.0}_{ -0.0}$	${}^{ +0.0}_{ -0.0}$	${}^{ -0.0}_{ +0.0}$	${}^{ +0.0}_{ -0.0}$
Pileup	${}^{ +0.0}_{ +0.0}$	${}^{ +0.1}_{ -0.2}$	${}^{ -0.2}_{ +0.3}$	${}^{ -0.3}_{ +0.1}$	${}^{ -0.1}_{ +0.1}$	${}^{ +0.0}_{ -0.1}$	${}^{ -0.4}_{ +0.5}$	${}^{ -0.4}_{ +0.4}$	${}^{ +0.1}_{ -0.1}$	${}^{ +0.4}_{ -0.5}$	${}^{ -0.4}_{ +0.5}$	${}^{ -0.5}_{ +0.5}$	${}^{ +0.2}_{ -0.1}$	${}^{ +0.0}_{ -0.0}$	${}^{ +0.2}_{ -0.1}$	${}^{ -0.8}_{ +0.9}$
Trigger	${}^{ +0.2}_{ -0.2}$	${}^{ +0.2}_{ -0.2}$	${}^{ +0.1}_{ -0.1}$	${}^{ +0.2}_{ -0.2}$	${}^{ +0.1}_{ -0.1}$	${}^{ +0.1}_{ -0.1}$	${}^{ +0.1}_{ -0.1}$	${}^{ +0.1}_{ -0.1}$	${}^{ -0.0}_{ +0.0}$	${}^{ -0.1}_{ +0.1}$	${}^{ +0.1}_{ -0.1}$	${}^{ +0.1}_{ -0.1}$	${}^{ -0.4}_{ +0.5}$	${}^{ -0.3}_{ +0.3}$	${}^{ -0.4}_{ +0.5}$	${}^{ -0.2}_{ +0.2}$
Background Z/$\gamma ^{*}$	${}^{ +0.0}_{ +0.0}$	${}^{ +0.4}_{ -0.4}$	${}^{ +0.4}_{ -0.4}$	${}^{ +0.2}_{ -0.2}$	${}^{ -0.4}_{ +0.5}$	${}^{ +0.1}_{ -0.1}$	${}^{ +0.2}_{ -0.2}$	${}^{ +0.4}_{ -0.4}$	${}^{ -0.2}_{ +0.2}$	${}^{ +0.1}_{ -0.1}$	${}^{ +0.3}_{ -0.4}$	${}^{ +0.6}_{ -0.6}$	${}^{ -0.6}_{ +0.7}$	${}^{ -0.2}_{ +0.1}$	${}^{ +0.3}_{ -0.3}$	${}^{ +0.5}_{ -0.5}$
Background other	${}^{ +0.0}_{ -0.0}$	${}^{ +0.2}_{ -0.2}$	${}^{ -0.1}_{ +0.1}$	${}^{ -0.2}_{ +0.1}$	${}^{ +0.0}_{ -0.1}$	${}^{ -0.1}_{ +0.0}$	${}^{ -0.2}_{ +0.3}$	${}^{ -0.9}_{ +0.9}$	${}^{ +0.2}_{ -0.2}$	${}^{ -0.1}_{ +0.2}$	${}^{ +0.3}_{ -0.4}$	${}^{ -0.4}_{ +0.5}$	${}^{ +0.0}_{ -0.0}$	${}^{ -0.0}_{ +0.1}$	${}^{ +0.1}_{ -0.0}$	${}^{ -0.2}_{ +0.2}$
b tagging	${}^{ +0.5}_{ -0.5}$	${}^{ +0.9}_{ -0.6}$	${}^{ +1.0}_{ -1.2}$	${}^{ +0.7}_{ -0.4}$	${}^{ +0.7}_{ -0.9}$	${}^{ +0.4}_{ -0.4}$	${}^{ +0.4}_{ -0.2}$	${}^{ +1.6}_{ -1.3}$	${}^{ +0.9}_{ -1.2}$	${}^{ +0.5}_{ -0.8}$	${}^{ +0.3}_{ -0.4}$	${}^{ +0.6}_{ -0.3}$	${}^{ +1.2}_{ -0.1}$	${}^{ +1.0}_{ -0.7}$	${}^{ +0.6}_{ -1.3}$	${}^{ +0.8}_{ -1.4}$
Int. luminosity	${}^{ +0.1}_{ -0.1}$	${}^{ +0.1}_{ -0.1}$	${}^{ -0.0}_{ +0.0}$	${}^{ -0.0}_{ +0.0}$	${}^{ -0.1}_{ +0.1}$	${}^{ -0.1}_{ +0.1}$	${}^{ -0.1}_{ +0.1}$	${}^{ -0.0}_{ +0.0}$	${}^{ -0.1}_{ +0.1}$	${}^{ -0.1}_{ +0.1}$	${}^{ +0.0}_{ -0.0}$	${}^{ +0.0}_{ -0.0}$	${}^{ +0.2}_{ -0.2}$	${}^{ +0.1}_{ -0.1}$	${}^{ -0.1}_{ +0.1}$	${}^{ +0.1}_{ -0.1}$
$m_{\mathrm{t}}$	${}^{ +0.7}_{ +0.1}$	${}^{ -1.0}_{ +0.9}$	${}^{ +0.3}_{ -0.2}$	${}^{ +2.1}_{ -3.3}$	${}^{ +0.4}_{ -0.6}$	${}^{ -0.6}_{ +1.0}$	${}^{ +0.4}_{ +0.1}$	${}^{ +2.2}_{ -2.9}$	${}^{ +0.1}_{ -0.7}$	${}^{ -0.9}_{ +1.1}$	${}^{ -0.1}_{ -0.4}$	${}^{ +2.8}_{ -2.2}$	${}^{ +0.3}_{ -0.1}$	${}^{ -0.5}_{ +0.6}$	${}^{ -0.3}_{ -1.2}$	${}^{ +4.2}_{ -4.3}$
$\mu _\mathrm {f}, \mu _\mathrm {r}$	${}^{ -2.7}_{ +4.2}$	${}^{ -4.2}_{ -2.2}$	${}^{ +0.2}_{ +0.7}$	${}^{ -6.7}_{ +4.9}$	${}^{ +0.0}_{ -0.8}$	${}^{ +0.9}_{ -1.7}$	${}^{ +4.6}_{ +0.2}$	${}^{ -2.2}_{ +1.7}$	${}^{ +1.9}_{ -3.4}$	${}^{ +1.9}_{ +0.9}$	${}^{ -3.5}_{ +2.4}$	${}^{ +3.1}_{ +5.9}$	${}^{ -1.4}_{ +2.1}$	${}^{ +3.3}_{ -1.2}$	${}^{ -1.3}_{ +0.8}$	${}^{ +1.5}_{ -4.4}$
Matching threshold	${}^{ +2.8}_{ +0.9}$	${}^{ -2.0}_{ -2.0}$	${}^{ +1.5}_{ +3.8}$	${}^{ -5.7}_{ -5.0}$	${}^{ -0.6}_{ +2.3}$	${}^{ -2.6}_{ -2.0}$	${}^{ +2.6}_{ -0.5}$	${}^{ +1.4}_{ +2.1}$	${}^{ -1.5}_{ -1.4}$	${}^{ +2.6}_{ +0.8}$	${}^{ +0.4}_{ +1.5}$	${}^{ +4.1}_{ +3.7}$	${}^{ -0.2}_{ -0.3}$	${}^{ +0.4}_{ -1.6}$	${}^{ -1.5}_{ +3.0}$	${}^{ +2.1}_{ -8.9}$
PDFs	${}^{ +0.6}_{ -0.7}$	${}^{ +0.1}_{ -0.1}$	${}^{ +0.2}_{ -0.2}$	${}^{ +0.2}_{ -0.2}$	${}^{ +0.2}_{ -0.2}$	${}^{ +0.1}_{ -0.1}$	${}^{ +0.1}_{ -0.1}$	${}^{ +0.3}_{ -0.3}$	${}^{ +0.5}_{ -0.4}$	${}^{ +0.3}_{ -0.3}$	${}^{ +0.2}_{ -0.2}$	${}^{ +0.5}_{ -0.5}$	${}^{ +0.6}_{ -0.6}$	${}^{ +0.2}_{ -0.1}$	${}^{ +0.3}_{ -0.3}$	${}^{ +0.4}_{ -0.4}$
Hadronization	$$ -3.3$$	$$ +0.4$$	$$ +2.7$$	$$ -5.9$$	$$ +1.7$$	$$ -1.8$$	$$ -2.4$$	$$ +2.2$$	$$ +1.5$$	$$ +0.6$$	$$ -5.2$$	$$ -1.1$$	$$ +0.0$$	$$ +4.9$$	$$ +1.7$$	$$ -4.3$$
Hard scattering	$$ +2.1$$	$$ -3.2$$	$$ +0.5$$	$$ +3.3$$	$$ +0.1$$	$$ -0.1$$	$$ +2.9$$	$$ -5.0$$	$$ -0.6$$	$$ +1.4$$	$$ +2.6$$	$$ -9.4$$	$$ +4.4$$	$$ -3.4$$	$$ -5.0$$	$$-10.5$$

**Table 8:** The measured normalized $\mathrm{t}\overline{\mathrm{t}}$ double-differential cross sections in different bins of $M(\mathrm{t}\overline{\mathrm{t}})$ and $y(\mathrm{t})$, along with their relative statistical and systematic uncertainties

$M(\mathrm{t}\overline{\mathrm{t}})$ ($\text {GeV}$ )	$|y(\mathrm{t}) |$	$\frac{1}{\sigma (\mathrm{t}\overline{\mathrm{t}})} \frac{\mathrm{d}^{2}\sigma (\mathrm{t}\overline{\mathrm{t}})}{\mathrm{d}M(\mathrm{t}\overline{\mathrm{t}}) \mathrm{d}y(\mathrm{t})}$ ($\text {GeV}$ $^{-1}$)	Stat. (%)	Syst. (%)	Bin
340–400	0–0.35	$3.21 \times 10^{-3}$	4.9	${}_{ -9.4}^{ +9.8}$	1
	0.35–0.85	$2.92 \times 10^{-3}$	4.0	${}_{ -5.7}^{ +3.9}$	2
	0.85–1.45	$2.06 \times 10^{-3}$	4.5	${}_{ -3.8}^{ +3.5}$	3
	1.45–2.5	$6.58 \times 10^{-4}$	9.3	${}_{ -5.8}^{ +7.4}$	4
400–500	0–0.35	$2.92 \times 10^{-3}$	3.2	${}_{ -8.3}^{ +7.4}$	5
	0.35–0.85	$2.39 \times 10^{-3}$	2.8	${}_{ -2.1}^{ +2.9}$	6
	0.85–1.45	$1.67 \times 10^{-3}$	3.2	${}_{ -4.2}^{ +3.4}$	7
	1.45–2.5	$5.99 \times 10^{-4}$	5.4	${}_{ -7.4}^{+10.8}$	8
500–650	0–0.35	$1.01 \times 10^{-3}$	5.0	${}_{ -7.7}^{ +1.8}$	9
	0.35–0.85	$8.73 \times 10^{-4}$	4.5	${}_{ -5.8}^{ +6.4}$	10
	0.85–1.45	$6.50 \times 10^{-4}$	4.8	${}_{ -5.6}^{ +6.5}$	11
	1.45–2.5	$2.91 \times 10^{-4}$	6.7	${}_{ -8.7}^{ +8.5}$	12
650–1500	0–0.35	$6.19 \times 10^{-5}$	7.8	${}_{-17.2}^{+19.6}$	13
	0.35–0.85	$6.77 \times 10^{-5}$	6.5	${}_{ -8.3}^{ +5.2}$	14
	0.85–1.45	$7.02 \times 10^{-5}$	5.4	${}_{ -4.7}^{ +6.3}$	15
	1.45–2.5	$4.42 \times 10^{-5}$	6.2	${}_{-14.2}^{ +9.1}$	16

**Table 9:** The correlation matrix of statistical uncertainties for the normalized $\mathrm{t}\overline{\mathrm{t}}$ double-differential cross sections as a function of $M(\mathrm{t}\overline{\mathrm{t}})$ and $y(\mathrm{t})$. The values are expressed as percentages. For bin indices see Table 8

Bin	1	2	3	4	5	6	7	8	9	10	11	12	13	14	15	16
1	$+100.0$	$-29.7$	$-31.0$	$+11.4$	$-21.1$	$-26.6$	$+0.9$	$+6.5$	$-16.8$	$+6.5$	$+6.6$	$-7.0$	$+5.8$	$+0.2$	$-3.8$	$-2.3$
2		$+100.0$	$+19.8$	$-35.7$	$-18.0$	$-6.1$	$-23.7$	$-3.6$	$+9.9$	$-20.9$	$-6.3$	$+10.0$	$-1.1$	$+5.5$	$+6.8$	$-4.7$
3			$+100.0$	$-19.4$	$+2.7$	$-16.6$	$-14.8$	$-28.3$	$+4.6$	$-4.2$	$-19.4$	$+1.3$	$-3.1$	$+1.3$	$+5.7$	$+3.1$
4				$+100.0$	$-2.2$	$-1.5$	$-13.8$	$-19.8$	$-9.4$	$+3.0$	$+3.9$	$-32.5$	$-1.1$	$-6.0$	$-6.5$	$+7.8$
5					$+100.0$	$-10.2$	$-24.0$	$-0.2$	$-10.0$	$-23.0$	$+0.6$	$+4.6$	$-10.1$	$+6.7$	$+3.4$	$-5.4$
6						$+100.0$	$+21.6$	$-25.3$	$-18.7$	$+8.8$	$-21.5$	$-5.3$	$+8.0$	$-16.0$	$-0.7$	$+3.7$
7							$+100.0$	$-5.9$	$+1.2$	$-9.8$	$+1.5$	$-24.1$	$+1.4$	$+0.8$	$-15.4$	$+3.3$
8								$+100.0$	$-0.7$	$-0.3$	$-8.8$	$+2.0$	$-2.9$	$-0.3$	$+5.3$	$-23.8$
9									$+100.0$	$-18.9$	$-10.4$	$+3.0$	$-35.2$	$-5.3$	$+5.4$	$-2.1$
10										$+100.0$	$+2.2$	$-14.1$	$+1.2$	$-27.6$	$-16.2$	$+4.2$
11											$+100.0$	$-8.6$	$+4.8$	$-3.6$	$-27.4$	$-14.2$
12												$+100.0$	$-2.6$	$+5.7$	$+3.8$	$-33.0$
13													$+100.0$	$-25.0$	$-1.2$	$+0.4$
14														$+100.0$	$-12.7$	$-4.2$
15															$+100.0$	$-22.0$
16																$+100.0$

**Table 10:** Sources and values of the relative systematic uncertainties in percent of the measured normalized $\mathrm{t}\overline{\mathrm{t}}$ double-differential cross sections as a function of $M(\mathrm{t}\overline{\mathrm{t}})$ and $y(\mathrm{t})$. For bin indices see Table 8

Syst. source/bin	1	2	3	4	5	6	7	8	9	10	11	12	13	14	15	16
Jet energy scale	${}^{ +2.9}_{ -2.9}$	${}^{ +0.3}_{ -1.4}$	${}^{ +1.8}_{ +0.5}$	${}^{ +2.4}_{ -0.4}$	${}^{ -0.8}_{ +1.1}$	${}^{ -1.1}_{ +1.5}$	${}^{ -3.5}_{ +1.3}$	${}^{ +0.1}_{ +2.0}$	${}^{ -0.6}_{ -0.5}$	${}^{ +1.7}_{ +0.5}$	${}^{ -0.7}_{ +0.6}$	${}^{ +1.6}_{ +1.2}$	${}^{ +4.6}_{ -2.1}$	${}^{ -0.5}_{ -2.6}$	${}^{ -0.7}_{ -1.1}$	${}^{ -1.7}_{ -5.0}$
Jet energy resolution	${}^{ +0.7}_{ +0.9}$	${}^{ -0.4}_{ -0.7}$	${}^{ -0.3}_{ -0.9}$	${}^{ +3.2}_{ +1.4}$	${}^{ -1.4}_{ -0.4}$	${}^{ +0.8}_{ +0.1}$	${}^{ -1.3}_{ -0.9}$	${}^{ +0.6}_{ +1.1}$	${}^{ -0.3}_{ -0.6}$	${}^{ +1.7}_{ +0.6}$	${}^{ +1.1}_{ +1.6}$	${}^{ +1.0}_{ -0.8}$	${}^{ +1.0}_{ +0.7}$	${}^{ -4.4}_{ +0.1}$	${}^{ +1.2}_{ -0.0}$	${}^{ -3.3}_{ -0.3}$
Kin. reconstruction	${}^{ +0.0}_{ -0.0}$	${}^{ -0.0}_{ +0.0}$	${}^{ -0.0}_{ +0.0}$	${}^{ +0.0}_{ -0.0}$	${}^{ +0.0}_{ -0.0}$	${}^{ -0.0}_{ +0.0}$	${}^{ +0.0}_{ -0.0}$	${}^{ +0.0}_{ -0.0}$	${}^{ -0.0}_{ +0.0}$	${}^{ -0.0}_{ +0.0}$	${}^{ -0.0}_{ +0.0}$	${}^{ -0.0}_{ +0.0}$	${}^{ +0.0}_{ -0.0}$	${}^{ +0.0}_{ -0.0}$	${}^{ +0.0}_{ -0.0}$	${}^{ +0.0}_{ -0.0}$
Pileup	${}^{ +0.4}_{ -0.6}$	${}^{ -0.3}_{ +0.2}$	${}^{ +0.1}_{ -0.1}$	${}^{ +0.6}_{ -0.7}$	${}^{ +0.0}_{ -0.1}$	${}^{ -0.1}_{ +0.1}$	${}^{ +0.2}_{ -0.1}$	${}^{ -0.3}_{ +0.6}$	${}^{ -0.3}_{ +0.3}$	${}^{ -0.4}_{ +0.6}$	${}^{ +0.2}_{ -0.2}$	${}^{ +0.5}_{ -0.7}$	${}^{ -0.0}_{ -0.0}$	${}^{ +0.2}_{ -0.3}$	${}^{ -0.4}_{ +0.3}$	${}^{ -0.4}_{ +0.5}$
Trigger	${}^{ +0.2}_{ -0.3}$	${}^{ +0.1}_{ -0.1}$	${}^{ -0.1}_{ +0.1}$	${}^{ -0.9}_{ +1.0}$	${}^{ +0.2}_{ -0.2}$	${}^{ +0.1}_{ -0.1}$	${}^{ +0.0}_{ -0.0}$	${}^{ -0.3}_{ +0.3}$	${}^{ +0.0}_{ -0.0}$	${}^{ +0.1}_{ -0.1}$	${}^{ -0.0}_{ +0.0}$	${}^{ -0.3}_{ +0.3}$	${}^{ +0.2}_{ -0.2}$	${}^{ +0.2}_{ -0.2}$	${}^{ +0.0}_{ -0.0}$	${}^{ -0.2}_{ +0.2}$
Background Z/$\gamma ^{*}$	${}^{ +0.2}_{ -0.2}$	${}^{ -0.8}_{ +0.8}$	${}^{ -0.7}_{ +0.6}$	${}^{ -0.7}_{ +1.0}$	${}^{ +0.2}_{ -0.3}$	${}^{ +0.2}_{ -0.2}$	${}^{ +0.3}_{ -0.3}$	${}^{ -0.1}_{ +0.1}$	${}^{ +0.2}_{ -0.3}$	${}^{ +0.2}_{ -0.2}$	${}^{ +0.3}_{ -0.3}$	${}^{ -0.4}_{ +0.4}$	${}^{ +0.4}_{ -0.4}$	${}^{ +0.3}_{ -0.4}$	${}^{ +0.3}_{ -0.3}$	${}^{ +0.4}_{ -0.4}$
Background other	${}^{ +0.3}_{ -0.3}$	${}^{ +0.2}_{ -0.2}$	${}^{ +0.4}_{ -0.4}$	${}^{ +0.3}_{ -0.3}$	${}^{ -0.1}_{ +0.0}$	${}^{ -0.2}_{ +0.2}$	${}^{ -0.1}_{ +0.1}$	${}^{ +0.0}_{ -0.0}$	${}^{ +0.0}_{ +0.0}$	${}^{ -0.3}_{ +0.3}$	${}^{ -0.2}_{ +0.1}$	${}^{ -0.1}_{ +0.0}$	${}^{ -0.3}_{ +0.3}$	${}^{ -0.0}_{ +0.0}$	${}^{ +0.4}_{ -0.4}$	${}^{ -0.1}_{ +0.2}$
b tagging	${}^{ +0.9}_{ -0.9}$	${}^{ +1.3}_{ -0.9}$	${}^{ +0.6}_{ -1.0}$	${}^{ +0.7}_{ -0.6}$	${}^{ +2.0}_{ -0.8}$	${}^{ +0.7}_{ -0.2}$	${}^{ +0.5}_{ -0.5}$	${}^{ +0.4}_{ -0.8}$	${}^{ +0.5}_{ -0.5}$	${}^{ +1.1}_{ -0.2}$	${}^{ +1.1}_{ -0.9}$	${}^{ +0.2}_{ -0.8}$	${}^{ +0.4}_{ -0.3}$	${}^{ +0.9}_{ -5.0}$	${}^{ +0.6}_{ -0.3}$	${}^{ +0.7}_{ -0.4}$
Int. luminosity	${}^{ +0.0}_{ -0.0}$	${}^{ -0.1}_{ +0.1}$	${}^{ -0.1}_{ +0.1}$	${}^{ +0.1}_{ -0.1}$	${}^{ +0.2}_{ -0.2}$	${}^{ +0.0}_{ -0.0}$	${}^{ +0.0}_{ -0.0}$	${}^{ +0.2}_{ -0.2}$	${}^{ -0.1}_{ +0.1}$	${}^{ -0.2}_{ +0.2}$	${}^{ -0.2}_{ +0.2}$	${}^{ -0.2}_{ +0.2}$	${}^{ +0.1}_{ -0.1}$	${}^{ +0.1}_{ -0.1}$	${}^{ +0.1}_{ -0.1}$	${}^{ +0.1}_{ -0.1}$
$m_{\mathrm{t}}$	${}^{ -2.5}_{ +3.2}$	${}^{ -2.1}_{ +1.4}$	${}^{ -2.5}_{ +1.5}$	${}^{ -2.7}_{ +2.3}$	${}^{ +0.4}_{ -0.3}$	${}^{ +0.3}_{ +0.5}$	${}^{ -0.5}_{ +0.3}$	${}^{ +0.6}_{ -0.0}$	${}^{ +1.2}_{ -2.2}$	${}^{ +1.7}_{ -1.2}$	${}^{ +1.6}_{ -1.0}$	${}^{ +0.3}_{ -0.5}$	${}^{ +4.1}_{ -3.5}$	${}^{ +3.6}_{ -3.1}$	${}^{ +2.4}_{ -2.0}$	${}^{ +1.0}_{ -2.6}$
$\mu _\mathrm {f}, \mu _\mathrm {r}$	${}^{ -5.0}_{ +4.7}$	${}^{ +0.4}_{ -1.0}$	${}^{ +1.7}_{ -1.9}$	${}^{ -4.0}_{ +4.6}$	${}^{ -0.1}_{ -3.3}$	${}^{ +2.1}_{ -1.1}$	${}^{ +2.0}_{ +1.4}$	${}^{ +7.4}_{ +0.7}$	${}^{ -7.2}_{ -0.3}$	${}^{ +1.5}_{ -0.6}$	${}^{ +0.4}_{ -0.4}$	${}^{ -5.1}_{ +2.8}$	${}^{ +1.3}_{ +8.4}$	${}^{ +0.8}_{ +1.5}$	${}^{ -0.1}_{ +0.0}$	${}^{ -2.9}_{ -6.3}$
Matching threshold	${}^{ +2.4}_{ +2.3}$	${}^{ -3.5}_{ +1.2}$	${}^{ +1.3}_{ -0.8}$	${}^{ -1.4}_{ +0.3}$	${}^{ -1.4}_{ +0.4}$	${}^{ -0.5}_{ -1.0}$	${}^{ +1.5}_{ +0.2}$	${}^{ +1.3}_{ +1.0}$	${}^{ -0.6}_{ -1.4}$	${}^{ +1.4}_{ -2.3}$	${}^{ -1.1}_{ +3.1}$	${}^{ +3.6}_{ -0.3}$	${}^{ -1.0}_{ -2.0}$	${}^{ +1.6}_{ +1.9}$	${}^{ +4.0}_{ +0.0}$	${}^{ -6.3}_{ -4.5}$
PDFs	${}^{ +1.0}_{ -1.1}$	${}^{ +0.2}_{ -0.3}$	${}^{ +0.7}_{ -0.7}$	${}^{ +1.1}_{ -1.1}$	${}^{ +0.2}_{ -0.2}$	${}^{ +0.1}_{ -0.2}$	${}^{ +0.2}_{ -0.2}$	${}^{ +0.2}_{ -0.3}$	${}^{ +0.2}_{ -0.2}$	${}^{ +0.2}_{ -0.2}$	${}^{ +0.3}_{ -0.3}$	${}^{ +0.4}_{ -0.4}$	${}^{ +0.5}_{ -0.5}$	${}^{ +0.2}_{ -0.2}$	${}^{ +0.3}_{ -0.3}$	${}^{ +0.2}_{ -0.2}$
Hadronization	$$ -6.0$$	$$ +1.4$$	$$ -0.6$$	$$ +0.7$$	$$ +3.7$$	$$ -0.7$$	$$ -0.8$$	$$ +7.2$$	$$ +0.6$$	$$ -3.6$$	$$ -3.2$$	$$ +6.7$$	$$ -1.6$$	$$ +2.6$$	$$ -3.3$$	$$ -3.9$$
Hard scattering	$$ +3.2$$	$$ -2.9$$	$$ +0.7$$	$$ +2.1$$	$$ -6.3$$	$$ +0.1$$	$$ +1.6$$	$$ -1.5$$	$$ -0.2$$	$$ +3.7$$	$$ +4.1$$	$$ +0.7$$	$$+16.5$$	$$ +1.1$$	$$ -2.2$$	$$ -8.1$$

**Table 11:** The measured normalized $\mathrm{t}\overline{\mathrm{t}}$ double-differential cross sections in different bins of $M(\mathrm{t}\overline{\mathrm{t}})$ and $y(\mathrm{t}\overline{\mathrm{t}})$, along with their relative statistical and systematic uncertainties

$M(\mathrm{t}\overline{\mathrm{t}})$ ($\text {GeV}$ )	$|y(\mathrm{t}\overline{\mathrm{t}}) |$	$\frac{1}{\sigma (\mathrm{t}\overline{\mathrm{t}})} \frac{\mathrm{d}^{2}\sigma (\mathrm{t}\overline{\mathrm{t}})}{\mathrm{d}M(\mathrm{t}\overline{\mathrm{t}}) \mathrm{d}y(\mathrm{t}\overline{\mathrm{t}})}$ ($\text {GeV}$ $^{-1}$)	Stat. (%)	Syst. (%)	Bin
340–400	0–0.35	$3.17 \times 10^{-3}$	4.5	${}_{ -5.9}^{ +6.6}$	1
	0.35–0.75	$3.07 \times 10^{-3}$	4.4	${}_{ -5.0}^{ +3.6}$	2
	0.75–1.15	$2.44 \times 10^{-3}$	5.1	${}_{ -5.1}^{ +6.1}$	3
	1.15–2.5	$9.14 \times 10^{-4}$	4.9	${}_{ -3.1}^{ +3.9}$	4
400–500	0–0.35	$3.06 \times 10^{-3}$	2.8	${}_{ -5.5}^{ +6.4}$	5
	0.35–0.75	$2.76 \times 10^{-3}$	2.8	${}_{ -5.4}^{ +4.7}$	6
	0.75–1.15	$2.05 \times 10^{-3}$	3.6	${}_{ -3.9}^{ +3.2}$	7
	1.15–2.5	$6.43 \times 10^{-4}$	3.6	${}_{ -5.4}^{ +6.0}$	8
500–650	0–0.35	$1.34 \times 10^{-3}$	3.8	${}_{ -4.8}^{ +2.1}$	9
	0.35–0.75	$1.17 \times 10^{-3}$	4.0	${}_{ -3.2}^{ +1.7}$	10
	0.75–1.15	$7.66 \times 10^{-4}$	5.8	${}_{ -5.0}^{ +4.7}$	11
	1.15–2.5	$1.85 \times 10^{-4}$	8.0	${}_{ -7.7}^{ +9.6}$	12
650–1500	0–0.35	$1.49 \times 10^{-4}$	4.2	${}_{ -6.8}^{ +3.6}$	13
	0.35–0.75	$1.18 \times 10^{-4}$	5.4	${}_{ -3.2}^{ +5.0}$	14
	0.75–1.15	$6.53 \times 10^{-5}$	8.3	${}_{ -7.5}^{+13.6}$	15
	1.15–2.5	$9.50 \times 10^{-6}$	17.2	${}_{-35.6}^{+33.9}$	16

**Table 12:** The correlation matrix of statistical uncertainties for the normalized $\mathrm{t}\overline{\mathrm{t}}$ double-differential cross sections as a function of $M(\mathrm{t}\overline{\mathrm{t}})$ and $y(\mathrm{t}\overline{\mathrm{t}})$. The values are expressed as percentages. For bin indices see Table 11

Bin	1	2	3	4	5	6	7	8	9	10	11	12	13	14	15	16
1	$+100.0$	$-23.2$	$-23.1$	$+5.4$	$-31.7$	$-19.6$	$+8.4$	$+0.6$	$-21.0$	$+5.6$	$+4.7$	$-7.2$	$+8.3$	$-0.8$	$-4.7$	$+0.4$
2		$+100.0$	$+7.6$	$-29.8$	$-17.6$	$-11.5$	$-24.6$	$+3.5$	$+6.5$	$-22.2$	$-2.5$	$+9.2$	$-0.5$	$+6.2$	$+3.7$	$-5.8$
3			$+100.0$	$-12.6$	$+7.8$	$-24.0$	$-19.9$	$-22.4$	$+4.2$	$-2.5$	$-19.2$	$+1.2$	$-4.3$	$+2.5$	$+6.6$	$+1.2$
4				$+100.0$	$-2.1$	$+2.1$	$-17.2$	$-24.4$	$-8.3$	$+4.9$	$+2.2$	$-38.7$	$-2.6$	$-7.1$	$-2.5$	$+13.1$
5					$+100.0$	$-5.2$	$-18.6$	$-4.1$	$-6.5$	$-18.7$	$+1.2$	$+1.3$	$-14.1$	$+3.9$	$+0.8$	$-3.4$
6						$+100.0$	$+17.6$	$-24.7$	$-18.9$	$+8.9$	$-17.7$	$-5.0$	$+5.3$	$-17.6$	$+1.5$	$+3.5$
7							$+100.0$	$-2.6$	$+0.3$	$-15.0$	$+0.3$	$-18.4$	$+1.2$	$+0.6$	$-13.5$	$+4.4$
8								$+100.0$	$+0.2$	$-4.7$	$-15.7$	$+9.5$	$-5.5$	$-0.4$	$+3.3$	$-24.0$
9									$+100.0$	$-13.2$	$-10.8$	$+1.3$	$-32.8$	$-6.9$	$+5.6$	$-2.0$
10										$+100.0$	$+2.4$	$-15.9$	$-5.5$	$-23.6$	$-12.6$	$+7.2$
11											$+100.0$	$-7.8$	$+4.8$	$-9.5$	$-27.9$	$-6.8$
12												$+100.0$	$-3.0$	$+4.4$	$-2.0$	$-38.9$
13													$+100.0$	$-21.1$	$-2.6$	$+0.6$
14														$+100.0$	$-15.7$	$-3.8$
15															$+100.0$	$-17.5$
16																$+100.0$

**Table 13:** Sources and values of the relative systematic uncertainties in percent of the measured normalized $\mathrm{t}\overline{\mathrm{t}}$ double-differential cross sections as a function of $M(\mathrm{t}\overline{\mathrm{t}})$ and $y(\mathrm{t}\overline{\mathrm{t}})$. For bin indices see Table 11

Syst. source\Bin	1	2	3	4	5	6	7	8	9	10	11	12	13	14	15	16
Jet energy scale	${}^{ +1.1}_{ -3.1}$	${}^{ +2.1}_{ -2.5}$	${}^{ +1.7}_{ -1.1}$	${}^{ +1.1}_{ +1.1}$	${}^{ +0.1}_{ +3.0}$	${}^{ -2.2}_{ +1.1}$	${}^{ -1.7}_{ +2.3}$	${}^{ -1.1}_{ +0.2}$	${}^{ -0.5}_{ -0.1}$	${}^{ -0.4}_{ +0.3}$	${}^{ -1.5}_{ +0.3}$	${}^{ +2.1}_{ -0.8}$	${}^{ -0.4}_{ -2.8}$	${}^{ +1.3}_{ -0.6}$	${}^{ +3.2}_{ -2.8}$	${}^{ +0.5}_{ -4.9}$
Jet energy resolution	${}^{ -0.8}_{ +0.1}$	${}^{ +1.0}_{ +0.7}$	${}^{ -0.3}_{ -0.8}$	${}^{ +0.3}_{ +0.5}$	${}^{ +1.0}_{ -0.2}$	${}^{ -0.7}_{ -0.3}$	${}^{ -0.2}_{ -0.3}$	${}^{ +0.3}_{ +0.2}$	${}^{ -0.6}_{ -0.1}$	${}^{ +0.1}_{ -0.5}$	${}^{ -0.3}_{ -0.1}$	${}^{ +2.8}_{ +0.5}$	${}^{ -0.4}_{ +0.5}$	${}^{ -0.9}_{ +1.1}$	${}^{ -1.1}_{ -1.3}$	${}^{ -3.6}_{ +0.1}$
Kin. reconstruction	${}^{ -0.0}_{ +0.0}$	${}^{ +0.0}_{ -0.0}$	${}^{ -0.0}_{ +0.0}$	${}^{ +0.0}_{ -0.0}$	${}^{ +0.0}_{ -0.0}$	${}^{ +0.0}_{ -0.0}$	${}^{ -0.0}_{ +0.0}$	${}^{ +0.0}_{ -0.0}$	${}^{ -0.0}_{ +0.0}$	${}^{ -0.0}_{ +0.0}$	${}^{ -0.0}_{ +0.0}$	${}^{ -0.0}_{ +0.0}$	${}^{ +0.0}_{ -0.0}$	${}^{ +0.0}_{ -0.0}$	${}^{ +0.0}_{ -0.0}$	${}^{ +0.0}_{ -0.0}$
Pileup	${}^{ +0.1}_{ -0.0}$	${}^{ +0.0}_{ -0.2}$	${}^{ +0.1}_{ -0.2}$	${}^{ +0.4}_{ -0.7}$	${}^{ +0.1}_{ -0.1}$	${}^{ -0.1}_{ +0.1}$	${}^{ +0.1}_{ -0.0}$	${}^{ -0.1}_{ +0.3}$	${}^{ -0.1}_{ +0.0}$	${}^{ -0.2}_{ +0.2}$	${}^{ +0.1}_{ -0.1}$	${}^{ -0.1}_{ +0.5}$	${}^{ -0.2}_{ -0.0}$	${}^{ -0.5}_{ +0.7}$	${}^{ +0.5}_{ -0.7}$	${}^{ -0.8}_{ +0.9}$
Trigger	${}^{ +0.2}_{ -0.2}$	${}^{ +0.2}_{ -0.2}$	${}^{ -0.1}_{ +0.1}$	${}^{ -0.5}_{ +0.5}$	${}^{ +0.2}_{ -0.2}$	${}^{ +0.2}_{ -0.2}$	${}^{ -0.0}_{ +0.0}$	${}^{ -0.3}_{ +0.3}$	${}^{ +0.1}_{ -0.1}$	${}^{ +0.0}_{ -0.0}$	${}^{ -0.1}_{ +0.1}$	${}^{ -0.5}_{ +0.5}$	${}^{ +0.1}_{ -0.1}$	${}^{ +0.1}_{ -0.1}$	${}^{ +0.1}_{ -0.1}$	${}^{ -0.1}_{ +0.1}$
Background Z/$\gamma ^{*}$	${}^{ +0.1}_{ -0.1}$	${}^{ -0.5}_{ +0.5}$	${}^{ -1.1}_{ +1.1}$	${}^{ -0.9}_{ +1.0}$	${}^{ +0.3}_{ -0.3}$	${}^{ +0.3}_{ -0.2}$	${}^{ +0.0}_{ -0.1}$	${}^{ +0.3}_{ -0.3}$	${}^{ +0.2}_{ -0.2}$	${}^{ +0.4}_{ -0.4}$	${}^{ +0.1}_{ -0.0}$	${}^{ -0.3}_{ +0.3}$	${}^{ +0.3}_{ -0.3}$	${}^{ +0.2}_{ -0.3}$	${}^{ +0.6}_{ -0.6}$	${}^{ +0.8}_{ -0.9}$
Background other	${}^{ +0.3}_{ -0.3}$	${}^{ +0.4}_{ -0.4}$	${}^{ +0.1}_{ -0.1}$	${}^{ +0.4}_{ -0.4}$	${}^{ -0.1}_{ +0.1}$	${}^{ -0.2}_{ +0.2}$	${}^{ -0.2}_{ +0.3}$	${}^{ +0.1}_{ -0.1}$	${}^{ -0.1}_{ +0.1}$	${}^{ +0.1}_{ -0.2}$	${}^{ -0.3}_{ +0.3}$	${}^{ -0.4}_{ +0.3}$	${}^{ +0.1}_{ -0.1}$	${}^{ -0.2}_{ +0.4}$	${}^{ -0.0}_{ -0.0}$	${}^{ -0.5}_{ +0.5}$
b tagging	${}^{ +0.7}_{ -0.7}$	${}^{ +1.1}_{ -1.2}$	${}^{ +0.6}_{ -1.2}$	${}^{ +0.8}_{ -0.3}$	${}^{ +1.2}_{ -0.4}$	${}^{ +0.7}_{ -0.3}$	${}^{ +0.3}_{ -0.1}$	${}^{ +0.4}_{ -1.0}$	${}^{ +0.6}_{ -0.3}$	${}^{ +0.9}_{ -0.5}$	${}^{ +0.1}_{ -0.5}$	${}^{ +0.4}_{ -1.3}$	${}^{ +0.4}_{ -2.2}$	${}^{ +0.2}_{ -0.1}$	${}^{ +1.5}_{ -1.2}$	${}^{ +0.5}_{ -0.6}$
Int. luminosity	${}^{ -0.0}_{ +0.0}$	${}^{ -0.0}_{ +0.0}$	${}^{ -0.4}_{ +0.4}$	${}^{ +0.1}_{ -0.1}$	${}^{ +0.1}_{ -0.1}$	${}^{ +0.1}_{ -0.1}$	${}^{ -0.1}_{ +0.1}$	${}^{ +0.4}_{ -0.4}$	${}^{ -0.2}_{ +0.1}$	${}^{ -0.2}_{ +0.2}$	${}^{ -0.3}_{ +0.3}$	${}^{ -0.3}_{ +0.3}$	${}^{ +0.1}_{ -0.1}$	${}^{ +0.1}_{ -0.1}$	${}^{ +0.2}_{ -0.2}$	${}^{ +0.3}_{ -0.3}$
$m_{\mathrm{t}}$	${}^{ -2.6}_{ +2.4}$	${}^{ -2.2}_{ +1.9}$	${}^{ -2.4}_{ +2.0}$	${}^{ -2.8}_{ +2.0}$	${}^{ +0.2}_{ +0.9}$	${}^{ -0.2}_{ +0.0}$	${}^{ +0.3}_{ +0.3}$	${}^{ +0.9}_{ -1.0}$	${}^{ +1.0}_{ -1.1}$	${}^{ +0.5}_{ -1.4}$	${}^{ +1.2}_{ -0.6}$	${}^{ +2.1}_{ -1.7}$	${}^{ +2.2}_{ -2.5}$	${}^{ +3.3}_{ -2.3}$	${}^{ +3.6}_{ -2.9}$	${}^{ +3.2}_{ -5.3}$
$\mu _\mathrm {f}, \mu _\mathrm {r}$	${}^{ -1.7}_{ +3.3}$	${}^{ -1.2}_{ +0.3}$	${}^{ +2.0}_{ -1.5}$	${}^{ +2.3}_{ -0.0}$	${}^{ +1.3}_{ -1.2}$	${}^{ +0.9}_{ -1.9}$	${}^{ -3.1}_{ +0.1}$	${}^{ +3.4}_{ +0.9}$	${}^{ +0.6}_{ -4.2}$	${}^{ -0.4}_{ -1.3}$	${}^{ -1.2}_{ +0.6}$	${}^{ -4.2}_{ +5.9}$	${}^{ -3.2}_{ -0.4}$	${}^{ -1.2}_{ +2.5}$	${}^{ +5.3}_{ +7.8}$	${}^{ -7.9}_{ -2.6}$
Matching threshold	${}^{ +1.7}_{ +3.0}$	${}^{ -2.7}_{ -1.1}$	${}^{ -0.5}_{ +3.4}$	${}^{ -0.3}_{ +1.0}$	${}^{ -0.1}_{ +0.5}$	${}^{ -0.4}_{ -0.2}$	${}^{ +1.6}_{ -0.2}$	${}^{ +1.3}_{ -2.0}$	${}^{ +0.0}_{ -1.2}$	${}^{ -1.8}_{ -0.1}$	${}^{ +1.0}_{ -1.3}$	${}^{ -0.8}_{ +2.0}$	${}^{ -2.0}_{ -3.1}$	${}^{ -0.6}_{ -0.4}$	${}^{ +8.2}_{ -1.1}$	${}^{ -0.7}_{ +1.8}$
PDFs	${}^{ +0.1}_{ -0.1}$	${}^{ +0.3}_{ -0.3}$	${}^{ +0.7}_{ -0.7}$	${}^{ +0.2}_{ -0.2}$	${}^{ +0.1}_{ -0.1}$	${}^{ +0.2}_{ -0.2}$	${}^{ +0.3}_{ -0.3}$	${}^{ +0.1}_{ -0.1}$	${}^{ +0.2}_{ -0.2}$	${}^{ +0.1}_{ -0.1}$	${}^{ +0.5}_{ -0.4}$	${}^{ +0.6}_{ -0.6}$	${}^{ +0.1}_{ -0.1}$	${}^{ +0.2}_{ -0.3}$	${}^{ +0.3}_{ -0.3}$	${}^{ +1.0}_{ -1.0}$
Hadronization	$$ -3.8$$	$$ +0.1$$	$$ +3.3$$	$$ -0.1$$	$$ +3.7$$	$$ -4.4$$	$$ -1.3$$	$$ +4.7$$	$$ +0.8$$	$$ -1.0$$	$$ -0.9$$	$$ +5.7$$	$$ -2.7$$	$$ -0.6$$	$$ +4.7$$	$$-18.8$$
Hard scattering	$$ -0.2$$	$$ -1.6$$	$$ -1.0$$	$$ -0.0$$	$$ -3.8$$	$$ +0.1$$	$$ -0.5$$	$$ -0.2$$	$$ -1.3$$	$$ +1.1$$	$$ +4.2$$	$$ +1.9$$	$$ -0.3$$	$$ +1.0$$	$$ +3.4$$	$$+27.9$$

**Table 14:** The measured normalized $\mathrm{t}\overline{\mathrm{t}}$ double-differential cross sections in different bins of $M(\mathrm{t}\overline{\mathrm{t}})$ and $\varDelta \eta (\mathrm{t},\overline{\mathrm{t}})$, along with their relative statistical and systematic uncertainties

$M(\mathrm{t}\overline{\mathrm{t}})$ ($\text {GeV}$ )	$\varDelta \eta (\mathrm{t},\overline{\mathrm{t}})$	$\frac{1}{\sigma (\mathrm{t}\overline{\mathrm{t}})} \frac{\mathrm{d}^{2}\sigma (\mathrm{t}\overline{\mathrm{t}})}{\mathrm{d}M(\mathrm{t}\overline{\mathrm{t}}) \mathrm{d}\varDelta \eta (\mathrm{t},\overline{\mathrm{t}})}$ ($\text {GeV}$ $^{-1}$)	Stat. (%)	Syst. (%)	Bin
340–400	0–0.4	$3.35 \times 10^{-3}$	7.3	${}_{ -7.5}^{ +9.7}$	1
	0.4–1.2	$2.53 \times 10^{-3}$	3.2	${}_{ -6.1}^{ +5.7}$	2
	1.2–6	$2.31 \times 10^{-4}$	8.4	${}_{ -9.5}^{+10.4}$	3
400–500	0–0.4	$1.60 \times 10^{-3}$	6.2	${}_{ -5.9}^{ +4.8}$	4
	0.4–1.2	$1.69 \times 10^{-3}$	3.3	${}_{ -3.0}^{ +3.9}$	5
	1.2–6	$3.87 \times 10^{-4}$	2.3	${}_{ -4.1}^{ +4.0}$	6
500–650	0–0.4	$3.56 \times 10^{-4}$	10.7	${}_{ -9.0}^{ +8.3}$	7
	0.4–1.2	$3.55 \times 10^{-4}$	7.7	${}_{ -9.8}^{+15.0}$	8
	1.2–6	$2.29 \times 10^{-4}$	2.4	${}_{ -6.2}^{ +2.9}$	9
650–1500	0–0.4	$2.08 \times 10^{-5}$	13.3	${}_{-11.4}^{ +8.8}$	10
	0.4–1.2	$2.42 \times 10^{-5}$	8.6	${}_{-12.9}^{ +9.1}$	11
	1.2–6	$2.31 \times 10^{-5}$	3.1	${}_{ -5.9}^{ +7.4}$	12

**Table 15:** The correlation matrix of statistical uncertainties for the normalized $\mathrm{t}\overline{\mathrm{t}}$ double-differential cross sections as a function of $M(\mathrm{t}\overline{\mathrm{t}})$ and $\varDelta \eta (\mathrm{t},\overline{\mathrm{t}})$. The values are expressed as percentages. For bin indices see Table 14

Bin	1	2	3	4	5	6	7	8	9	10	11	12
1	$+100.0$	$+1.8$	$-71.5$	$+2.0$	$-36.9$	$-15.9$	$-13.0$	$+3.4$	$+20.0$	$+3.5$	$-0.3$	$-11.8$
2		$+100.0$	$-13.1$	$-34.2$	$+6.4$	$-33.6$	$+15.6$	$-19.6$	$-13.1$	$-5.3$	$+3.5$	$+1.6$
3			$+100.0$	$+4.3$	$-2.1$	$-7.7$	$+5.8$	$+6.9$	$-33.8$	$-2.7$	$-1.0$	$+13.4$
4				$+100.0$	$-26.3$	$-25.5$	$-16.1$	$-24.2$	$+4.1$	$-0.3$	$+10.9$	$+2.0$
5					$+100.0$	$+5.4$	$-16.5$	$+6.3$	$-33.9$	$+8.2$	$-11.2$	$+1.4$
6						$+100.0$	$+10.5$	$-11.5$	$+3.4$	$-3.0$	$+5.6$	$-28.9$
7							$+100.0$	$-37.6$	$-3.1$	$-42.6$	$+6.0$	$+4.2$
8								$+100.0$	$-10.3$	$+9.6$	$-35.8$	$-7.7$
9									$+100.0$	$+2.2$	$+0.6$	$-40.7$
10										$+100.0$	$-31.1$	$+0.4$
11											$+100.0$	$-12.2$
12												$+100.0$

**Table 16:** Sources and values of the relative systematic uncertainties in percent of the measured normalized $\mathrm{t}\overline{\mathrm{t}}$ double-differential cross sections as a function of $M(\mathrm{t}\overline{\mathrm{t}})$ and $\varDelta \eta (\mathrm{t},\overline{\mathrm{t}})$. For bin indices see Table 14

Syst. source\ Bin	1	2	3	4	5	6	7	8	9	10	11	12
Jet energy scale	${}^{ +3.6}_{ -0.1}$	${}^{ +2.4}_{ -2.4}$	${}^{ -0.1}_{ -1.5}$	${}^{ -2.9}_{ +0.6}$	${}^{ -1.6}_{ +1.0}$	${}^{ -1.3}_{ +2.0}$	${}^{ +3.0}_{ +0.3}$	${}^{ +3.2}_{ -0.2}$	${}^{ -1.1}_{ +0.1}$	${}^{ +1.8}_{ -2.1}$	${}^{ +2.5}_{ -4.9}$	${}^{ -0.0}_{ -0.6}$
Jet energy resolution	${}^{ -1.3}_{ +0.8}$	${}^{ +0.2}_{ -0.7}$	${}^{ +1.1}_{ -0.0}$	${}^{ -1.4}_{ -1.2}$	${}^{ +1.6}_{ +0.2}$	${}^{ -0.4}_{ -0.0}$	${}^{ +1.2}_{ +2.2}$	${}^{ +0.5}_{ -1.0}$	${}^{ -0.4}_{ -0.6}$	${}^{ -1.1}_{ -1.4}$	${}^{ -2.4}_{ +1.3}$	${}^{ +0.4}_{ +1.7}$
Kin. reconstruction	${}^{ +0.0}_{ -0.0}$	${}^{ -0.0}_{ +0.0}$	${}^{ -0.0}_{ +0.0}$	${}^{ +0.0}_{ -0.0}$	${}^{ -0.0}_{ +0.0}$	${}^{ +0.0}_{ -0.0}$	${}^{ +0.0}_{ -0.0}$	${}^{ -0.1}_{ +0.1}$	${}^{ +0.0}_{ -0.0}$	${}^{ -0.0}_{ +0.0}$	${}^{ +0.0}_{ -0.0}$	${}^{ +0.0}_{ -0.0}$
Pileup	${}^{ +0.4}_{ -0.3}$	${}^{ -0.0}_{ -0.1}$	${}^{ +0.1}_{ -0.2}$	${}^{ +0.1}_{ -0.2}$	${}^{ -0.2}_{ +0.4}$	${}^{ +0.2}_{ -0.2}$	${}^{ -0.3}_{ +0.1}$	${}^{ -0.5}_{ +0.8}$	${}^{ +0.2}_{ -0.3}$	${}^{ -0.2}_{ +0.1}$	${}^{ +0.2}_{ -0.2}$	${}^{ -0.6}_{ +0.6}$
Trigger	${}^{ -0.0}_{ +0.0}$	${}^{ -0.1}_{ +0.1}$	${}^{ +0.1}_{ -0.1}$	${}^{ +0.2}_{ -0.2}$	${}^{ -0.1}_{ +0.1}$	${}^{ +0.1}_{ -0.1}$	${}^{ +0.3}_{ -0.3}$	${}^{ -0.2}_{ +0.2}$	${}^{ -0.0}_{ +0.0}$	${}^{ +0.0}_{ -0.0}$	${}^{ +0.3}_{ -0.3}$	${}^{ -0.1}_{ +0.1}$
Background Z/$\gamma ^{*}$	${}^{ -0.5}_{ +0.5}$	${}^{ -0.6}_{ +0.6}$	${}^{ -0.5}_{ +0.6}$	${}^{ +0.6}_{ -0.6}$	${}^{ +0.3}_{ -0.3}$	${}^{ +0.1}_{ -0.1}$	${}^{ +0.3}_{ -0.4}$	${}^{ +0.3}_{ -0.2}$	${}^{ +0.0}_{ -0.0}$	${}^{ +0.5}_{ -0.5}$	${}^{ +0.5}_{ -0.5}$	${}^{ +0.2}_{ -0.3}$
Background other	${}^{ +0.9}_{ -0.9}$	${}^{ +0.4}_{ -0.4}$	${}^{ -0.4}_{ +0.4}$	${}^{ -0.0}_{ +0.1}$	${}^{ -0.2}_{ +0.1}$	${}^{ -0.1}_{ +0.1}$	${}^{ -0.1}_{ +0.1}$	${}^{ -0.3}_{ +0.3}$	${}^{ +0.2}_{ -0.2}$	${}^{ -0.6}_{ +0.6}$	${}^{ +0.4}_{ -0.4}$	${}^{ -0.7}_{ +0.8}$
b tagging	${}^{ +0.2}_{ -0.2}$	${}^{ +1.4}_{ -1.0}$	${}^{ +0.3}_{ -1.9}$	${}^{ +1.3}_{ -0.6}$	${}^{ +0.8}_{ -0.6}$	${}^{ +0.6}_{ -0.3}$	${}^{ +0.2}_{ -0.2}$	${}^{ +0.7}_{ -1.6}$	${}^{ +1.5}_{ -0.8}$	${}^{ +0.3}_{ -1.3}$	${}^{ +0.9}_{ -0.9}$	${}^{ +0.8}_{ -0.1}$
Int. luminosity	${}^{ +0.4}_{ -0.4}$	${}^{ -0.4}_{ +0.4}$	${}^{ -0.1}_{ +0.1}$	${}^{ +0.4}_{ -0.4}$	${}^{ -0.2}_{ +0.2}$	${}^{ +0.2}_{ -0.2}$	${}^{ +0.2}_{ -0.2}$	${}^{ -0.4}_{ +0.4}$	${}^{ -0.0}_{ +0.0}$	${}^{ -0.1}_{ +0.1}$	${}^{ +0.2}_{ -0.2}$	${}^{ +0.0}_{ -0.0}$
$m_{\mathrm{t}}$	${}^{ -1.0}_{ +0.6}$	${}^{ -2.3}_{ +1.7}$	${}^{ -3.5}_{ +3.6}$	${}^{ +0.6}_{ -1.4}$	${}^{ +1.0}_{ -0.5}$	${}^{ -0.9}_{ +1.4}$	${}^{ +3.1}_{ -3.4}$	${}^{ +3.2}_{ -2.3}$	${}^{ +0.2}_{ -0.7}$	${}^{ +2.8}_{ -3.7}$	${}^{ +3.2}_{ -3.7}$	${}^{ +3.0}_{ -2.2}$
$\mu _\mathrm {f}, \mu _\mathrm {r}$	${}^{ -1.5}_{ -0.1}$	${}^{ -1.5}_{ +2.2}$	${}^{ +4.8}_{ +3.3}$	${}^{ -1.1}_{ +0.7}$	${}^{ +2.3}_{ -0.5}$	${}^{ +0.5}_{ -2.0}$	${}^{ -7.9}_{ +5.2}$	${}^{ +9.2}_{ +4.7}$	${}^{ -2.9}_{ -4.1}$	${}^{ +0.1}_{ -4.7}$	${}^{ -6.5}_{ +4.6}$	${}^{ +0.2}_{ +2.6}$
Matching threshold	${}^{ +2.8}_{ +5.3}$	${}^{ -1.8}_{ +0.7}$	${}^{ +2.2}_{ -1.6}$	${}^{ +0.3}_{ -0.0}$	${}^{ +0.1}_{ -0.7}$	${}^{ -0.6}_{ -0.7}$	${}^{ +3.4}_{ +2.6}$	${}^{ +5.5}_{ +3.3}$	${}^{ -3.3}_{ -0.9}$	${}^{ -0.3}_{ -4.7}$	${}^{ -6.1}_{ -1.6}$	${}^{ +2.9}_{ -1.7}$
PDFs	${}^{ +1.7}_{ -1.7}$	${}^{ +0.4}_{ -0.4}$	${}^{ +5.3}_{ -5.6}$	${}^{ +0.6}_{ -0.7}$	${}^{ +0.6}_{ -0.6}$	${}^{ +0.3}_{ -0.4}$	${}^{ +0.2}_{ -0.1}$	${}^{ +0.4}_{ -0.4}$	${}^{ +0.3}_{ -0.2}$	${}^{ +0.3}_{ -0.3}$	${}^{ +0.4}_{ -0.5}$	${}^{ +0.2}_{ -0.2}$
Hadronization	$$ +6.8$$	$$ -4.2$$	$$ -3.3$$	$$ +3.8$$	$$ -1.1$$	$$ +1.9$$	$$ -0.8$$	$$ -5.4$$	$$ +1.0$$	$$ -8.0$$	$$ +2.2$$	$$ -1.3$$
Hard scattering	$$ -0.0$$	$$ +0.0$$	$$ +5.1$$	$$ +2.4$$	$$ -1.8$$	$$ -2.4$$	$$ -2.3$$	$$ +7.6$$	$$ -2.6$$	$$ -0.2$$	$$ -6.2$$	$$ +4.9$$

**Table 17:** The measured normalized $\mathrm{t}\overline{\mathrm{t}}$ double-differential cross sections in different bins of $M(\mathrm{t}\overline{\mathrm{t}})$ and $p_{\mathrm {T}} (\mathrm{t}\overline{\mathrm{t}})$, along with their relative statistical and systematic uncertainties

$M(\mathrm{t}\overline{\mathrm{t}})$ ($\text {GeV}$ )	$p_{\mathrm {T}} (\mathrm{t}\overline{\mathrm{t}})$ ($\text {GeV}$ )	$\frac{1}{\sigma (\mathrm{t}\overline{\mathrm{t}})} \frac{\mathrm{d}^{2}\sigma (\mathrm{t}\overline{\mathrm{t}})}{\mathrm{d}M(\mathrm{t}\overline{\mathrm{t}}) \mathrm{d}p_{\mathrm {T}} (\mathrm{t}\overline{\mathrm{t}})}$ ($\text {GeV}$ $^{-2}$)	Stat. (%)	Syst. (%)	Bin
340–400	0–30	$6.63 \times 10^{-5}$	3.0	${}_{ -4.0}^{ +6.7}$	1
	30–75	$3.51 \times 10^{-5}$	3.5	${}_{ -7.5}^{ +5.9}$	2
	75–150	$9.61 \times 10^{-6}$	6.6	${}_{-10.8}^{+13.1}$	3
	150–500	$6.19 \times 10^{-7}$	14.8	${}_{-17.2}^{ +8.6}$	4
400–500	0–30	$5.57 \times 10^{-5}$	2.0	${}_{ -6.0}^{ +6.7}$	5
	30–75	$2.82 \times 10^{-5}$	2.5	${}_{ -5.5}^{ +5.4}$	6
	75–150	$8.34 \times 10^{-6}$	4.7	${}_{-10.9}^{+14.3}$	7
	150–500	$9.02 \times 10^{-7}$	7.6	${}_{ -7.5}^{ +6.9}$	8
500–650	0–30	$2.09 \times 10^{-5}$	3.4	${}_{-11.1}^{ +9.0}$	9
	30–75	$1.11 \times 10^{-5}$	4.1	${}_{ -7.4}^{ +7.7}$	10
	75–150	$3.24 \times 10^{-6}$	7.1	${}_{-16.6}^{+14.3}$	11
	150–500	$2.91 \times 10^{-7}$	11.8	${}_{ -3.0}^{ +6.9}$	12
650–1500	0–30	$1.68 \times 10^{-6}$	5.8	${}_{ -7.8}^{+10.0}$	13
	30–75	$1.09 \times 10^{-6}$	6.4	${}_{-14.1}^{+10.9}$	14
	75–150	$3.80 \times 10^{-7}$	7.8	${}_{ -8.2}^{+10.4}$	15
	150–500	$3.96 \times 10^{-8}$	9.2	${}_{-16.7}^{+18.3}$	16

**Table 18:** The correlation matrix of statistical uncertainties for the normalized $\mathrm{t}\overline{\mathrm{t}}$ double-differential cross sections as a function of $M(\mathrm{t}\overline{\mathrm{t}})$ and $p_{\mathrm {T}} (\mathrm{t}\overline{\mathrm{t}})$. The values are expressed as percentages. For bin indices see Table 17

Bin	1	2	3	4	5	6	7	8	9	10	11	12	13	14	15	16
1	$+100.0$	$-20.4$	$-22.7$	$+1.5$	$-25.9$	$-21.0$	$+8.9$	$-1.5$	$-37.8$	$+5.1$	$+6.6$	$-4.3$	$+6.9$	$-1.4$	$-7.9$	$-0.2$
2		$+100.0$	$+5.3$	$-10.6$	$-26.3$	$-17.3$	$-21.5$	$+7.5$	$+4.0$	$-30.1$	$+0.9$	$-0.1$	$+0.6$	$+4.5$	$+2.2$	$-3.1$
3			$+100.0$	$-8.5$	$+6.4$	$-32.2$	$-36.0$	$-3.0$	$+9.5$	$-6.0$	$-17.9$	$+5.8$	$-5.7$	$+5.4$	$+8.6$	$-3.4$
4				$+100.0$	$-0.2$	$+8.2$	$-9.0$	$-59.8$	$-4.0$	$+1.2$	$+3.5$	$+7.5$	$-0.1$	$-2.2$	$-1.1$	$+1.6$
5					$+100.0$	$-7.6$	$-22.5$	$-1.6$	$+13.7$	$-24.7$	$-0.8$	$+1.3$	$-26.9$	$+7.1$	$+2.0$	$-4.1$
6						$+100.0$	$+18.0$	$-13.9$	$-28.2$	$+19.3$	$-16.3$	$+5.1$	$+8.8$	$-24.1$	$+3.0$	$-0.2$
7							$+100.0$	$+1.2$	$-5.8$	$-17.1$	$-13.4$	$-6.5$	$+4.9$	$+0.4$	$-10.5$	$+4.7$
8								$+100.0$	$+1.3$	$-0.3$	$-8.2$	$-43.8$	$-1.6$	$+0.1$	$+2.2$	$+5.1$
9									$+100.0$	$-17.3$	$-17.5$	$+2.1$	$-25.9$	$-6.9$	$+9.7$	$-3.0$
10										$+100.0$	$+4.9$	$-8.7$	$-9.1$	$-15.4$	$-12.3$	$+6.3$
11											$+100.0$	$-7.5$	$+7.4$	$-12.6$	$-32.0$	$+2.9$
12												$+100.0$	$-1.0$	$+1.4$	$-3.3$	$-42.9$
13													$+100.0$	$-38.8$	$-5.1$	$+1.6$
14														$+100.0$	$-19.4$	$-4.3$
15															$+100.0$	$-18.6$
16																$+100.0$

**Table 19:** Sources and values of the relative systematic uncertainties in percent of the measured normalized $\mathrm{t}\overline{\mathrm{t}}$ double-differential cross sections as a function of $M(\mathrm{t}\overline{\mathrm{t}})$ and $p_{\mathrm {T}} (\mathrm{t}\overline{\mathrm{t}})$. For bin indices see Table 17

Syst. source/bin	1	2	3	4	5	6	7	8	9	10	11	12	13	14	15	16
Jet energy scale	${}^{ +4.3}_{ -1.7}$	${}^{ -0.3}_{ -1.3}$	${}^{ -0.1}_{ +0.3}$	${}^{ +6.4}_{ +0.9}$	${}^{ -0.6}_{ -0.4}$	${}^{ -2.3}_{ +2.2}$	${}^{ -2.5}_{ +3.1}$	${}^{ -4.5}_{ +0.4}$	${}^{ -0.3}_{ -0.8}$	${}^{ +1.5}_{ +0.9}$	${}^{ -1.5}_{ +3.4}$	${}^{ -0.5}_{ +3.5}$	${}^{ +2.4}_{ -1.8}$	${}^{ +0.4}_{ -4.5}$	${}^{ -1.2}_{ -0.6}$	${}^{ +0.5}_{ -0.7}$
Jet energy resolution	${}^{ +1.1}_{ +0.1}$	${}^{ -0.7}_{ -0.7}$	${}^{ -1.0}_{ +0.5}$	${}^{ +2.0}_{ -0.2}$	${}^{ -0.3}_{ -1.7}$	${}^{ -0.2}_{ +1.0}$	${}^{ +1.6}_{ +1.9}$	${}^{ -1.7}_{ -2.2}$	${}^{ -0.2}_{ -1.6}$	${}^{ +0.4}_{ +4.0}$	${}^{ +0.1}_{ +0.4}$	${}^{ +2.5}_{ +2.9}$	${}^{ +0.1}_{ +0.4}$	${}^{ -1.3}_{ -1.5}$	${}^{ -0.2}_{ -1.4}$	${}^{ -1.4}_{ +1.9}$
Kin. reconstruction	${}^{ -0.0}_{ +0.0}$	${}^{ +0.0}_{ -0.0}$	${}^{ -0.0}_{ +0.0}$	${}^{ -0.0}_{ +0.0}$	${}^{ +0.0}_{ -0.0}$	${}^{ -0.0}_{ +0.0}$	${}^{ +0.0}_{ -0.0}$	${}^{ +0.0}_{ -0.0}$	${}^{ -0.0}_{ +0.0}$	${}^{ -0.0}_{ +0.0}$	${}^{ -0.0}_{ +0.0}$	${}^{ -0.0}_{ +0.0}$	${}^{ +0.0}_{ -0.0}$	${}^{ +0.0}_{ -0.0}$	${}^{ +0.0}_{ -0.0}$	${}^{ -0.0}_{ +0.0}$
Pileup	${}^{ +0.5}_{ -0.6}$	${}^{ -0.5}_{ +0.5}$	${}^{ +0.5}_{ -0.4}$	${}^{ -0.8}_{ -0.4}$	${}^{ +0.4}_{ -0.3}$	${}^{ -0.3}_{ +0.4}$	${}^{ +0.0}_{ +0.1}$	${}^{ -0.5}_{ +0.7}$	${}^{ +0.5}_{ -0.4}$	${}^{ -0.6}_{ +0.5}$	${}^{ -0.6}_{ +0.6}$	${}^{ -0.7}_{ +0.9}$	${}^{ +0.8}_{ -0.7}$	${}^{ -0.9}_{ +0.6}$	${}^{ +0.1}_{ -0.4}$	${}^{ -0.7}_{ +0.7}$
Trigger	${}^{ -0.0}_{ +0.0}$	${}^{ -0.0}_{ +0.0}$	${}^{ +0.0}_{ -0.0}$	${}^{ -0.2}_{ +0.2}$	${}^{ +0.1}_{ -0.1}$	${}^{ -0.0}_{ +0.0}$	${}^{ +0.1}_{ -0.1}$	${}^{ +0.2}_{ -0.2}$	${}^{ -0.1}_{ +0.1}$	${}^{ -0.1}_{ +0.1}$	${}^{ -0.1}_{ +0.1}$	${}^{ -0.1}_{ +0.1}$	${}^{ +0.0}_{ -0.0}$	${}^{ +0.1}_{ -0.1}$	${}^{ -0.0}_{ -0.0}$	${}^{ -0.0}_{ +0.1}$
Background Z/$\gamma ^{*}$	${}^{ -0.8}_{ +0.8}$	${}^{ -0.2}_{ +0.2}$	${}^{ +0.6}_{ -0.6}$	${}^{ -2.1}_{ +2.4}$	${}^{ +0.1}_{ -0.1}$	${}^{ +0.1}_{ -0.2}$	${}^{ +0.1}_{ -0.1}$	${}^{ +0.1}_{ -0.2}$	${}^{ +0.3}_{ -0.3}$	${}^{ -0.1}_{ +0.1}$	${}^{ -0.1}_{ +0.0}$	${}^{ +0.5}_{ -0.6}$	${}^{ +0.2}_{ -0.3}$	${}^{ +0.3}_{ -0.3}$	${}^{ +0.4}_{ -0.4}$	${}^{ +0.3}_{ -0.3}$
Background other	${}^{ +0.0}_{ -0.0}$	${}^{ +0.5}_{ -0.5}$	${}^{ +0.8}_{ -0.8}$	${}^{ -0.6}_{ +0.7}$	${}^{ -0.2}_{ +0.2}$	${}^{ -0.1}_{ +0.2}$	${}^{ +0.1}_{ -0.1}$	${}^{ -0.5}_{ +0.4}$	${}^{ -0.1}_{ +0.0}$	${}^{ -0.1}_{ +0.1}$	${}^{ -0.4}_{ +0.4}$	${}^{ -1.1}_{ +1.1}$	${}^{ -0.1}_{ +0.1}$	${}^{ +1.0}_{ -1.0}$	${}^{ +0.1}_{ -0.2}$	${}^{ -1.4}_{ +1.4}$
b tagging	${}^{ +0.5}_{ -0.5}$	${}^{ +0.4}_{ -0.6}$	${}^{ +0.8}_{ -1.9}$	${}^{ +2.6}_{ -2.4}$	${}^{ +0.8}_{ -0.3}$	${}^{ +0.1}_{ -0.5}$	${}^{ +0.6}_{ -0.8}$	${}^{ +1.1}_{ -0.0}$	${}^{ +1.4}_{ -0.3}$	${}^{ +0.3}_{ -0.8}$	${}^{ +0.9}_{ -0.7}$	${}^{ +1.1}_{ -2.3}$	${}^{ +0.5}_{ -0.0}$	${}^{ +0.4}_{ -1.4}$	${}^{ +0.9}_{ -1.6}$	${}^{ +0.1}_{ -0.6}$
Int. luminosity	${}^{ -0.0}_{ +0.0}$	${}^{ -0.1}_{ +0.1}$	${}^{ +0.1}_{ -0.1}$	${}^{ -0.4}_{ +0.4}$	${}^{ +0.2}_{ -0.2}$	${}^{ +0.0}_{ -0.0}$	${}^{ +0.1}_{ -0.1}$	${}^{ +0.1}_{ -0.1}$	${}^{ -0.2}_{ +0.2}$	${}^{ -0.3}_{ +0.3}$	${}^{ -0.2}_{ +0.2}$	${}^{ -0.1}_{ +0.1}$	${}^{ +0.2}_{ -0.2}$	${}^{ +0.3}_{ -0.3}$	${}^{ +0.1}_{ -0.1}$	${}^{ -0.1}_{ +0.1}$
$m_{\mathrm{t}}$	${}^{ -2.9}_{ +2.3}$	${}^{ -2.6}_{ +1.5}$	${}^{ -1.2}_{ +2.5}$	${}^{ -1.6}_{ +3.1}$	${}^{ +0.7}_{ -0.4}$	${}^{ +0.3}_{ -0.2}$	${}^{ -1.0}_{ +1.8}$	${}^{ -1.6}_{ +0.4}$	${}^{ +1.6}_{ -1.4}$	${}^{ +0.6}_{ -0.9}$	${}^{ +1.5}_{ -2.2}$	${}^{ +1.9}_{ -0.5}$	${}^{ +3.2}_{ -3.3}$	${}^{ +3.3}_{ -2.7}$	${}^{ +1.5}_{ -1.6}$	${}^{ +1.8}_{ -0.8}$
$\mu _\mathrm {f}, \mu _\mathrm {r}$	${}^{ -1.2}_{ +3.8}$	${}^{ -0.3}_{ -3.8}$	${}^{ +7.0}_{ -2.1}$	${}^{-10.4}_{ -1.9}$	${}^{ -2.8}_{ +4.4}$	${}^{ +2.4}_{ -2.3}$	${}^{+11.6}_{ -8.1}$	${}^{ -1.4}_{ -0.8}$	${}^{ -8.3}_{ +5.1}$	${}^{ +3.8}_{ -5.0}$	${}^{ -0.4}_{ -6.4}$	${}^{ +4.4}_{ +3.1}$	${}^{ -4.2}_{ +7.3}$	${}^{ +1.0}_{ -5.2}$	${}^{ +2.6}_{ +1.3}$	${}^{ +0.4}_{ +5.0}$
Matching threshold	${}^{ -1.3}_{ +1.9}$	${}^{ +0.3}_{ -0.3}$	${}^{ +1.7}_{ +1.6}$	${}^{-12.7}_{ -0.3}$	${}^{ +0.1}_{ +0.3}$	${}^{ -0.4}_{ -1.5}$	${}^{ +2.4}_{ +0.2}$	${}^{ +4.4}_{ +1.7}$	${}^{ +1.4}_{ +1.8}$	${}^{ -2.1}_{ +1.1}$	${}^{ -5.1}_{ -6.2}$	${}^{ +1.2}_{ +1.2}$	${}^{ -0.6}_{ +1.4}$	${}^{ -1.4}_{ -5.8}$	${}^{ +7.2}_{ -3.3}$	${}^{ +5.1}_{ +3.5}$
PDFs	${}^{ +0.2}_{ -0.3}$	${}^{ +0.2}_{ -0.2}$	${}^{ +0.1}_{ -0.2}$	${}^{ +0.2}_{ -0.2}$	${}^{ +0.2}_{ -0.3}$	${}^{ +0.1}_{ -0.1}$	${}^{ +0.2}_{ -0.2}$	${}^{ +0.4}_{ -0.4}$	${}^{ +0.1}_{ -0.1}$	${}^{ +0.3}_{ -0.3}$	${}^{ +0.5}_{ -0.5}$	${}^{ +0.4}_{ -0.4}$	${}^{ +0.4}_{ -0.3}$	${}^{ +0.5}_{ -0.4}$	${}^{ +0.2}_{ -0.2}$	${}^{ +0.6}_{ -0.6}$
Hadronization	$$ +0.2$$	$$ -5.0$$	$$+10.1$$	$$ -3.0$$	$$ +4.8$$	$$ -2.5$$	$$ -5.4$$	$$ +2.7$$	$$ +6.7$$	$$ -3.3$$	$$-13.1$$	$$ +0.8$$	$$ -4.6$$	$$+10.1$$	$$ -5.2$$	$$-10.4$$
Hard scattering	$$ -0.3$$	$$ -2.7$$	$$ -2.1$$	$$ -1.5$$	$$ -1.4$$	$$ -3.2$$	$$ +4.1$$	$$ -4.2$$	$$ +2.1$$	$$ +3.5$$	$$ +4.2$$	$$ -0.3$$	$$ +2.5$$	$$ -1.1$$	$$ +4.4$$	$$+12.8$$

**Table 20:** The measured normalized $\mathrm{t}\overline{\mathrm{t}}$ double-differential cross sections in different bins of $M(\mathrm{t}\overline{\mathrm{t}})$ and $\varDelta \phi (\mathrm{t},\overline{\mathrm{t}})$, along with their relative statistical and systematic uncertainties

$M(\mathrm{t}\overline{\mathrm{t}})$ ($\text {GeV}$ )	$\varDelta \phi (\mathrm{t},\overline{\mathrm{t}})$ (rad)	$\frac{1}{\sigma (\mathrm{t}\overline{\mathrm{t}})} \frac{\mathrm{d}^{2}\sigma (\mathrm{t}\overline{\mathrm{t}})}{\mathrm{d}M(\mathrm{t}\overline{\mathrm{t}}) \mathrm{d}\varDelta \phi (\mathrm{t},\overline{\mathrm{t}})}$ ($\text {GeV}$ $^{-1}$ rad$^{-1}$)	Stat. (%)	Syst. (%)	Bin
340–400	0–2.2	$5.68 \times 10^{-4}$	5.5	${}_{ -8.1}^{+10.1}$	1
	2.2–2.95	$2.68 \times 10^{-3}$	3.3	${}_{ -6.4}^{ +5.3}$	2
	2.95–$\pi$	$6.67 \times 10^{-3}$	5.6	${}_{-13.7}^{+22.1}$	3
400–500	0–2.2	$2.59 \times 10^{-4}$	5.9	${}_{-10.2}^{+12.1}$	4
	2.2–2.95	$2.09 \times 10^{-3}$	2.5	${}_{ -6.8}^{ +5.2}$	5
	2.95–$\pi$	$8.90 \times 10^{-3}$	2.3	${}_{ -3.3}^{ +4.9}$	6
500–650	0–2.2	$7.04 \times 10^{-5}$	11.0	${}_{-16.0}^{+19.6}$	7
	2.2–2.95	$7.23 \times 10^{-4}$	4.4	${}_{-13.8}^{+10.5}$	8
	2.95–$\pi$	$4.11 \times 10^{-3}$	2.9	${}_{ -7.4}^{ +7.0}$	9
650–1500	0–2.2	$6.06 \times 10^{-6}$	13.3	${}_{-22.1}^{+16.8}$	10
	2.2–2.95	$6.40 \times 10^{-5}$	6.0	${}_{ -8.3}^{+10.5}$	11
	2.95–$\pi$	$4.21 \times 10^{-4}$	3.6	${}_{ -5.2}^{ +4.1}$	12

**Table 21:** The correlation matrix of statistical uncertainties for the normalized $\mathrm{t}\overline{\mathrm{t}}$ double-differential cross sections as a function of $M(\mathrm{t}\overline{\mathrm{t}})$ and $\varDelta \phi (\mathrm{t},\overline{\mathrm{t}})$. The values are expressed as percentages. For bin indices see Table 20

Bin	1	2	3	4	5	6	7	8	9	10	11	12
1	$+100.0$	$-2.3$	$-44.6$	$-29.0$	$-31.3$	$+4.7$	$-15.6$	$-5.0$	$+11.8$	$+7.6$	$+5.1$	$-6.0$
2		$+100.0$	$-24.4$	$-18.1$	$+2.6$	$-43.1$	$+6.8$	$-30.5$	$+2.1$	$-2.8$	$+5.2$	$+2.6$
3			$+100.0$	$+16.1$	$-24.6$	$-11.2$	$+3.4$	$+11.4$	$-30.7$	$-5.4$	$-3.5$	$+5.1$
4				$+100.0$	$-2.5$	$-18.5$	$-25.5$	$-19.7$	$-1.2$	$-4.4$	$+2.3$	$+2.9$
5					$+100.0$	$-10.5$	$-8.8$	$+14.5$	$-33.5$	$+4.1$	$-23.5$	$+6.2$
6						$+100.0$	$+4.9$	$-20.4$	$+6.9$	$-2.3$	$+7.1$	$-22.7$
7							$+100.0$	$-7.5$	$-9.2$	$-39.9$	$-9.2$	$+4.5$
8								$+100.0$	$-18.5$	$+0.6$	$-29.5$	$-10.3$
9									$+100.0$	$+3.7$	$-0.0$	$-31.9$
10										$+100.0$	$-18.3$	$-3.3$
11											$+100.0$	$-23.0$
12												$+100.0$

**Table 22:** Sources and values of the relative systematic uncertainties in percent of the measured normalized $\mathrm{t}\overline{\mathrm{t}}$ double-differential cross sections as a function of $M(\mathrm{t}\overline{\mathrm{t}})$ and $\varDelta \phi (\mathrm{t},\overline{\mathrm{t}})$. For bin indices see Table 20

Syst. source/bin	1	2	3	4	5	6	7	8	9	10	11	12
Jet energy scale	${}^{ +1.9}_{ +0.9}$	${}^{ +0.5}_{ -2.4}$	${}^{ +2.8}_{ -2.9}$	${}^{ -1.5}_{ +3.3}$	${}^{ -2.7}_{ +2.4}$	${}^{ +0.3}_{ +0.2}$	${}^{ -1.8}_{ +0.8}$	${}^{ -2.5}_{ +2.3}$	${}^{ +0.9}_{ -0.5}$	${}^{ -6.9}_{ -3.1}$	${}^{ +3.9}_{ -1.2}$	${}^{ +1.2}_{ -3.0}$
Jet energy resolution	${}^{ +1.1}_{ +2.1}$	${}^{ -0.8}_{ +0.4}$	${}^{ -0.9}_{ -2.6}$	${}^{ -2.1}_{ -1.7}$	${}^{ +0.3}_{ -0.1}$	${}^{ +0.7}_{ -0.0}$	${}^{ +5.5}_{ +4.0}$	${}^{ -0.4}_{ +0.3}$	${}^{ +0.1}_{ -0.3}$	${}^{ -3.6}_{ -4.8}$	${}^{ +0.5}_{ +2.4}$	${}^{ -1.0}_{ -0.1}$
Kin. reconstruction	${}^{ +0.0}_{ -0.0}$	${}^{ -0.0}_{ +0.0}$	${}^{ -0.0}_{ +0.0}$	${}^{ +0.0}_{ -0.0}$	${}^{ -0.0}_{ +0.0}$	${}^{ +0.0}_{ -0.0}$	${}^{ +0.0}_{ -0.0}$	${}^{ -0.0}_{ +0.0}$	${}^{ -0.0}_{ +0.0}$	${}^{ -0.0}_{ +0.0}$	${}^{ +0.0}_{ -0.0}$	${}^{ +0.0}_{ -0.0}$
Pileup	${}^{ -0.2}_{ +0.1}$	${}^{ -0.2}_{ +0.1}$	${}^{ +1.1}_{ -1.3}$	${}^{ +0.1}_{ -0.0}$	${}^{ -0.4}_{ +0.4}$	${}^{ +0.4}_{ -0.3}$	${}^{ -1.0}_{ +1.3}$	${}^{ -0.6}_{ +0.5}$	${}^{ +0.2}_{ -0.1}$	${}^{ -0.9}_{ +0.8}$	${}^{ +0.1}_{ -0.2}$	${}^{ -0.0}_{ -0.0}$
Trigger	${}^{ +0.0}_{ -0.0}$	${}^{ -0.0}_{ +0.0}$	${}^{ -0.1}_{ +0.1}$	${}^{ +0.0}_{ -0.0}$	${}^{ -0.0}_{ +0.0}$	${}^{ +0.1}_{ -0.1}$	${}^{ +0.1}_{ -0.1}$	${}^{ -0.1}_{ +0.2}$	${}^{ -0.0}_{ +0.0}$	${}^{ -0.3}_{ +0.3}$	${}^{ +0.0}_{ +0.0}$	${}^{ +0.1}_{ -0.1}$
Background Z/$\gamma ^{*}$	${}^{ -1.0}_{ +1.0}$	${}^{ -0.5}_{ +0.5}$	${}^{ -0.4}_{ +0.5}$	${}^{ -0.2}_{ +0.1}$	${}^{ +0.3}_{ -0.3}$	${}^{ +0.2}_{ -0.2}$	${}^{ +1.0}_{ -0.7}$	${}^{ +0.2}_{ -0.2}$	${}^{ +0.1}_{ -0.1}$	${}^{ +0.4}_{ -0.3}$	${}^{ +0.2}_{ -0.2}$	${}^{ +0.4}_{ -0.4}$
Background other	${}^{ +0.6}_{ -0.6}$	${}^{ +0.3}_{ -0.3}$	${}^{ +0.1}_{ -0.1}$	${}^{ -0.8}_{ +0.7}$	${}^{ -0.1}_{ +0.1}$	${}^{ +0.0}_{ -0.0}$	${}^{ -0.5}_{ +0.4}$	${}^{ -0.4}_{ +0.4}$	${}^{ +0.0}_{ -0.0}$	${}^{ -1.0}_{ +0.9}$	${}^{ +0.2}_{ -0.1}$	${}^{ +0.1}_{ -0.1}$
b tagging	${}^{ +0.9}_{ -0.9}$	${}^{ +1.7}_{ -0.7}$	${}^{ +0.4}_{ -0.7}$	${}^{ +0.3}_{ -0.7}$	${}^{ +1.6}_{ -0.2}$	${}^{ +0.3}_{ -0.4}$	${}^{ +0.4}_{ -2.6}$	${}^{ +0.8}_{ -0.4}$	${}^{ +0.5}_{ -0.4}$	${}^{ +0.5}_{ -1.5}$	${}^{ +1.4}_{ -0.8}$	${}^{ +0.3}_{ -0.4}$
Int. luminosity	${}^{ +0.2}_{ -0.2}$	${}^{ -0.3}_{ +0.3}$	${}^{ +0.1}_{ -0.1}$	${}^{ -0.0}_{ +0.0}$	${}^{ -0.1}_{ +0.1}$	${}^{ +0.3}_{ -0.3}$	${}^{ +0.1}_{ -0.1}$	${}^{ -0.4}_{ +0.4}$	${}^{ -0.1}_{ +0.1}$	${}^{ -0.1}_{ +0.1}$	${}^{ +0.2}_{ -0.2}$	${}^{ +0.2}_{ -0.2}$
$m_{\mathrm{t}}$	${}^{ -0.5}_{ -0.9}$	${}^{ -1.8}_{ +1.7}$	${}^{ -5.0}_{ +4.6}$	${}^{ +0.5}_{ +0.9}$	${}^{ +0.4}_{ +0.0}$	${}^{ -0.1}_{ +0.1}$	${}^{ +0.3}_{ +1.4}$	${}^{ -0.2}_{ -0.8}$	${}^{ +2.0}_{ -1.9}$	${}^{ +1.9}_{ -1.3}$	${}^{ +2.3}_{ -1.8}$	${}^{ +3.1}_{ -3.2}$
$\mu _\mathrm {f}, \mu _\mathrm {r}$	${}^{ +9.2}_{ -7.7}$	${}^{ -3.3}_{ -2.4}$	${}^{-11.5}_{+21.0}$	${}^{ +9.6}_{ -7.6}$	${}^{ +2.5}_{ -4.4}$	${}^{ -1.7}_{ +3.7}$	${}^{ -0.7}_{ +4.8}$	${}^{ +6.9}_{ -9.2}$	${}^{ -5.4}_{ +3.8}$	${}^{ +1.5}_{-10.5}$	${}^{ +4.0}_{ -2.6}$	${}^{ -2.0}_{ +2.2}$
Matching threshold	${}^{ +1.8}_{ -1.0}$	${}^{ -0.8}_{ +2.4}$	${}^{ -1.7}_{ +2.3}$	${}^{ +1.2}_{ +0.4}$	${}^{ -1.2}_{ -2.2}$	${}^{ +1.1}_{ +1.1}$	${}^{ +4.3}_{ +9.1}$	${}^{ -2.0}_{ -6.5}$	${}^{ +0.5}_{ +2.5}$	${}^{ -4.5}_{ -5.5}$	${}^{ +4.5}_{ -2.8}$	${}^{ -1.5}_{ -0.8}$
PDFs	${}^{ +0.2}_{ -0.2}$	${}^{ +2.1}_{ -2.2}$	${}^{ +1.1}_{ -1.2}$	${}^{ +0.3}_{ -0.3}$	${}^{ +0.4}_{ -0.4}$	${}^{ +0.1}_{ -0.1}$	${}^{ +0.5}_{ -0.4}$	${}^{ +0.2}_{ -0.2}$	${}^{ +0.1}_{ -0.1}$	${}^{ +0.4}_{ -0.3}$	${}^{ +0.3}_{ -0.2}$	${}^{ +0.1}_{ -0.1}$
Hadronization	$$ +1.0$$	$$ -3.6$$	$$ +2.5$$	$$ +3.4$$	$$ -2.7$$	$$ +2.7$$	$$+14.1$$	$$ -7.4$$	$$ +3.0$$	$$-14.7$$	$$ +1.2$$	$$ -0.5$$
Hard scattering	$$ +0.8$$	$$ -1.1$$	$$ +1.8$$	$$ -5.1$$	$$ -2.7$$	$$ +0.3$$	$$ -6.6$$	$$ +1.3$$	$$ +3.7$$	$$ -7.6$$	$$ +6.9$$	$$ +0.2$$

## Appendix B: PDF fit of single-differential $\mathrm{t}\overline{\mathrm{t}}$ measurement at NNLO

Approximate NNLO predictions [19] for the $y(\mathrm{t})$ single-differential cross section are obtained using the DiffTop program, which is interfaced to fastNLO  [90] (version 2.1). The results are used in a PDF fit at NNLO. The procedure follows the determination of the PDFs at NLO described in Sect. 9.1. In the NNLO fit, the scales for $\mathrm{t}\overline{\mathrm{t}}$ production are set to $\mu _\mathrm {r} = \mu _\mathrm {f} = m_{\mathrm{t}}$, with $m_{\mathrm{t}} = 173$ $\,\text {GeV}$ being the top quark pole mass. The scale evolution of partons is calculated through the DGLAP equations at NNLO. The DIS and $\mathrm {W}^\pm$ boson charge asymmetry theoretical predictions are calculated at NNLO accuracy. For the $\mathrm {W}^\pm$ boson charge asymmetry predictions, the NNLO corrections are obtained by using K-factors, defined as the ratios of the predictions at NNLO to the ones at NLO, both calculated with the fewz  [91] program (version 3.1), using the NNLO CT10 [32] PDFs. As in Ref. [63], the charm quark mass parameter is set to $M_{\mathrm{c}}= 1.43$ $\,\text {GeV}$ for a fit at NNLO. To stabilise for the comparison, the fit of the gluon distribution at NNLO, which suffers from insufficient constraints when using the inclusive HERA DIS and $\mathrm {W}^\pm$ boson charge asymmetry data alone, the $Q^2$ range of the HERA data is further restricted to $Q^2 > Q^2_\text {min} = 7.5$ $\,\text {GeV}$ $^2$. In addition, a reduced set of 15 parameters is used for the PDFs, which are parametrized at the initial scale of the QCD evolution as:

8 $$\begin{aligned} x\mathrm{g} (x)= & {} A_{\mathrm{g}} x^{B_{\mathrm{g}}}\,(1-x)^{C_{\mathrm{g}}}\, - A'_{\mathrm{g}} x^{B'_{\mathrm{g}}}\,(1-x)^{C'_{\mathrm{g}}}, \nonumber \\ x\mathrm{u}_v(x)= & {} A_{\mathrm{u}_v}x^{B_{\mathrm{u}_v}}\,(1-x)^{C_{\mathrm{u}_v}}\,(1+E_{\mathrm{u}_v}x^2) ,\nonumber \\ x\mathrm{d}_v(x)= & {} A_{\mathrm{d}_v}x^{B_{\mathrm{d}_v}}\,(1-x)^{C_{\mathrm{d}_v}},\nonumber \\ x\overline{U}(x)= & {} A_{\overline{U}}x^{B_{\overline{U}}}\,(1-x)^{C_{\overline{U}}}\, (1+E_{\overline{U}}x^{2}),\nonumber \\ x\overline{D}(x)= & {} A_{\overline{D}}x^{B_{\overline{D}}}\,(1-x)^{C_{\overline{D}}}\, (1+E_{\overline{D}}x^{2}). \end{aligned}$$

The PDF uncertainty estimation follows the NLO fit procedure described in Sect. 9.1, except for the model parameter variations of $5 \le Q^2_\mathrm{{min}}\le 10\,\text {GeV} ^2$ and $1.37\le M_{\mathrm{c}}\le 1.49\,\text {GeV}$. The resulting gluon distribution at a scale of $\mu _\mathrm{{f}}^2=30{,} 000\,\text {GeV} ^2 \simeq m_{\mathrm{t}}^2$ is shown in Fig. 19, together with its uncertainty band. The reduction of the total gluon PDF uncertainty is noticeable at large *x*, once the $\mathrm{t}\overline{\mathrm{t}}$ cross sections are included in the fit. This impact is smaller compared to the one observed in the 18-parameter fit at NLO (Fig. 17).
